# Exploration of neuropeptides to identify potential target for regulating feeding behavior and development in *Eurygaster integriceps*

**DOI:** 10.1371/journal.pone.0353952

**Published:** 2026-07-17

**Authors:** Mehrbanoo Kazemi Alamouti, Mohammad Majdi, Reza Talebi, Mehdi Dastranj, Aziz Sheikhigarjan, Mohsen Mardi, Alireza Bandani, Zahra Ghorbanzadeh, Ghasem Hosseini Salekdeh, Mohammad Reza Ghaffari

**Affiliations:** 1 Department of Systems and Synthetic Biology, Agricultural Biotechnology Research Institute of Iran, Agricultural Research, Education and Extension Organization (AREEO), Karaj, Iran; 2 Department of Plant Genetics and Production, Faculty of Agriculture, University of Kurdistan, Sanandaj, Iran; 3 Department of Animal Genomics and Bioinformatics, Animal Biotechnology Research Institute, Agricultural Research, Education and Extension Organization (AREEO), Agricultural Biotechnology Research Institute of Iran (ABRII), Rasht, Iran; 4 Department of Agricultural Entomology, Iranian Research Institute of Plant Protection, Agricultural Research, Education and Extension Organization (AREEO), Tehran, Iran; 5 Department of Plant Protection, College of Agriculture and Natural Resources, University of Tehran, Karaj, Iran; 6 Department of Bioinformatics, Agricultural Biotechnology Research Institute of Iran, Agricultural Research, Education and Extension Organization (AREEO), Karaj, Iran; 7 School of Natural Sciences, Macquarie University, Macquarie Park, NSW, New Southwest Wales; Charles University: Univerzita Karlova, CZECHIA

## Abstract

Neuropeptides regulate diverse physiological processes in insects, including feeding, reproduction, and development, and have therefore emerged as promising species-specific targets for next-generation pest control strategies. In this study, we focused on 13 feeding-related neuropeptides previously identified from our whole-body RNA-seq dataset of *Eurygaster integriceps*. Using a combination of bioinformatics and expression analyses, we characterized precursor structures, predicted mature peptides, and quantified expression across developmental stages, between sexes, and in key feeding-associated organs of head and gut. Several neuropeptides, including *Ast-A*, *Ast-B*, *Ast-C*, *AKH*, *SIF*, *ITP*, *Burs*, *sNPF*, *NPF*, and *Crz* showed higher expression in males than in females, suggesting sexually dimorphic regulation of feeding and metabolism. Moreover, most neuropeptides were expressed at higher levels in the head compared to the gut, which is consistent with central neuro-regulatory functions. Furthermore the expression pattern of neuropeptides of life cycle developmental stages revealed peak expression in early developmental stages, along with stage-specific variation indicating coordinated regulation of growth, feeding behavior, and digestive physiology. Taken together, these findings provide the first detailed molecular and expression atlas of feeding-related neuropeptides in *E. integriceps* and offer foundational insights for developing future neuropeptide-based, species-specific, and environmentally safe biocontrol strategies.

## Introduction

The Sunn pest, *Eurygaster integriceps*, is a major agricultural pest of wheat and barley in the West and Central Asia and Eastern Europe, causing devastating economic losses [1]. Yield losses attributed to heavy infestations of Sunn pest in two key crops ranges from 50–90% (wheat) and 20–30% (barley) with the possibility of complete crop failure without any effective pest control [2,3]. For instance, in Iran, infestations lead to over 9 million tons of qualitative and quantitative losses annually, severely impacting food security and economic stability [4]. These severe losses further highlight the critical need for targeted pest management strategies to mitigate the damage caused by *E. integriceps*. Current management of Sunn pest relies on chemical, biological, and cultural strategies, such as insecticides, natural enemies, and resistant wheat varieties [1]. However, these approaches face limitations, including environmental concerns and dependence on precise knowledge of the pest’s seasonal behavior. Importantly, molecular-level information, such as the developmental and tissue-specific expression of feeding-related neuropeptides, is lacking, representing a critical knowledge gap for developing more targeted and sustainable control tools.

One promising avenue involves disrupting the insect’s fundamental physiological processes. Insects have various strategies to obtain their required nutrients and energy [5] of which homeostasis is vital for survival, growth, and reproduction of animal species [6]. Energy deprivation drives a complex behavioral program to ensure adequate food intake [7]. Feeding behavior in insects has been investigated in terms of physiology, biology and biochemistry [5]. Physiological aspects of feeding behavior indicate that special components of the nervous system are involved in regulating feeding behavior in insects. Neuropeptides can be defined as regulatory peptides with functional roles in growth and development, behavior and reproduction, metabolism, homeostasis, and muscle movement [8,9]. They can be produced in most major types of neurons, including sensory cells/neurons, interneurons, and motoneurons. Hormonal peptides are produced both in neurosecretory cells of the central nervous system (CNS) and in peripheral secretory (neurosecretory and endocrine) cells, hence signaling can occur bi-directionally from the CNS to periphery and vice versa [10–12]. Due to the high specificity of neuropeptides and their cognate receptors, they can be used to design a selective insecticidal target system for reducing the fitness of target pest insects, while minimizing detrimental environmental impacts [13,14].

For instance, NPF regulates feeding similar to vertebrate NPY. Neuropeptides, such as CCHamide and Bursicon, govern food intake, sensory perception, cuticle remodeling, and seasonal transitions. Given their essential and highly specific roles, neuropeptides and their receptors have emerged as promising targets for the development of next-generation, species-specific insecticides. Molecular identification and characterization of these signaling molecules provide critical insights into the pathways that modulate insect physiology and behavior [15]. Although neuropeptide systems have been investigated in several agricultural pests, information remains scarce or incomplete for many species, including *E. integriceps*, highlighting the need for molecular-level studies to improve our understanding and advance innovative pest management strategies

Our earlier whole-body RNA-seq study [16] identified 46 neuropeptide precursors in *Eurygaster integriceps*, but it did not resolve developmental, sex-specific, or tissue-specific expression patterns. Building on this foundational dataset, the present study focuses on major feeding-related neuropeptides for detailed structural and functional analysis. We examined precursor architecture, predicted mature peptide structures, and quantified expression across developmental stages, between sexes, and in key feeding-associated tissues (head and gut) using gene expression profiling. By integrating precursor structure with validated expression patterns, this research delivers the first comprehensive expression profile of feeding-related neuropeptides in *E. integriceps* and provides new molecular insight into the neuropeptidergic network underlying feeding behavior and development.

## Materials and methods

### Transcriptome data mining and peptide structural analysis

The transcriptomic resources used for peptide discovery and structural characterization in this study were derived from our previously published whole-body RNA-seq dataset of *E. integriceps* [16], available under BioProject accession PRJNA1015108. Building on this existing dataset, we performed targeted in silico mining of neuropeptide genes with a specific emphasis on 13 neuropeptides that are strongly implicated in feeding regulation and gut–brain signaling, based on extensive literature evidence. These 13 candidates represent a subset of the 46 neuropeptide precursors catalogued in our earlier work and were selected for detailed structural and expression analyses in the present study. Prediction of precursor architecture and mature peptide structure followed an established bioinformatic workflow [17,18]. Putative prohormone cleavage sites were annotated according to Veenstra’s criteria [19] and by comparison with known neuropeptide processing patterns. Signal peptides were identified using SignalP 5 (http://www.cbs.dtu.dk/services/SignalP/), and domain organization of precursors was visualized using Domain Graph (DOG, version 2.0). Multiple sequence alignments were conducted with ClustalX 2.1, and conserved motifs along with sequence logo representations were generated using WebLogo 2.8.2 [20]. Post-translational modifications, including N-terminal cyclization (Q/E) and C-terminal amidation from terminal glycine residues, were inferred based on sequence homology to established peptide isoforms [21]. This integrative data-mining and structural-prediction approach enabled precise annotation of the selected neuropeptide precursors and their predicted mature peptides, forming the basis for subsequent developmental, sex-specific, and tissue-specific expression analyses in this study.

### Insect rearing and sample preparation

In early April 2023, adult *E. integriceps* specimens were collected from wheat fields at the Varamin Research field, Tehran Province, Iran (35.3252° N, 51.6472° E). The collected population was reared on freshly cut wheat and provided with water in a growth chamber at the Department of Systems Biology, Agricultural Biotechnology Research Institute of Iran (ABRII), Karaj, Alborz Province, Iran. The chamber was maintained under controlled conditions with a long-day photoperiod (16 h light/8 h dark), a temperature of 26 ± 1 °C, and relative humidity of 60 ± 10% [4].

The developmental stages of *E. integriceps* included eggs, 1st, 2nd, 3rd, 4th, and 5th instar nymphs, as well as female and male adults. For each stage three independent biological replicates were prepared. Each biological replicate consisted of a separate pooled sample of multiple individuals, including more than 100 eggs, 30 individuals for each of the 1st to 3rd instar nymphs, and 10 individuals for each of the 4th and 5th instar nymphs, as well as female and male adults. Samples were immediately frozen in liquid nitrogen and stored at −80 °C until further experiments.

For organ-specific analysis, female and male adults were dissected to isolate heads and guts. For each tissue type, three independent biological replicates were prepared, each consisting of pooled samples (15 heads or 15 guts per replicate). Adult Sunn pests were immobilized on a clean wax plate using insect pins, and their epidermis was carefully removed. The dissected organs were placed in sterilized glass containers, rinsed twice with DEPC-treated water, and separated using microscopic tweezers under a stereomicroscope (Nikon SMZ1500, Japan). The pooled organs were pulverized in liquid nitrogen and stored at −80 °C prior to RNA extraction.

### Quantitative real-time PCR (qPCR) analysis

Total RNA was extracted from various *E. integriceps* samples using TRIzol reagent (Invitrogen, Carlsbad, CA, USA) according to the manufacturer’s protocol. The purity and integrity of the extracted RNA were evaluated using a NanoDrop 2000 spectrophotometer (Thermo Scientific, Wilmington, DE, USA). To eliminate genomic DNA contamination, the RNA samples were treated with DNase I (Fermentas, Madison, WI, USA). Reverse transcription was performed using the PrimeScript RT Reagent Kit (Takara, Dalian, Liaoning, China) in a 20 μL reaction mixture containing 1 μg of total RNA. The resulting cDNA was serially diluted 2-fold for the analysis of RT-qPCR primer specificity. Quantitative primers were designed using Oligo7 software (Version Oligo7: v7.60) [22] and Primer3 (Version v2.6.1) based on the obtained nucleotide sequences. Primer sequences are provided in S1 Table). RT-qPCR analysis was conducted on a Light Cycler 480 system (Roche, Switzerland) using a two-step amplification protocol: 95°C for 30 seconds, followed by 40 cycles of 95°C for 5 seconds and 60°C for 30 seconds. Melting curve analysis was performed to confirm primer specificity. Each reaction mix consisted of 5.0 μL of SinaSYBR Green QPCR Master Mix (Sinaclon), 3.9 μL of nuclease-free water, 0.6 μL of forward and reverse primers (10 μmol/L), and 0.5 μL of cDNA. The *18S rRNA* gene (KP890857) was used as an internal reference [23]. Two reference genes, *Actin* and *18S rRNA*, were initially tested for their expression stability in this study. Due to limited genomic information for this species, these genes were selected as commonly used references. Stability analysis of *18S rRNA* was performed, including calculation of mean, standard deviation (SD), standard error of the mean (SEM), and coefficient of variation (CV%). The quantification cycle (Cq) values were automatically calculated using the Second Derivative Maximum Method implemented in the Roche LightCycler analysis software. Each experiment included three biological replicates and two technical replicates. Relative mRNA expression levels were calculated using the 2^−ΔΔCT^ method [24].

### Statistical analysis

The relative gene expression levels (ΔCq values normalized to reference genes) were calculated for each sample, and results are presented as mean ± standard error (SE). Pairwise comparisons between two groups (male vs. female, and head vs. gut) were performed using independent-samples t-tests. For comparisons involving more than two groups (developmental stages), one-way analysis of variance (ANOVA) was conducted, followed by Tukey’s honestly significant difference (HSD) post hoc test. Statistical significance was set at P < 0.05. All analyses were performed using SPSS version 27.0 (IBM Corp., Armonk, NY, USA).

## Results

### Neuropeptides discovery by transcriptome mining: bioinformatics analyses and peptide prediction

**Fig 1:**
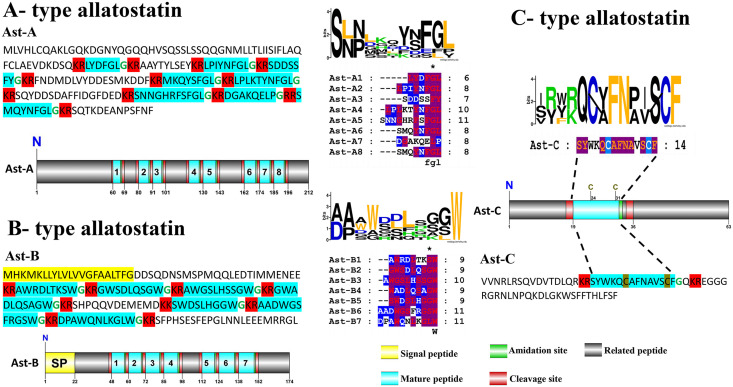
Identification and characterization of *E. integriceps* ASTs peptides. (A-type allatostatin) Schematic representation of Ast-A precursor and their 8 mature peptides comparative sequence alignments in *E. integriceps*, sequence logo is shown above alignments. (B-type allatostatin) Amino acid sequences and schematic representation of Ast-B precursor in *E. integriceps*, logo is shown above comparative sequence alignments of Ast-B peptides. (C-type allatostatin) Schematic representation of Ast-C in *E. integriceps* and their mature peptides comparative sequence alignments, sequence logo is shown above alignments*.*

Through transcriptome mining and bioinformatics analyses, three types of allatostatin precursors including A-type (Ast-A), B-type (Ast-B), and C-type (Ast-C) were identified, each exhibiting unique structural features and conserved motifs (Fig 1). The precursor of Ast-A was predicted to be 212 amino acids (aa) in length and encode eight mature peptides. These peptides are separated by dibasic cleavage sites and terminate with a glycine residue, enabling C-terminal amidation. Most mature peptides share the conserved C-terminal motif XYXFGL amide, where X represents variable residues. Despite this conserved motif, each predicted peptide differs in sequence and length, reflecting their structural variability. For Ast-B, the precursor was found to comprise 174 aa, starting with a 21-aa signal peptide (Fig 1). It encodes seven mature peptides, ranging from 9 to 11 aa in length, which are separated by dibasic cleavage sites. These peptides share a conserved XWXXXXGXW amide motif at their C-terminus, highlighting their functional consistency while maintaining some sequence variability. The Ast-C precursor was identified as a shorter sequence of 63 aa encoding a single mature peptide. This peptide is 14 aa long and features a conserved amidated C-terminal motif, -PISCF (Fig 1). The peptide structure is stabilized by a disulfide bridge formed between cysteine residues at positions 6 and 13. This structural arrangement is characteristic of C-type allatostatins [25]. The mature peptides encoded by allatostatin A, B, and C end with a glycine residue, which facilitates amidation at the C-terminus [26].

**Fig 2:**
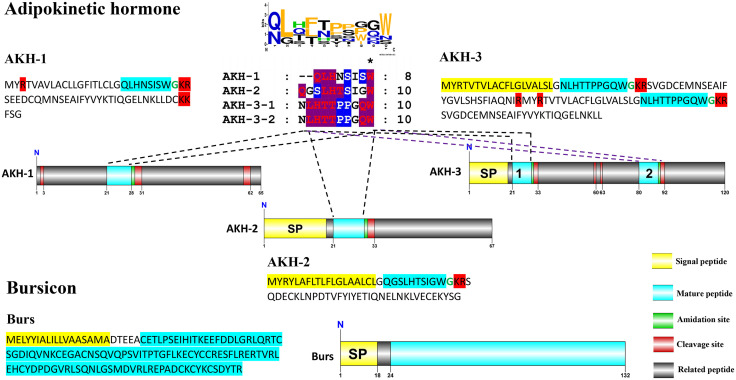
Identification and characterization of *E. integriceps*Adipokinetic hormone and Bursicon. AKH Schematics showing three AKH precursors identified in *E. integriceps*. Comparative sequence alignment of AKH mature peptides, sequence logos are shown above alignments. Burs Schematics showing Burs precursor identified in *E. integriceps*.

Transcriptome mining revealed three adipokinetic hormone (AKH) precursors (AKH-1, AKH-2, and AKH-3) and one bursicon (Burs) precursor, each displaying distinct structural characteristics and conserved motifs (Fig 2). The AKH precursors were predicted to encode 65, 67, and 120 (aa) for AKH-1, AKH-2, and AKH-3, respectively. AKH-1 contains a single predicted peptide that is separated by a dibasic cleavage site, consistent with neuropeptide maturation (Fig 2). Both AKH-2 and AKH-3 feature a signal peptide, 19 aa long, respectively, followed by mature peptide regions. The mature peptides encoded by AKH-2 and AKH-3 are 8–10 aa in length and terminate with a glycine residue, enabling amidation of the C-terminal (Fig 2). Notably, AKH-3 encodes two mature peptides, highlighting its structural complexity. These peptides display a conserved C-terminal motif, characteristic of AKH peptides (Fig 2). Burs is 132 aa long and begins with an 18 aa signal peptide (Fig 2). Unlike the AKH peptides, the mature peptide encoded by Burs lacks a glycine residue at the C-terminal, which precludes amidation. This precursor shares 89% sequence identity with the Burs precursor of the Oriental Stink Bug, *Plautia stali* (S2 Table), demonstrating evolutionary conservation.

**Fig 3:**
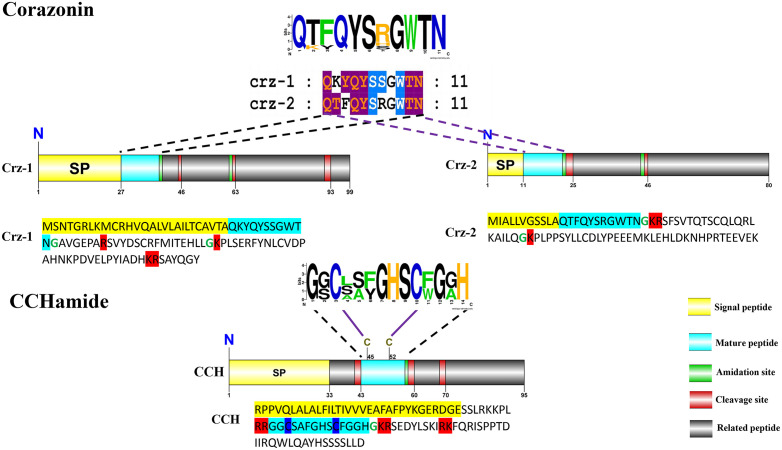
Identification and characterization of CCHamide and Corazonin in *E. integriceps.* Schematic showing CCH and two Crz precursors identified in *E. integriceps*. A sequence logo is shown above alignments, where the height of each letter is proportional to the observed frequency of the corresponding amino acid in the alignment column.

The predicted sequence of the CCHamide (CCH) precursor is composed of 95 aa with a predicted signal peptide of 22 aa (Fig 3). The mature peptide, a C-terminal amidated nonapeptide, starts at position 45 and ends at position 52, containing two cysteine residues. This conserved structure, GCXSFGHSCXGGHamide, highlights the functional conservation of the CCH mature peptide [27] (Fig 3). Two transcripts were identified to putatively encode a complete Corazonin precursor, consisting of 99 aa (Crz-1) and 80 aa (Crz-2), respectively (Fig 3). Crz-1 contains a predicted signal peptide of 27 aa, whereas Crz-2 has a 11 aa signal peptide. Both are followed by 11 predicted mature peptides, separated by dibasic cleavage sites. The mature peptides share a conserved sequence: QTFQYSRGWTN amide (Fig 3).

**Fig 4:**
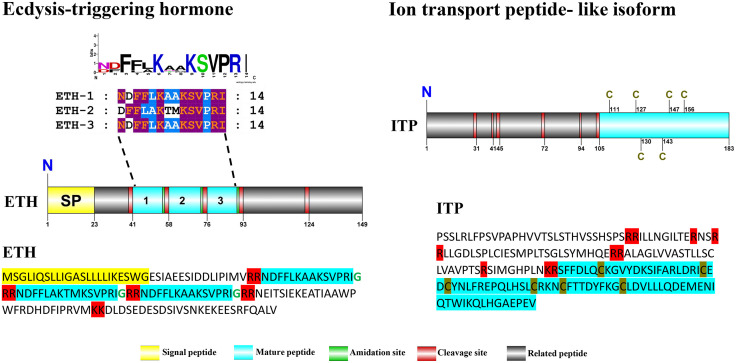
Identification and characterization of Ecdysis triggering hormone and Ion transport peptide in *E. integriceps.* Schematic showing ETH and ITP precursors and mature peptide identified in *E. integriceps*. A sequence logo is shown above alignments, where the height of each letter is proportional to the observed frequency of the corresponding amino acid in the alignment column.

The predicted ecdysis-triggering hormone precursor (ETH) consists of 149 aa, including a 23 aa signal peptide (Fig 4). This is followed by three predicted ETH peptides of 14 aa each, separated by dibasic cleavage sites (Fig 4). All three ETH peptides share high similarity with the conserved motif VPRLamide (Fig 4), which is characteristic of the ETH family possessing the C-terminal motif -PRXamide. This motif is widely conserved in invertebrates [28]. One transcript was identified that potentially encodes a complete ion transport peptide-like isoform (ITP) consisting of 183 aa, which contains a dibasic cleavage site and lacks the glycine necessary for amidation of the peptide. The mature ITP peptide, comprising 79 aa, includes 6 cysteine residues (Fig 4). This is consistent with the ITP from *Halyomorpha halys* (S2 Table), which also contains six cysteine residues in its mature peptide, with these cysteines being well-aligned. The ITP precursor showed a 99% identity with the ITP from the brown marmorated stink bug (*H. halys*).

**Fig 5:**
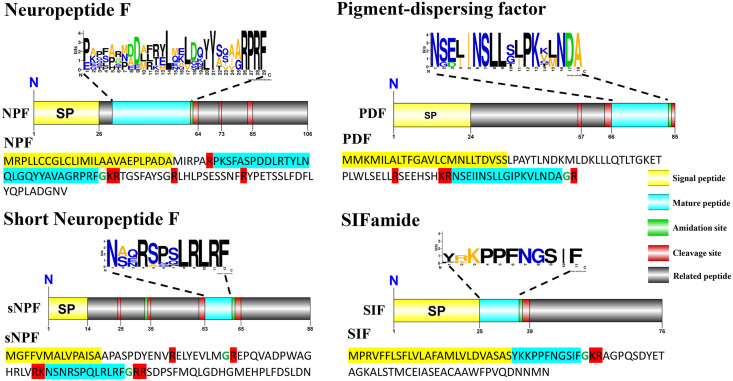
Identification and characterization of Neuropeptide F, Short Neuropeptide F, Pigment dispersing factor and SIFamide in *E. integriceps.* Schematic showing NPF, sNPF, PDF and SIF precursors and mature peptide identified in *E. integriceps*. A sequence logo is shown above alignments, where the height of each letter is proportional to the observed frequency of the corresponding amino acid in the alignment column.

The nucleotide sequence produced from the transcriptome data encodes a complete neuropeptide F precursor (NPF) with 104 aa, beginning with a 26 aa signal peptide (Fig 5). The predicted 29-aa mature peptide contains the conserved RPRFamide motif and shares the highest BLAST similarity with the neuropeptide F of *Rhodnius prolixus* (S2 Table). One transcript was identified that encodes a complete short Neuropeptide F precursor (sNPF) consisting of 89 aa, starting with a 34 aa signal peptide. This is followed by a single predicted peptide, 12 aa in length, containing the conserved XPXRLRFamide motif (Fig 5). The LOGO analysis of NPF and sNPF confirms their conserved C-terminal motif: RXRFamide (Fig 5). The search for Pigment-dispersing factor precursors (PDF) revealed a transcript encoding a complete 85 aa precursor. This precursor starts with a 24 aa signal peptide and contains a mature peptide of 18 aa, separated by dibasic cleavage sites (Fig 5). The PDF precursor shares over 80% identity with the PDF of the Southern green stink bug, *Nezara viridula* (S2 Table). In the transcriptome assembly, a single transcript encodes a complete SIF amide precursor (SIF) comprising 76 aa, beginning with a 25 aa signal peptide. The predicted mature peptide, GYRKPPFNGSIFamide, is located immediately after the signal peptide (Fig 5). The SIF precursor shows high homology (93% identity) with the SIF of the Oriental Stink Bug, *P. stali* (S2 Table). Additionally, the mature SIF peptide displays a conserved structure: XYRKPPFNGSIFamide.

### Neuropeptide expression profile

#### Expression profile of neuropeptide during development.

**Fig 6:**
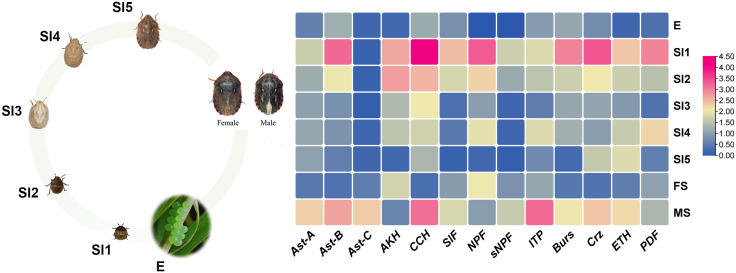
Expression patterns of 13 neuropeptide genes across developmental stages and between sexes in *E. integriceps.*

The gene expression in *E. integriceps* revealed dynamic fluctuations across developmental stages (Fig 6), with the full statistical analyses supporting these differences presented in S1 Fig. The Highest expression levels were predominantly found in early instars (Sunn pest Instar (SI) 1-SI2), particularly for *Ast-A*, *Ast-B, CCH, AKH, Crz*, and *sNPF*. Later stages (SI3-SI5) generally exhibited lower expression, except for genes like *PDF* and *ITP*, which showed an initial decline in SI3 followed by an increase in SI4. *Ast-C* and *AKH* showed unique expression peaks in SI2 indicating stage-specific regulatory roles. Although various types of allatostatins (*Ast-A, Ast-B, Ast-C*), are expressed in all developmental stages of *E. integriceps* (S1 Fig), there are differences in their expression levels across stages. *Ast-A* and *Ast-B* exhibited higher expression during the 1st instar nymph stage compared to later stages, while *Ast-C* was predominantly expressed in eggs, showing a 3.2-fold increase compared to all other stages (S1 Fig). *CCH* and *AKH*, *CCH* was highly expressed in SI1 (2.9-fold higher than in SI5). *AKH* peaked in SI2, with expression levels nearly 3.1-fold higher than in eggs. *PDF* and *ITP* showed a similar trend. Expression levels decreased significantly in SI3 (1.8-fold lower than in SI1) and increased again in SI4 (2.4-fold higher compared to SI3). *Burs* exhibited the highest expression in SI2, with a 2.6-fold increase compared to S5. *ETH,* which is crucial for initiating molting, showed the highest level in SI1 (3.0-fold increase compared to eggs) and remained relatively stable across later stages [29]. (*sNPF* and *NPF*), *sNPF* and *NPF* had peak expression in SI1, with *sNPF* being 3.5-fold higher and *NPF* 2.9-fold higher than in SI5. Among the two evaluated reference genes, *18S rRNA* showed the most stable expression across all developmental stages. The detailed stability metrics (mean, SD, SEM, and CV%) and the corresponding statistical analyses are provided in S2 Fig and Table S4.

This figure illustrates the relative expression levels of 13 neuropeptide genes at different developmental stages of *E.integriceps*, from egg to the 5th instar nymph, as well as in adult males and females. The heatmaps represent gene expression profiles, with red indicating upregulation and blue indicating downregulation. The developmental stages include egg, 1st to 5th instar nymphs (SI1–SI5), and adult males and females. The expression levels of these genes were analyzed to understand their potential role in growth, development, and sex-specific physiological processes. The color scale at the bottom right shows expression intensity values.

#### The expression profile of adult females and males.

The expression profiles of 13 neuropeptides in adult *E. integriceps* were analyzed to identify sex-specific differences (Fig 6 and S2 Fig). The results revealed significant variation in the expression levels of multiple neuropeptides between males and females of certain neuropeptides being preferentially expressed in one sex. Overall, the expression levels of *Ast-A*, *Ast-B*, *Ast-C*, *AKH*, *SIF*, *ITP*, *Burs*, *sNPF*, *NPF*, and *Crz* were significantly higher in males compared to females. In contrast, *CCH* and *PDF* were expressed at higher levels in females. No significant difference was found in *ETH* expression between the two sexes.

#### Organ-specific expression profile.

**Fig 7:**
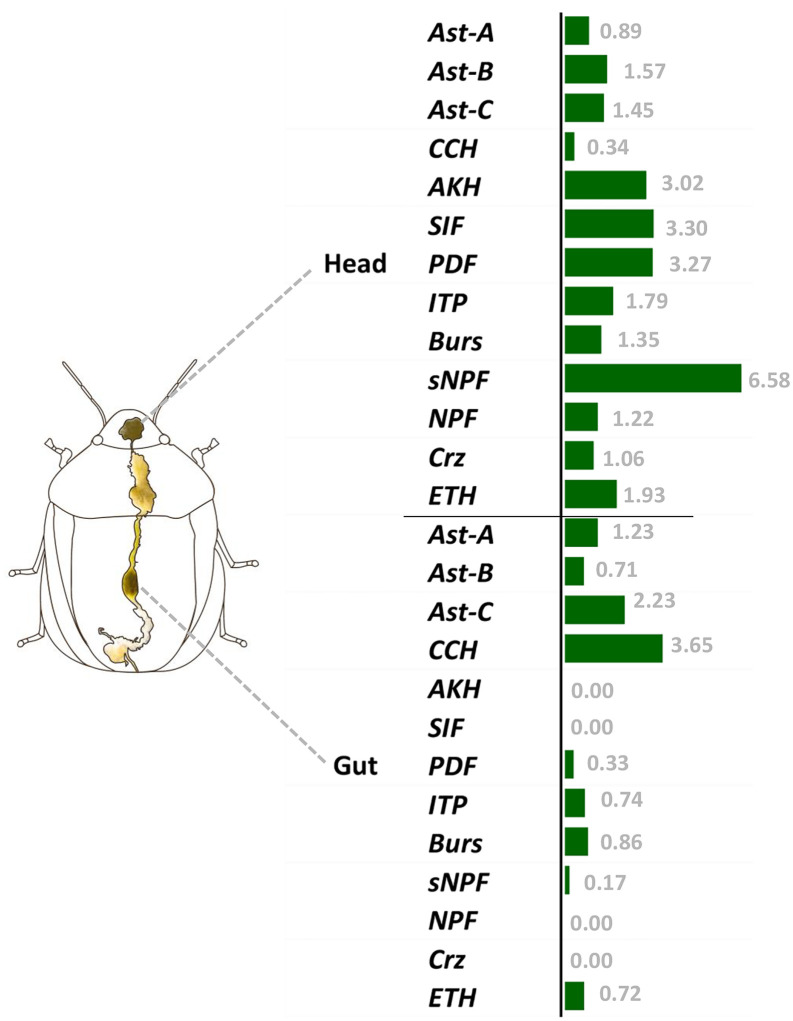
Comparative expression of 13 neuropeptide genes in the head and gut of adult *E. integriceps.*

The organ-specific expression profiles of 13 neuropeptide precursors in *E. integriceps* adults were examined in the head and gut (Fig 7), and the statistical analyses supporting these patterns are presented in S3 Fig. The results showed that all neuropeptides were expressed in the head. In contrast certain neuropeptides associated with central nervous system, including *SIF*, *NPF*, *Crz*, and *AKH*, were not detected in the gut. The neuropeptides that were detected in both organs comprising of *Ast-A*, *Ast-C*, and *CCH* exhibited significantly higher expression in the gut compared to the head. Conversely, *Ast-B*, *PDF*, *ITP*, *sNPF*, and *ETH* were predominantly expressed in the head (Fig 7). Interestingly, *Burs* did not show any significant difference in expression between the head and gut, implying that its function may be distributed across both organs or may be regulated post-transcriptionally rather than at the expression level.

This figure illustrates the comparative expression levels of 13 neuropeptide genes in the head and gut of adult *E. integriceps*. The anatomical diagram highlights the head and gut, with bar graphs representing gene expression levels in each organ. Bars positioned at the top represent the head, while those at the bottom represent the gut. The variation in expression levels suggests potential organ-specific functions of these neuropeptides in physiological and regulatory processes.

## Discussion

Neuropeptides and peptide hormones are essential for cell-to-cell communication in multicellular animals [30,31]. They are derived from precursor proteins that undergo posttranslational modifications such as amidation that make them biologically active [32,33]. Neuropeptides and their receptors are highly conserved, playing critical roles in reproduction, metabolism, growth, and locomotion. [30,34]. Due to their physiological significance, neuropeptides have been suggested as potential insecticidal targets [35]. The expression of neuropeptides in the *E. integriceps* is, however, poorly studied. In this study, the structure and expression patterns of identified neuropeptides involved in feeding regulation are examined across developmental stages, organs (head and gut), and adult males and females of *E. integriceps*.

### Neuropeptides identification and structures

Allatostatin peptides play a crucial role in juvenile hormone regulation [36] and are diverse peptides derived from three distinct genes in insects, designated as *Ast-A, Ast-B, and Ast-C* [37]. In this study, the structural characterization of Ast-A, Ast-B, Ast-C peptides revealed both conserved motifs and sequence variability. There are eight mature peptides encoded by the Ast-A precursor, all of which share the characteristic C-terminal motif XYXFGL amide, which represents a hallmark of this family. However, the variable residues (X) in the motif and peptide lengths may be able to fine-tune the interaction with receptor subtypes, facilitating functional plasticity across tissues or developmental stages. Similarly, Ast-B peptides in Sunn pest exhibit a conserved XWXXXXGXW amide motif, which aligns with their role in regulating juvenile hormone synthesis like other insects. The shorter and more uniform length of Ast-B peptides (9–11 aa) compared to Ast-A (212 aa) may reflect distinct release dynamics or receptor-binding kinetics.

AKH, a key neuropeptide, regulates lipid and carbohydrate levels, energy metabolism, and the female reproductive system in insects [38]. In *E. integriceps*, we identified three neuropeptide precursors, two of which possess signal peptides (Fig 2). Prior studies have demonstrated that injecting mature AKH peptide elevates carbohydrate and lipid levels in *Bombyx mori* hemolymph [39]. Similarly, in *Coccinella septempunctata*, CsAKH and CsAKHR control lipid storage and ovarian development during diapause [40]. The presence of two signal peptides in AKH precursors suggests that these peptides are secreted and act as endocrine or paracrine signals, aligning with AKH’s established mode of action, where it is released from the corpora cardiaca into the hemolymph to exert systemic effects [41,42].

We also identified the CCH precursor, which contains a signal peptide and a conserved GCXSFGHSCXGGH structure (Fig 3), aligning well with CCH found in other insects. For instance, in *Drosophila melanogaster*, CCHamide-1 and CCHamide-2 are critical for stimulating food intake [43], whereas in *Gryllus bimaculatus*, CCH modulates feeding behavior and nutrient signaling [44]. Furthermore, CCH influences feeding motivation in blowflies and enhances sensory perception and olfactory responses in starved *D. melanogaster* [45]. Therefore, the identified CCH precursor in *E. integriceps* likely plays similar regulatory roles, potentially impacting feeding behavior and reproduction in this species.

ETH, as the identified neuropeptide precursor in *E. integriceps*, and its three mature ETH peptides, showed considerable similarity to the conserved VPRL amide motif (Fig 4). The process of ecdysis in insects requires ETH, an essential endocrine signal [46]. Besides its function in ecdysis, ETH is also associated with reproductive regulation [47]. Prior study showed ETH influences on juvenile hormone-related courtship memory and reproductive viability in several insect species, thus showing its multifaceted properties [48].

An additional significant finding was the identification of the ITP precursor. This precursor contains six spatially conserved cysteines in the mature peptide [45]. These cysteines form three intramolecular disulfide bonds, which stabilize the hormone (Fig 4). ITP is secreted by the corpora cardiaca and induces the ileum to transfer Cl^-1^ ions from the lumen to the hemolymph, establishing an electrochemical gradient that facilitates water reabsorption [49]. Two neuropeptide F precursors were identified in this research; the mature peptides are 29 and 12 amino acids, respectively, and are referred to as NPF and sNPF (Fig 5). The C-terminal end of both peptides includes the conserved RXRF motif. The neuropeptide NPF in insects, containing 7–40 amino acids and a conserved RPRF amide motif, is critical for its functional activity in feeding, metabolism, reproduction, and stress responses [38,50]. In *Drosophila*, NPF regulates food intake and body size by modulating insulin-like peptides [51].

### Developmental expression patterns of neuropeptides

The dynamic expression profiles of neuropeptides throughout the developmental stages of *E. integriceps* provide essential information regarding their functional roles in growth, the regulation of feeding, and molting. The highest levels of neuropeptide expression, notably *Ast-A*, *Ast-B*, *CCH*, *AKH*, *Crz*, and *sNPF*, were detected in early instars (SI1-SI2). This shows their participation in the initial stages of development and feeding. Conversely, the expression of most neuropeptides in later stages (SI3-SI5), excluding *PDF* and *ITP*, showed a biphasic pattern, with an initial decline in SI3 followed by an increase in SI4. For Neuropeptide *PDF*, expression decreased to 0.3-fold in SI3 and subsequently increased to 3.7-fold in SI4. Similarly, Neuropeptide *ITP* showed a reduction to 0.4-fold in SI3 followed by an increase to 2.5-fold in SI4. These patterns suggest that neuropeptides may have stage-specific regulatory functions in *E. integriceps*. For example, Allatostatins *Ast*-A and *Ast*-B peaked during the first instar, with 2.1-fold and 8.1-fold increases relative to eggs, respectively, whereas *Ast*-C exhibited its highest expression in eggs. During nymphal stages, *Ast*-C expression was lower than in eggs, with fold-changes of 0.5, 0.3, 0.2, 0.4, and 0.2 in the first to fifth instars, respectively (S1 Fig), suggesting its role in early embryonic development rather than feeding regulation. The results are in agreement with earlier studies showing that the gene coding for allatostatins controls juvenile hormone metabolism, which affects development and feeding behavior [52]. Earlier findings revealed that Lepidopteran Ast-C feeding resulted in reduced growth and fecundity and induced considerable mortality in *Acyrthosiphon pisum* and *Myzus persicae* [53,54]. Furthermore, Cao et al. found that allatostatin compounds specifically inhibit the synthesis and secretion of a specific juvenile hormone in *Spodoptera frugiperda*, which affects the ecdysis and growth of insects and leads to mortality [52,55]. *CCH* and *AKH*, which are recognized for their functions in metabolic regulation, exhibited elevated expression in early instars. Elevated *CCH* expression in SI1 relative to SI5 indicates a potential role for *CCH* in early nutritional signaling. *AKH* showed elevated expression in SI2; its expression was significantly higher than in eggs, which suggests *AKH* may be involved in lipid mobilization and energy metabolism, as previously reported [5]. The cerebral expression profiles of *CCH* at the early vitellogenic stage suggested a possible role in promoting vitellogenesis, as previously indicated in *Scylla paramamosain* [56]. Distinctive expression patterns were observed in the *PDF*, *ITP*, *Burs*, and *ETH* genes that regulate molting. *PDF* and *ITP* exhibited a biphasic pattern, declining in SI3 but increasing in SI4, potentially indicating their involvement in circadian rhythm regulation and physiological adaptation during molting [5,57]. *Burs*, which is key to hardening the cuticle post-molting, had a high level of expression in SI2 versus SI5, while *ETH*, which is required for starting molting, showed its maximum expression in SI1 in contrast to eggs. The results indicate that *ETH* is involved in early molting stages, while *PDF* and *ITP* govern the later molting transitions [29]. The *sNPF* and *NPF* neuropeptide F family members demonstrated peak expression in SI1, and *sNPF* and *NPF* levels were significantly greater than those observed in SI5. In later instars, the expression of these genes was significantly reduced, indicating that *NPFs* are essential for controlling feeding behavior primarily in the early stages of development, a pattern consistent with observations in other insect species [12,58]. The initial high expression of *Ast-A*, *Ast-B*, *CCH*, and *sNPF* strengthens the hypothesis that they have a role in stimulating feeding behavior and promoting growth in young instars. In contrast, the delayed *PDF* and *ITP* peaks in SI4 could suggest their involvement in circadian and physiological adaptations prior to the ultimate molting stage [5,29].

### Sex-specific expression of neuropeptides

Sexual dimorphisms in neuropeptide expression were observed; specifically, *Ast-A*, *Ast-B*, *Ast-C*, *AKH*, *SIF*, *ITP*, *Burs*, *sNPF*, *NPF*, and *Crz* displayed elevated expression in males, while *CCH* and *PDF* were expressed primarily in females. The absence of significant variation in *ETH* expression suggests the conservation of its function across sexes. These findings imply that neuropeptides contribute to sexually dimorphic physiological functions, potentially influencing reproductive processes, metabolism, and behavioral adaptations. A significant difference in the expression of *NPF*, identified in *E. integriceps*, was observed between adult males and females (Fig 6, S2 Fig). Nevertheless, these neuropeptides are essential in the regulation of numerous physiological functions, such as development, reproduction, and feeding [37]. The higher expression of *sNPF* and *NPF* in males may correlate with increased feeding activity and energy metabolism, while heightened *PDF* expression in females might be indicative of a role in circadian rhythm regulation [37]. In a comparable study by Yao et al., *PDF* expression was predominantly detected in female adults of *S. frugiperda* and is essential for the circadian rhythm of Drosophila and other insects [59]. In our study, *SIF* exhibited higher expression in males (Fig 6). *SIF* are neuropeptide precursors that are highly conserved and involved in regulating sexual behavior, feeding, and sleep [60]. *SIF* knockdown in *R. prolixus* decreased blood meal consumption; however, injection of *Rhopr-SIF* increased blood meal intake [61]. The widespread distribution of *SIF* in the CNS, along with numerous varicosities throughout the ganglia, may indicate its potential involvement in regulating adult functions, such as reproduction [60]. Regarding sexual differences, the release of *SIF* in the brain may inhibit sexual behavior until the flies encounter the appropriate physiological conditions, suggesting a role in sexual dimorphism [62]. Nevertheless, these inferences necessitate additional validation through tissue localization studies, functional inhibition assays, and other experimental methodologies.

### Organ-specific expression of neuropeptides

The organ-specific expression profiles of neuropeptides in *E. integriceps* provide essential data for in-depth investigation of the biology and ecology of this vital biological control species. Most of the *E. integriceps* neuropeptide precursor genes were expressed in the brain (Fig 7, S3 Fig). The data suggests that *Ast-B*, *PDF*, *ITP*, and *ETH* expression was predominant in the brain, implying that most neuropeptide signaling systems act as neuroregulators in predatory bugs [63]. Conversely, *Ast-C* and *CCH* exhibited broad expression within the gut (Fig 7). In *Arma custos*, the gut exhibited high expression levels of *CCH* and *Ast-C* [63]. *Ast-C* and *CCH* are brain-gut peptides that are conserved and that have functions such as myotropic effects and feeding behavior regulation [64]. Drosophila CCHamide‑2 is expressed in the gut and fat body, promoting larval feeding and controlling body growth [65]. In insects, neuropeptides within the gut control feeding behavior, which includes the selection of food, appetite regulation, digestion, metabolism, and excretion [60]. Notably, *SIF*, *AKH*, *NPF*, and *Crz* expression was confined to the brain (Fig 7), which aligns with observations in other insect species [45,66,67]. SIFamide-like immunoreactivity has been localized in the CNS of *R. prolixus* [68]. *SIF* is produced exclusively by four interneurons that project throughout the brain [67]. *AKH* is expressed in endocrine cells within the two neurohemal organs, corpora cardiaca, frequently fused and located near the insect brain.

## Conclusions

This study provides a detailed functional characterization of 13 feeding-related neuropeptides in Sunn pest (*E. integriceps*), a major wheat pest in Iran. Using the data mining of our previously generated whole-body RNA-seq dataset, we conducted structural analyses and expression profiling, revealing unique temporal, sex-specific, and tissue-specific expression patterns for each neuropeptide. The increased expression of *Ast-A*, *Ast-B*, *CCH*, *AKH*, *Crz*, and *sNPF* in early instars supports their significant contribution to nymphal feeding and growth, while the biphasic expression pattern of *PDF* and *ITP* suggests these neuropeptides involvement in molting and circadian regulation. It is noteworthy that the expression of most neuropeptides differs between the sexes, with males expressing more than females (*CCH* and *PDF*), which suggests specialized roles in reproduction and metabolism. The primary brain localization of most neuropeptides supports their neuro-regulatory roles, though the gut-specific expression of *Ast-C* and *CCH* suggests further roles in digestive physiology. According to these findings, neuropeptide signaling can potentially be used to control pests specifically adapted to a species through a process known as neuropeptide signaling. In order to develop sustainable alternatives to conventional insecticides within integrated pest management strategies, future research must focus on functionally characterizing these neuropeptides and their receptor binding in order to confirm their involvement in feeding and development.

## Supporting information

S1 Table List of primers used for gene expression analysis. (DOCX)

S2 Table Identified neuropeptide precursors in *E. integriceps.* (DOCX)

S3 Table Neuropeptide Genes of *E. integriceps*: mRNA and Predicted Protein Sequences. (XLSX)

S4 Table Stability of 18S reference gene across developmental stages of *E. integriceps.* (XLSX)

S1 Fig RT-qPCR results of neuropeptides throughout the *E. integriceps* life cycle. The y-axis represents the relative expression level and the x-axis the life cycle. The standard error is represented by the error bar and significant differences are represented by the different letters (p < 0.05). E: egg, SI (Sunn pest Inestar)1: 1th-instar nymphs, SI2: 2th-instar nymphs; SI3: 3th instar nymphs; SI4: 4th instar nymphs; SI5: 5th instar nymphs. *Ast-A*: allatostatin-A; *Ast-B*: allatostatin-B; *Ast-C*: allatostatin-C; *CCH*: CCHamide; *AKH*: Adipokinetic hormone; *SIF*: SIFamide; *PDF*: Pigment-dispersing factor; *ITP*: Ion transport peptide; *Burs*: Bursicon; *sNPF*: Short NPF; *NPF*: Neuropeptide F; *Crz*: Corazonin; *ETH*: Ecdysis triggering hormone. Lowercase letters above the error bars indicate that significant differences among different stages or various tissues (P < 0.05, one-way ANOVA followed by Tukey’s test).
(PNG)

S2 Fig Relative expression of neuropeptides in both sexes of *E. integriceps.* FS: Female Sunn pest, MS: Male Sunn pest. *Ast-A*: allatostatin-A; *Ast-B*: allatostatin-B; *Ast-C*: allatostatin-C; *CCH*: CCHamide; *AKH*: Adipokinetic hormone; *SIF*: SIFamide; *PDF*: Pigment-dispersing factor; *ITP*: Ion transport peptide; *Burs*: Bursicon; *sNPF*: Short NPF; *NPF*: Neuropeptide F; *Crz*: Corazonin; *ETH*: Ecdysis triggering hormone. Data are expressed as mean ± standard error (SE). Differences between groups were assessed using one-way ANOVA followed by a t-test. Asterisks denote statistically significant differences: *p < 0.05; **p < 0.01; ***p < 0.001.
(PNG)

S3 Fig The organ-specific transcript abundances of *E. integriceps* neuropeptides. *Ast-A*: allatostatin-A; *Ast-B*: allatostatin-B; *Ast-C*: allatostatin-C; *CCH*: CCHamide; *AKH*: Adipokinetic hormone; *SIF*: SIFamide; *PDF*: Pigment-dispersing factor; *ITP*: Ion transport peptide; *Burs*: Bursicon; *sNPF*: Short NPF; *NPF*: Neuropeptide F; *Crz*: Corazonin; *ETH*: Ecdysis triggering hormone. Data are expressed as mean ± standard error (SE). Differences between groups were assessed using one-way ANOVA followed by a t-test. Asterisks denote statistically significant differences: *p < 0.05; **p < 0.01; ***p < 0.001.
(PNG)
