# Supplementary material for: Exploration of neuropeptides to identify potential target for regulating feeding behavior and development in Eurygaster integriceps
Source: PLoS One. 2026 Jul 17;21(7):e0353952. doi: 10.1371/journal.pone.0353952 (PMC13379102; doi:10.1371/journal.pone.0353952)
Supplement: S1 Table — (DOCX) [file pone.0353952.s001.docx]

**Table S 1**. List of primers used for gene expression analysis.

| **Primer Name** | **Forward primer (5′→3′)** | **Reverse primer (5′→3′)** | **Target Gene** | **Annealing Temperature (°C)** |
| --- | --- | --- | --- | --- |
| Ast -A | TTGGGTTAGCCTCATCTT | CAGGTTTTCATTTGGGCTTG | Allatostatin A | 51 |
| Ast -B | CCAGGCCTAAACAACCTTGAAG | GGCTCAGCGATACAACCATG | Allatostatin B | 58 |
| Ast -C | GGTCGCTAACAACCCTCTA | TGCTTGGCGAACAGGTAG | Allatostatin C | 56 |
| SIF | TCCTCGTTCTCGCCTTCGCC | ATCAGATTGTGGTCCAGCCCTC | SIFamide | 58 |
| NPF | TCCTAGATTTGGGAAACGC | TTCCATCTGCCAGTGGTTG | Neuropeptide F (long Neuropeptide F ) | 58 |
| ETH | GTCAGTGCCTCGAATAGGACG | CATGATCCCTGAACCATGGC | Ecdysis-triggering hormone | 56 |
| sNPF | ATGGAGAGATAAAAGGAGAAGG | ACACCCTCTCTTTGATTCTC | Neuropeptide F (short Neuropeptide F) | 51 |
| AKH | TGGATGGTTTTGTAGACGTAGA | CTTCTTGGGCTTGGTTGC | Adipokinetic hormone | 54 |
| Crz | AGCTGCAGGTTCATGATCAC | GGAGTTCGACGTCAGGTTTG | Corazonin | 55 |
| Burs | ACATCGCTCTCATACTGATAG | GCACTTGTTCACCTGGATGT | Bursicon | 53 |
| PDF | TAGCGACCTGCGTCATTTAG | CAAGCTACTGTTGCAGACCC | Pigment-dispersing factor | 55 |
| CCH | TTGTAGCCATTGCCGAAT | TTGGTCACTCCTGCTTCG | CCHamide | 53 |
| 18S | GGCCTTCGGGATCGGAGTAA | GCAAATGCTTTCGCAGTTGTT | 18S rRNA | 58 |
