# Supplementary material for: Exploration of neuropeptides to identify potential target for regulating feeding behavior and development in Eurygaster integriceps
Source: PLoS One. 2026 Jul 17;21(7):e0353952. doi: 10.1371/journal.pone.0353952 (PMC13379102; doi:10.1371/journal.pone.0353952)
Supplement: S2 Table — (DOCX) [file pone.0353952.s002.docx]

**Table S 2**. Identified neuropeptide precursors in Eurygaster integriceps

|  | | | | | Homology search with known protein | | | |
| --- | --- | --- | --- | --- | --- | --- | --- | --- |
| Neuropeptide | Unigene ID | Abbreviation | ORF (aa) | SP (aa) | Species | Protein ID | E-Value | Identity (%) |
| Adipokinetic hormone | TRINITY_DN1013_c0_g1_i2 | AKH-1 | 65 | ̴ | *Plautia stali* | BAV78787.1 | 6.00E-19 | 50 |
| Adipokinetic hormone | TRINITY_DN17312_c0_g1_i1 | AKH-2 | 67 | 19 | *Plautia stali* | BAV78787.1 | 1.00E-21 | 61 |
| Adipokinetic hormone | TRINITY_DN7407_c0_g1_i1 | AKH-3 | 120 | 19 | *Nezara viridula* | AZK31332.1 | 2.00E-12 | 48 |
| Allatostatin A | TRINITY_DN53332_c0_g1_i1 | Ast-A | 212 | ̴ | *Plautia stali* | BAV78789.1 | 4.00E-103 | 84 |
| Allatostatin B | TRINITY_DN1251_c1_g1_i1 | Ast-B | 174 | 21 | *Plautia stali* | BAU88428.1 | 6.00E-64 | 81 |
| Allatostatin C | TRINITY_DN208820_c0_g1_i1 | Ast-C | 63 | ̴ | *Plautia stali* | BAV78790.1 | 6.00E-16 | 94 |
| Bursicon | TRINITY_DN101716_c0_g1_i1 | Burs | 132 | 18 | *Plautia stali* | BAV78794.1 | 2.00E-84 | 89 |
| CCHamide | TRINITY_DN6297_c0_g1_i1 | CCH | 95 | 32 | *Plautia stali* | BAV78798.1 | 7.00E-46 | 80 |
| Corazonin | TRINITY_DN26128_c0_g1_i2 | Crz-1 | 99 | 27 | *Halyomorpha halys* | XP_014274138.1 | 3.00E-05 | 38 |
| Corazonin | TRINITY_DN27226_c0_g1_i1 | Crz-2 | 80 | 11 | *Plautia stali* | BAV78800.1 | 1.00E-38 | 67 |
| Ion transport peptide-like isoform | TRINITY_DN8446_c0_g1_i1 | ITP | 183 | ̴ | *Halyomorpha halys* | XP_014274474.1 | 1.00E-89 | 99 |
| Ecdysis-triggering hormone | TRINITY_DN116259_c0_g1_i1 | ETH | 149 | 23 | *Plautia stali* | BAV78804.1 | 5.00E-68 | 71 |
| Neuropeptide F (long transcript) | TRINITY_DN26518_c0_g1_i1 | NPF | 106 | 26 | *Rhodnius prolixus* | AMW92660.1 | 2.00E-31 | 63 |
| Neuropeptide F (short transcript) | TRINITY_DN134045_c0_g1_i1 | sNPF | 89 | 14 | *Halyomorpha halys* | XP_014284284.1 | 1.00E-57 | 89 |
| Pigment-dispersing factor | TRINITY_DN73472_c0_g1_i1 | PDF | 85 | 24 | *Nezara viridula* | AZK31364.1 | 2.00E-45 | 81 |
| SIF amide | TRINITY_DN28987_c0_g1_i1 | SIF | 76 | 25 | *Plautia stali* | BAV78828.1 | 7.00E-45 | 89 |
| ORF, open reading frame; SP, signal peptide; ̴ no signal peptide | | | | | | | | |
