# Supplementary figures and images for: Exploration of neuropeptides to identify potential target for regulating feeding behavior and development in Eurygaster integriceps
Source: PLoS One. 2026 Jul 17;21(7):e0353952. doi: 10.1371/journal.pone.0353952 (PMC13379102; doi:10.1371/journal.pone.0353952)

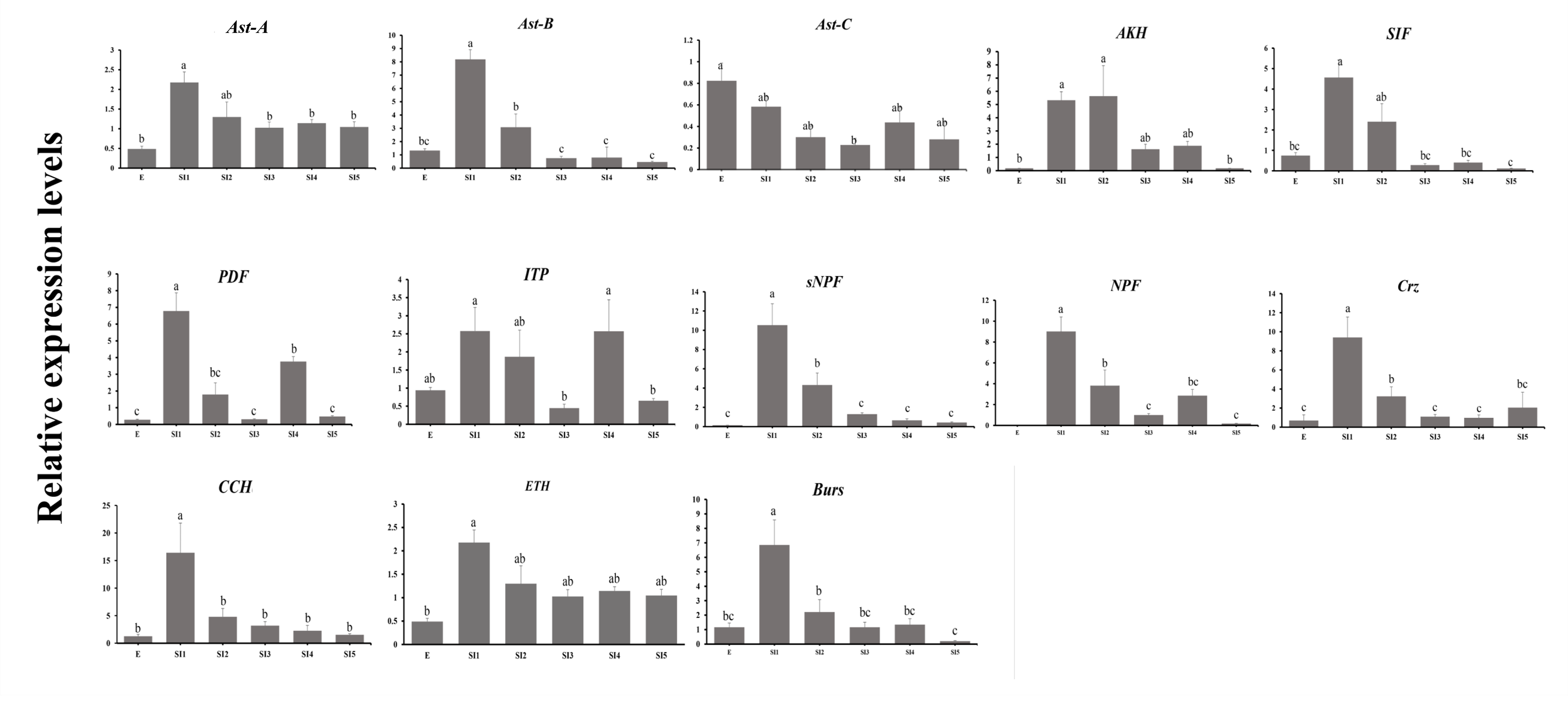

Supplement: S1 Fig — The y-axis represents the relative expression level and the x-axis the life cycle. The standard error is represented by the error bar and significant differences are represented by the different letters (p < 0.05). E: egg, SI (Sunn pest Inestar)1: 1th-instar nymphs, SI2: 2th-instar nymphs; SI3: 3th instar nymphs; SI4: 4th instar nymphs; SI5: 5th instar nymphs. Ast-A: allatostatin-A; Ast-B: allatostatin-B; Ast-C: allatostatin-C; CCH: CCHamide; AKH: Adipokinetic hormone; SIF: SIFamide; PDF: Pigment-dispersing factor; ITP: Ion transport peptide; Burs: Bursicon; sNPF: Short NPF; NPF: Neuropeptide F; Crz: Corazonin; ETH: Ecdysis triggering hormone. Lowercase letters above the error bars indicate that significant differences among different stages or various tissues (P < 0.05, one-way ANOVA followed by Tukey’s test). (PNG) [file pone.0353952.s005.png]

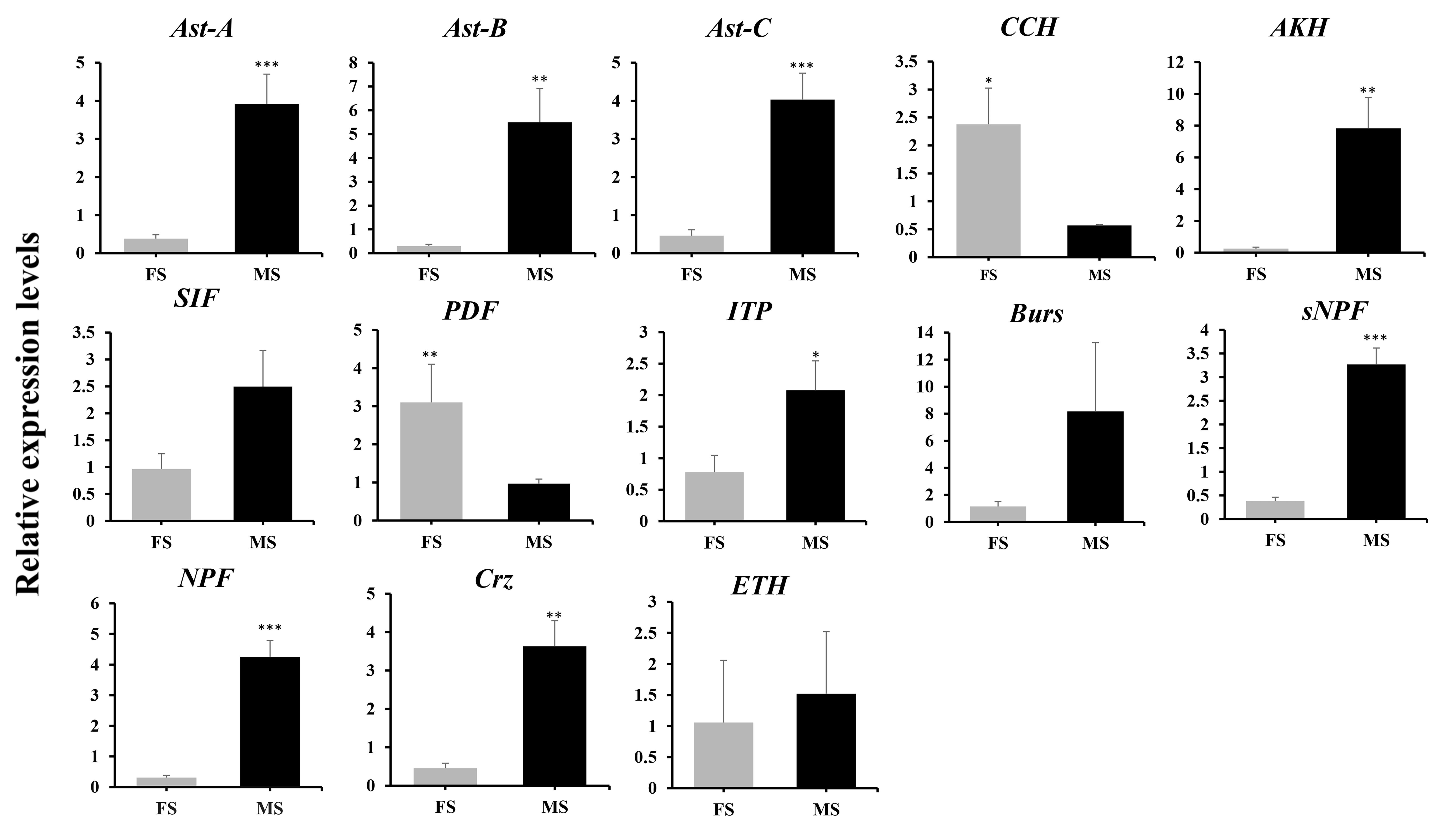

Supplement: S2 Fig — FS: Female Sunn pest, MS: Male Sunn pest. Ast-A: allatostatin-A; Ast-B: allatostatin-B; Ast-C: allatostatin-C; CCH: CCHamide; AKH: Adipokinetic hormone; SIF: SIFamide; PDF: Pigment-dispersing factor; ITP: Ion transport peptide; Burs: Bursicon; sNPF: Short NPF; NPF: Neuropeptide F; Crz: Corazonin; ETH: Ecdysis triggering hormone. Data are expressed as mean ± standard error (SE). Differences between groups were assessed using one-way ANOVA followed by a t-test. Asterisks denote statistically significant differences: *p < 0.05; **p < 0.01; ***p < 0.001. (PNG) [file pone.0353952.s006.png]

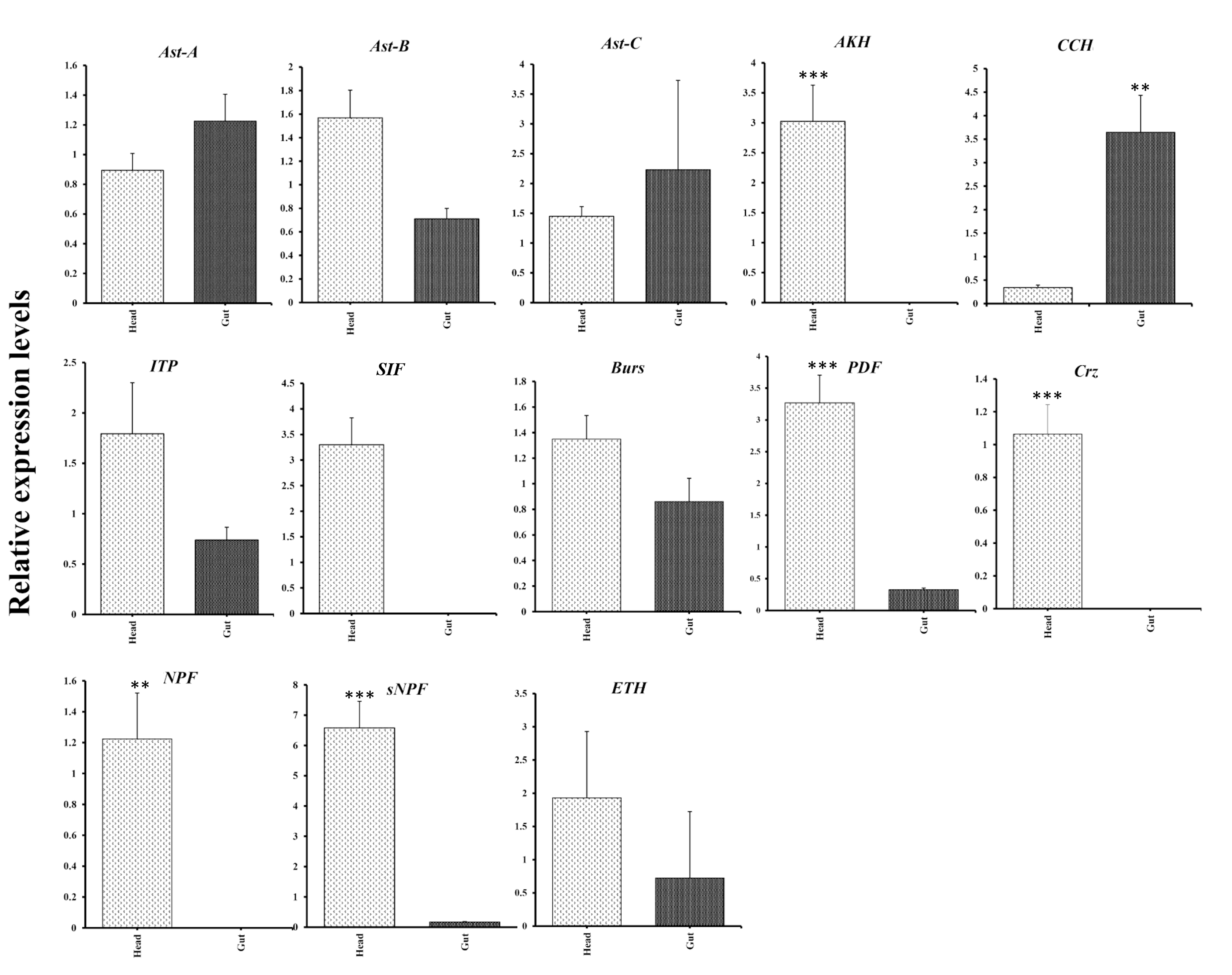

Supplement: S3 Fig — Ast-A: allatostatin-A; Ast-B: allatostatin-B; Ast-C: allatostatin-C; CCH: CCHamide; AKH: Adipokinetic hormone; SIF: SIFamide; PDF: Pigment-dispersing factor; ITP: Ion transport peptide; Burs: Bursicon; sNPF: Short NPF; NPF: Neuropeptide F; Crz: Corazonin; ETH: Ecdysis triggering hormone. Data are expressed as mean ± standard error (SE). Differences between groups were assessed using one-way ANOVA followed by a t-test. Asterisks denote statistically significant differences: *p < 0.05; **p < 0.01; ***p < 0.001. (PNG) [file pone.0353952.s007.png]
