# Supplementary material for: Exploration of neuropeptides to identify potential target for regulating feeding behavior and development in Eurygaster integriceps
Source: PLoS One. 2026 Jul 17;21(7):e0353952. doi: 10.1371/journal.pone.0353952 (PMC13379102; doi:10.1371/journal.pone.0353952)
Supplement: Attachment — Submitted filename: PONE-D-25-46793_reviewer.pdf [file pone.0353952.s008.pdf]

PLOS ONE

Exploration of neuropeptides to identify potential target for regulating feeding behavior and development in Eurygaster integriceps

--Manuscript Draft--

**Manuscript Number:**	PONE-D-25-46793
**Article Type:**	Research Article
**Full Title:**	Exploration of neuropeptides to identify potential target for regulating feeding behavior and development in Eurygaster integriceps
**Short Title:**	Exploration of neuropeptides in Eurygaster integriceps
**Corresponding Author:**	Mohammad Reza Ghaffari, Associate Professor<br>Agricultural Biotechnology Research Institute of Iran<br>Karaj, Alborz IRAN, ISLAMIC REPUBLIC OF
**Keywords:**	Biocontrol; Neuropeptides; Sunn pest
**Abstract:**	Neuropeptides regulate a wide array of vital biological processes including feeding, reproduction and development. Consequently, they have garnered significant attention as potential species-specific insect control agents. However, research on neuropeptides and targeted feeding mechanisms that directly impact insect pest survival and population dynamics remains limited. This study investigated neuropeptide expression patterns and structure in Sunn pests (Eurygaster integriceps Puton (Hemiptera: Scutelleridae)) at three different developmental levels, sexes, and organs using RNA-seq data. Thirteen of the 46 neuropeptides identified in this study encode feeding and digestive physiology neuropeptides. An analysis of the expression patterns of these genes in males and females revealed that neuropeptides were predominantly expressed in females than in males, contributing to sexually dimorphic physiological responses. Furthermore, most neuropeptides were found to be expressed more in the head than in the gut, suggesting that they may act as neuro-regulators. Moreover, analysis of neuropeptide expression patterns during the life cycle of Sunn pest revealed the highest expression in early stages and the lowest in later stages, along with stage-specific variations. A coordinated control mechanism over critical physiological processes may be revealed by these findings, which suggest a possible crosstalk between signaling pathways that regulate growth, metabolism, feeding behavior, and gut function at different stages. The findings of this study suggest promising avenues for future insect pest management research using novel neuropeptide-based biocontrols.
**Order of Authors:**	Mehrbanoo Kazemi Alamouti
	Mohammad Majdi, Associate Professor
	Reza Talebi, Assistant Professor
	Mehdi Dastranj
	Aziz Sheikhigarjan, Associate Professor
	Mohsen Mardi, Full Professor
	Alireza Bandani, Associate Professor
	Zahra Ghorbanzadeh
	Ghasem Hossini Salekdeh, Full Professor
	Mohammad Reza Ghaffari, Associate Professor
**Additional Information:**	
**Question**	**Response**
**Financial Disclosure**<br><br>Enter a financial disclosure statement that	The author(s) received no specific funding for this work.

Powered by Editorial Manager® and ProduXion Manager® from Aries Systems Corporation

describes the sources of funding for the work included in this submission. Review the submission guidelines for detailed requirements. View published research articles from *PLOS ONE* for specific examples.<br><br>This statement is required for submission and **will appear in the published article** if the submission is accepted. Please make sure it is accurate.<br><br>**Funded studies**<br>Enter a statement with the following details:<br>• Initials of the authors who received each award<br>• Grant numbers awarded to each author<br>• The full name of each funder<br>• URL of each funder website<br>• Did the sponsors or funders play any role in the study design, data collection and analysis, decision to publish, or preparation of the manuscript?<br><br>Did you receive funding for this work?	
**Competing Interests**<br><br>Use the instructions below to enter a competing interest statement for this submission. On behalf of all authors, disclose any competing interests that could be perceived to bias this work—acknowledging all financial support and any other relevant financial or non-financial competing interests.<br><br>This statement is required for submission and **will appear in the published article** if the submission is accepted. Please make sure it is accurate and that any funding sources listed in your Funding Information later in the submission form are also declared in your Financial Disclosure statement.<br><br>View published research articles from *PLOS ONE* for specific examples.	The authors have declared that no competing interests exist.

Powered by Editorial Manager® and ProduXion Manager® from Aries Systems Corporation

**NO authors have competing interests**<br><br>Enter: *The authors have declared that no competing interests exist.*<br><br>**Authors with competing interests**<br><br>Enter competing interest details beginning with this statement:<br><br>*I have read the journal's policy and the authors of this manuscript have the following competing interests: [insert competing interests here]*<br><br>* typeset	
**Ethics Statement**<br><br>Enter an ethics statement for this submission. This statement is required if the study involved:<br><br>• Human participants<br>• Human specimens or tissue<br>• Vertebrate animals or cephalopods<br>• Vertebrate embryos or tissues<br>• Field research<br><br>Write "N/A" if the submission does not require an ethics statement.<br><br>General guidance is provided below. Consult the submission guidelines for detailed instructions. **Make sure that all information entered here is included in the Methods section of the manuscript.**	N/A

**Format for specific study types**<br><br>**Human Subject Research (involving human participants and/or tissue)**<br>• Give the name of the institutional review board or ethics committee that approved the study<br>• Include the approval number and/or a statement indicating approval of this research<br>• Indicate the form of consent obtained (written/oral) or the reason that consent was not obtained (e.g. the data were analyzed anonymously)<br><br>**Animal Research (involving vertebrate animals, embryos or tissues)**<br>• Provide the name of the Institutional Animal Care and Use Committee (IACUC) or other relevant ethics board that reviewed the study protocol, and indicate whether they approved this research or granted a formal waiver of ethical approval<br>• Include an approval number if one was obtained<br>• If the study involved *non-human primates*, add *additional details* about animal welfare and steps taken to ameliorate suffering<br>• If anesthesia, euthanasia, or any kind of animal sacrifice is part of the study, include briefly which substances and/or methods were applied<br><br>**Field Research**<br><br>Include the following details if this study involves the collection of plant, animal, or other materials from a natural setting:<br>• Field permit number<br>• Name of the institution or relevant body that granted permission	
**Data Availability**<br><br>Authors are required to make all data underlying the findings described fully available, without restriction, and from the time of publication. PLOS allows rare exceptions to address legal and ethical concerns. See the PLOS Data Policy and FAQ for detailed information.	Yes - all data are fully available without restriction

A Data Availability Statement describing where the data can be found is required at submission. Your answers to this question constitute the Data Availability Statement and **will be published in the article**, if accepted.<br><br>**Important:** Stating 'data available on request from the author' is not sufficient. If your data are only available upon request, select 'No' for the first question and explain your exceptional situation in the text box.<br><br>Do the authors confirm that all data underlying the findings described in their manuscript are fully available without restriction?	
**Describe where the data may be found in full sentences. If you are copying our sample text, replace any instances of XXX with the appropriate details.**<br><br>• If the data are **held or will be held in a public repository**, include URLs, accession numbers or DOIs. If this information will only be available after acceptance, indicate this by ticking the box below. For example: *All XXX files are available from the XXX database (accession number(s) XXX, XXX.).*<br>• If the data are all contained **within the manuscript and/or Supporting Information files**, enter the following: *All relevant data are within the manuscript and its Supporting Information files.*<br>• If neither of these applies but you are able to provide **details of access elsewhere**, with or without limitations, please do so. For example:<br><br>*Data cannot be shared publicly because of [XXX]. Data are available from the XXX Institutional Data Access / Ethics Committee (contact via XXX) for researchers who meet the criteria for access to confidential data.*<br><br>*The data underlying the results presented in the study are available from (include the name of the third party*	All relevant data are within the manuscript and its Supporting Information files

and contact information or URL).<br>• This text is appropriate if the data are owned by a third party and authors do not have permission to share the data.<br><br>* typeset	
Additional data availability information:	

Edited_Final_Kazemi_2025 Click here to access/download;Manuscript;Edited_Final_Kazemi_2025.docx

Exploration of neuropeptides to identify potential target for regulating feeding behavior and development in *Eurygaster integriceps*

Mehrbanoo Kazemi Alamouti[1,2], Mohammad Majdi[2,*], Reza Talebi[1,3], Mehdi Dastranj[4], Aziz Sheikhigarjan[5], Mohsen Mardi[1], Alireza Bandani[5], Zahra Ghorbanzadeh[1,6], Ghasem Hossini Salekdeh[7], Mohammad Reza Ghaffari[1,6,*]

[1]Department of Systems and Synthetic Biology, Agricultural Biotechnology Research Institute of Iran, Agricultural Research, Education and Extension Organization (AREEO), Karaj, Iran

[2]Department of Plant Genetics and Production, Faculty of Agriculture, University of Kurdistan, Sanandaj, Iran

[3]Department of Animal Genomics and Bioinformatics, Animal Biotechnology Research Institute, Agricultural Research, Education and Extension Organization (AREEO), Agricultural Biotechnology Research Institute of Iran (ABRII), Rasht, Iran

[4]Department of Agricultural Entomology, Iranian Research Institute of Plant Protection, Agricultural Research, Education and Extension Organization (AREEO), Tehran, Iran

[5]Department of Plant Protection, College of Agriculture and Natural Resources, University of Tehran, Karaj, Iran

[6]Department of Bioinformatics, Agricultural Biotechnology Research Institute of Iran, Agricultural Research, Education and Extension Organization (AREEO), Karaj, Iran

[7]School of Natural Sciences, Macquarie University, Macquarie Park, NSW 2109, NSW

ORCID
0009-0005-2456-7321 (M.Kazemi Alamuti)
0000-0001-6959-4845 (M.Majdi)
0000-0002-5555-8453 (R. Talebi)
0000-0002-5124-4721 (G. Hossini Salekdeh)
0000-0002-7139-8613 (M.R. Ghaffari)

*Corresponding authors:
Mohammad Majdi: majdi60@gmail.com, m.majdi@uok.ac.ir
Mohammad Reza Ghaffari: mrghaffari52@gmail.com.

Abstract

Neuropeptides regulate a wide array of vital biological processes including feeding, reproduction and development. Consequently, they have garnered significant attention as potential species-specific insect control agents. However, research on neuropeptides and targeted feeding mechanisms that directly impact insect pest survival and population dynamics remains limited. ~~This study investigated neuropeptide expression patterns and structure~~ in Sunn pests (*Eurygaster integriceps* Puton (Hemiptera: Scutelleridae)) at three different developmental levels, sexes, and organs using RNA-seq data. Thirteen of the 46 neuropeptides identified in this study encode feeding and digestive physiology neuropeptides. An analysis of the expression patterns of these genes in males and females revealed that neuropeptides were predominantly expressed in females than in males, contributing to sexually dimorphic physiological responses. Furthermore, most neuropeptides were found to be expressed more in the head than in the gut, suggesting that they may act as neuro-regulators. Moreover, analysis of neuropeptide expression patterns during the life cycle of Sunn pest revealed the highest expression in early stages and the lowest in later stages, along with stage-specific variations. A coordinated control mechanism over critical physiological processes may be revealed by these findings, which suggest a possible crosstalk between signaling pathways that regulate growth, metabolism, feeding behavior, and gut function at different stages. The findings of this study suggest promising avenues for future insect pest management research using novel neuropeptide-based biocontrols.

Keywords

Biocontrol; Neuropeptides; Sunn pest

Introduction

The Sunn pest, *Eurygaster integriceps*, is a major agricultural pest of wheat and barley in the West and Central Asia and Eastern Europe, causing devastating economic losses [1]. Yield losses attributed to heavy infestations of Sunn pest in two key crops ranges from 50–90% (wheat) and 20–30% (barley) with the possibility of complete crop failure without any effective pest control [2,3]. For instance, in Iran, infestations lead to over 9 million tons of qualitative and quantitative losses annually, severely impacting food security and economic stability [4]. These severe losses further highlight the critical need for targeted pest management strategies to mitigate the damage caused by *E. integriceps*.

One promising avenue for such strategies involves disrupting the insect's fundamental physiological processes. In general, insects have various strategies to obtain their required nutrients and energy [5]. Energy homeostasis is vital for survival, growth, and reproduction of animal species [6]. Energy deprivation drives a complex behavioral program to ensure adequate food intake [7]. Feeding behavior in insects has been investigated in terms of physiology, biology and biochemistry [5]. Physiological aspects of feeding behavior indicate that special components of the nervous system are involved in regulating feeding behavior in insects. Neuropeptides can be defined as regulatory peptides with functional roles in growth and development, behavior and reproduction, metabolism, homeostasis, and muscle movement [8,9]. They can be produced in most major types of neurons, including sensory cells/neurons, interneurons, and motoneurons. Hormonal peptides are produced both in neurosecretory cells of the central nervous system (CNS) and in peripheral secretory (neurosecretory and endocrine)

cells, hence signaling can occur bi-directionally from the CNS to periphery and vice versa [10–12]. Due to the high specificity of neuropeptides and their cognate receptors, they can be used to design a selective insecticidal target system for reducing the fitness of target pest insects, while minimizing detrimental environmental impacts [13,14].

However, the role of neuropeptides, their mechanisms of action, interactions with each other, and their release are still poorly understood. In our previous study, we identified neuropeptide precursors in *E. integriceps* through transcriptome analysis [15]. We conducted structural analysis and predicted the mature peptides of neuropeptides involved in various feeding mechanisms. Building on these findings, this study aims to further explore these neuropeptides by examining their structural properties, expression patterns over different developmental stages, sexes and organs. ~~By gaining deeper understanding of the neuropeptide network that modulates pest feeding behavior, this research may pave the way for establishing neuropeptide-based pest control strategies~~.

Materials and methods

Insect rearing and sample preparation

In early April 2023, adult Sunn pest (*E. integriceps*) specimens were collected from wheat fields at the Varamin Research field, Tehran Province, Iran (35.3252° N, 51.6472° E). The collected population was reared on freshly cut wheat and provided with water in a growth chamber at the Department of Systems Biology, Agricultural Biotechnology Research Institute of Iran (ABRII), Karaj, Alborz Province, Iran. The chamber was maintained under controlled conditions with a long-day photoperiod (16 h light/8 h dark), a temperature of 26 ± 1 °C, and relative humidity of 60 ± 10% [4].

The developmental stages of *E. integriceps* included eggs, 1st, 2nd, 3rd, 4th, and 5th instar nymphs, as well as female and male adults. The sample groups consisted of more than 100 eggs, 30 individuals for each of the 1st to 3rd instar nymph stages, and 10 individuals for each of the 4th and 5th instar nymphs, female adults, and male adults.

For organ-specific analysis, female and male adults were dissected to isolate heads and guts. A total of 15 heads and 15 guts were prepared. Adult Sunn pests were immobilized on a clean wax plate using insect pins, and their epidermis was carefully removed. The dissected organs were placed in sterilized glass containers, rinsed twice with DEPC-treated water, and separated using microscopic tweezers under a stereomicroscope (Nikon SMZ1500, Japan). The isolated organs were immediately transferred to liquid nitrogen for freezing and stored at −80 °C until further analysis.

Bioinformatics analysis

The data analyzed in this study were derived from our previous publication [15]. The structures of mature peptides were predicted using a well-established workflow [16,17]. Briefly, prohormone cleavage sites were identified based on the criteria outlined by Veenstra [18] and through homology with known pro-peptide processing schemes. Each deduced precursor was assessed for the presence of a signal peptide using the SignalP 5 online program (http://www.cbs.dtu.dk/services/SignalP/). Protein domain structures were illustrated using the Domain Graph (DOG, version 2.0) software. Multiple sequence alignments of the predicted peptide sequences were performed using ClustalX. Motif identification and sequence logo diagrams were generated with the online tool WebLogo [19]. Post-translational modifications,

such as N-terminal cyclization of Gln/Glu residues (Q/E) and C-terminal amidation at Gly residues (G), were predicted based on homology with known peptide isoforms [20].

Expression Pattern Analysis

Total RNA was extracted from various *E. integriceps* samples using TRIzol reagent (Invitrogen, Carlsbad, CA, USA) according to the manufacturer's protocol. The purity and integrity of the extracted RNA were evaluated using a NanoDrop 2000 spectrophotometer (Thermo Scientific, Wilmington, DE, USA). To eliminate genomic DNA contamination, the RNA samples were treated with DNase I (Fermentas, Madison, WI, USA). Reverse transcription was performed using the PrimeScript RT Reagent Kit (Takara, Dalian, Liaoning, China) in a 20 μL reaction mixture containing 1 μg of total RNA. The resulting cDNA was serially diluted 2-fold for the analysis of RT-qPCR primer specificity. Quantitative primers were designed using Oligo7 software [21] and Primer3 (https://bioinfo.ut.ee/) based on the obtained nucleotide sequences. Primer sequences are provided in Table S1). RT-qPCR analysis was conducted on a LightCycler 480 system (Roche, Switzerland) using a two-step amplification protocol: 95°C for 30 seconds, followed by 40 cycles of 95°C for 5 seconds and 60°C for 30 seconds. Melting curve analysis was performed to confirm primer specificity. Each reaction mix consisted of 5.0 μL of SinaSYBR Green QPCR Master Mix (Sinaclon), 3.9 μL of nuclease-free water, 0.6 μL of forward and reverse primers (10 μmol/L), and 0.5 μL of cDNA. The *18S rRNA* gene (KP890857) was used as an internal reference [22]. Each experiment included three biological replicates and two technical replicates. Relative mRNA expression levels were calculated using the $2^{-\Delta\Delta CT}$ method [23].

Statistical analysis

The relative gene expression levels from various samples were measured as mean ± standard error (SE). Data were analyzed using one-way analysis of variance (ANOVA), followed by Tukey's honestly significant difference (HSD) post hoc test for multiple comparisons at a significance level of $P < 0.05$. Statistical analyses were conducted using SPSS version 27.0 (IBM Corp., Armonk, NY, USA).

Results

Neuropeptides discovery by transcriptome mining: bioinformatics analyses and peptide prediction

Through transcriptome mining and bioinformatics analyses, three types of allatostatin precursors including A-type (Ast-A), B-type (Ast-B), and C-type (Ast-C) were identified, each exhibiting unique structural features and conserved motifs (Fig 1). The precursor of Ast-A was predicted to be 212 amino acids (aa) in length and encode eight mature peptides. These peptides are separated by dibasic cleavage sites and terminate with a glycine residue, enabling C-terminal amidation. Most mature peptides share the conserved C-terminal motif XYXFGL amide, where X represents variable residues. Despite this conserved motif, each predicted peptide differs in sequence and length, reflecting their structural variability. For Ast-B, the precursor was found to comprise 174 aa, starting with a 21-aa signal peptide (Fig 1). It encodes seven mature peptides, ranging from 9 to 11 aa in length, which are separated by dibasic cleavage sites. These peptides share a conserved XWXXXXGXW amide motif at their C-terminus, highlighting their functional consistency while maintaining some sequence variability. The Ast-C precursor was identified as

a shorter sequence of 63 aa encoding a single mature peptide. This peptide is 14 aa long and features a conserved amidated C-terminal motif, -PISCF (Fig 1). The peptide structure is stabilized by a disulfide bridge formed between cysteine residues at positions 6 and 13. This structural arrangement is characteristic of C-type allatostatins [24]. The mature peptides encoded by allatostatin A, B, and C end with a glycine residue, which facilitates amidation at the C-terminus [25].

Fig. 1. Identification and characterization of *Eurygaster integriceps* ASTs peptides.

(A-type allatostatin) Schematic representation of Ast-A precursor and their 8 mature peptides comparative sequence alignments in *E. integriceps*, sequence logo is shown above alignments. (B-type allatostatin) Amino acid sequences and schematic representation of Ast-B precursor in *E. integriceps*, logo is shown above comparative sequence alignments of Ast-B peptides. (C-type allatostatin) Schematic representation of Ast-C in *E. integriceps* and their mature peptides comparative sequence alignments, sequence logo is shown above alignments.

Transcriptome mining revealed three adipokinetic hormone (AKH) precursors (AKH-1, AKH-2, and AKH-3) and one bursicon (Burs) precursor, each displaying distinct structural characteristics and conserved motifs (Fig 2). The AKH precursors were predicted to encode 65, 67, and 120 (aa) for AKH-1, AKH-2, and AKH-3, respectively. AKH-1 contains a single predicted peptide that is separated by a dibasic cleavage site, consistent with neuropeptide maturation (Fig 2). Both AKH-2 and AKH-3 feature a signal peptide, 19 aa long, respectively, followed by mature peptide regions. The mature peptides encoded by AKH-2 and AKH-3 are 8–10 aa in length and terminate with a glycine residue, enabling amidation of the C-terminal (Fig 2). Notably, AKH-3 encodes two mature peptides, highlighting its structural complexity. These peptides display a conserved

C-terminal motif, characteristic of AKH peptides (Fig 2). Burs is 132 aa long and begins with an 18 aa signal peptide (Fig 2). Unlike the AKH peptides, the mature peptide encoded by Burs lacks a glycine residue at the C-terminal, which precludes amidation. This precursor shares 89% sequence identity with the Burs precursor of the Oriental Stink Bug, *Plautia stali* (Table S2), demonstrating evolutionary conservation.

Fig. 2. Identification and characterization of *Eurygaster integriceps*Adipokinetic hormone and Bursicon.

AKH Schematics showing three AKH precursors identified in *E. integriceps*. Comparative sequence alignment of AKH mature peptides, sequence logos are shown above alignments. Burs Schematics showing Burs precursor identified in *E. integriceps*.

The predicted sequence of the CCHamide (CCH) precursor is composed of 95 aa with a predicted signal peptide of 22 aa (Fig 3). The mature peptide, a C-terminal amidated nonapeptide, starts at position 45 and ends at position 52, containing two cysteine residues. This conserved structure, GCXSFGHSCXGGHamide, highlights the functional conservation of the CCH mature peptide [26] (Fig 3). Two transcripts were identified to putatively encode a complete Corazonin precursor, consisting of 99 aa (Crz-1) and 80 aa (Crz-2), respectively (Fig 3). Crz-1 contains a predicted signal peptide of 27 aa, whereas Crz-2 has a 11 aa signal peptide. Both are followed by 11 predicted mature peptides, separated by dibasic cleavage sites. The mature peptides share a conserved sequence: QTFQYSRGWTN amide (Fig 3).

Fig. 3. Identification and characterization of CCHamide and Corazonin in *Eurygaster integriceps*.

Schematic showing CCH and two Crz precursors identified in *E. integriceps*. A sequence logo is shown above alignments, where the height of each letter is proportional to the observed frequency of the corresponding amino acid in the alignment column.

The predicted ecdysis-triggering hormone precursor (ETH) consists of 149 aa, including a 23 aa signal peptide (Fig 4). This is followed by three predicted ETH peptides of 14 aa each, separated by dibasic cleavage sites (Fig 4). All three ETH peptides share high similarity with the conserved motif VPRLamide (Fig 4), which is characteristic of the ETH family possessing the C-terminal motif -PRXamide. This motif is widely conserved in invertebrates [27]. One transcript was identified that potentially encodes a complete ion transport peptide-like isoform (ITP) consisting of 183 aa, which contains a dibasic cleavage site and lacks the glycine necessary for amidation of the peptide. The mature ITP peptide, comprising 79 aa, includes 6 cysteine residues (Fig 4). This is consistent with the ITP from *Halyomorpha halys* (Table S2), which also contains six cysteine residues in its mature peptide, with these cysteines being well-aligned. The ITP precursor showed a 99% identity with the ITP from the brown marmorated stink bug (*H. halys*).

Fig. 4. Identification and characterization of Ecdysis triggering hormone and Ion transport peptide in *Eurygaster integriceps*.

Schematic showing ETH and ITP precursors and mature peptide identified in *E. integriceps*. A sequence logo is shown above alignments, where the height of each letter is proportional to the observed frequency of the corresponding amino acid in the alignment column.

The nucleotide sequence produced from the transcriptome data encodes a complete neuropeptide F precursor (NPF) with 104 aa, beginning with a 26 aa signal peptide (Fig 5). The predicted 29-aa mature peptide contains the conserved RPRFamide motif and shares the highest BLAST similarity with the neuropeptide F of *Rhodnius prolixus* (Table S2). One transcript was identified that encodes a complete short Neuropeptide F precursor (sNPF) consisting of 89 aa, starting with a 34 aa signal peptide. This is followed by a single predicted peptide, 12 aa in length, containing the conserved XPXRLRFamide motif (Fig 5). The LOGO analysis of NPF and sNPF confirms their conserved C-terminal motif: RXRFamide (Fig 5). The search for Pigment-dispersing factor precursors (PDF) revealed a transcript encoding a complete 85 aa precursor. This precursor starts with a 24 aa signal peptide and contains a mature peptide of 18 aa, separated by dibasic cleavage sites (Fig 5). The PDF precursor shares over 80% identity with the PDF of the Southern green stink bug, *Nezara viridula* (Table S2). In the transcriptome assembly, a single transcript encodes a complete SIF amide precursor (SIF) comprising 76 aa, beginning with a 25 aa signal peptide. The predicted mature peptide, GYRKPPFNGSIFamide, is located immediately after the signal peptide (Fig 5). The SIF precursor shows high homology (93% identity) with the SIF of the Oriental Stink Bug, *P. stali* (Table S2). Additionally, the mature SIF peptide displays a conserved structure: XYRKPPFNGSIFamide.

Fig. 5. Identification and characterization of Neuropeptide F, Short Neuropeptide F, Pigment dispersing factor and SIFamide in *Eurygaster integriceps*. Schematic showing NPF, sNPF, PDF and SIF precursors and mature peptide identified in *E. integriceps*. A sequence logo is shown above alignments, where the height of each letter is proportional to the observed frequency of the corresponding amino acid in the alignment column.

Neuropeptide Expression Profile

Expression Profile of Neuropeptide During Development

The gene expression in *E. integriceps* revealed dynamic fluctuations across developmental stages (Fig 6). The Highest expression levels were predominantly found in early instars (Sunn pest Instar (SI) 1-SI2), particularly for *Ast-A*, *Ast-B, CCH, AKH, Crz*, and *sNPF*. Later stages (SI3-SI5) generally exhibited lower expression, except for genes like *PDF* and *ITP*, which showed an initial decline in SI3 followed by an increase in SI4. *Ast-C* and *AKH* showed unique expression peaks in SI2 indicating stage-specific regulatory roles. Although various types of allatostatins (*Ast-A, Ast-B, Ast-C*), are expressed in all developmental stages of *E. integriceps* (S1 Fig), there are differences in their expression levels across stages. *Ast-A* and *Ast-B* exhibited higher expression during the 1st instar nymph stage compared to later stages, while *Ast-C* was predominantly expressed in eggs, showing a 3.2-fold increase compared to all other stages (S1 Fig). *CCH* and *AKH*, *CCH* was highly expressed in SI1 (2.9-fold higher than in SI5). *AKH* peaked in SI2, with expression levels nearly 3.1-fold higher than in eggs. *PDF* and *ITP* showed a similar trend. Expression levels decreased significantly in SI3 (1.8-fold lower than in SI1) and increased again in SI4 (2.4-fold higher compared to SI3). *Burs* exhibited the highest expression in SI2, with a 2.6-fold increase compared to S5. *ETH,* which is crucial for initiating molting, showed the highest level in SI1 (3.0-fold increase compared to eggs) and remained relatively stable across later stages [28]. (*sNPF* and *NPF*), *sNPF* and *NPF* had peak expression in SI1, with *sNPF* being 3.5-fold higher and *NPF* 2.9-fold higher than in SI5.

Fig. 6. Expression patterns of 13 neuropeptide genes across developmental stages and between sexes in *Eurygaster integriceps*.

This figure illustrates the relative expression levels of 13 neuropeptide genes at different developmental stages of *E.integriceps*, from egg to the 5th instar nymph, as well as in adult males and females. The heatmaps represent gene expression profiles, with red indicating upregulation and blue indicating downregulation. The developmental stages include egg, 1st to 5th instar nymphs (SI1–SI5), and adult males and females. The expression levels of these genes were analyzed to understand their potential role in growth, development, and sex-specific physiological processes. The color scale at the bottom right shows expression intensity values.

The expression profile of adult females and males

The expression profiles of 13 neuropeptides in adult *E. integriceps* were analyzed to identify sex-specific differences (Fig 6) (S2 Fig). The results revealed significant variation in the expression levels of multiple neuropeptides between males and females of certain neuropeptides being preferentially expressed in one sex. Overall, the expression levels of *Ast-A*, *Ast-B*, *Ast-C*, *AKH*, *SIF*, *ITP*, *Burs*, *sNPF*, *NPF*, and *Crz* were significantly higher in males compared to females. In contrast, *CCH* and *PDF* were expressed at higher levels in females. No significant difference was found in *ETH* expression between the two sexes.

Organ-specific Expression Profile

The organ-specific expression profiles of 13 neuropeptide precursors in *E. integriceps* adults, analyzed across two organs including the head and gut (Fig 7) (S3 Fig). The results showed that

all neuropeptides were expressed in the head. In contrast certain neuropeptides associated with central nervous system, including *SIF*, *NPF*, *Crz*, and *AKH*, were not detected in the gut. The neuropeptides that were detected in both organs comprising of *Ast-A*, *Ast-C*, and *CCH* exhibited significantly higher expression in the gut compared to the head. Conversely, *Ast-B*, *PDF*, *ITP*, *sNPF*, and *ETH* were predominantly expressed in the head (Fig 7). Interestingly, *Burs* did not show any significant difference in expression between the head and gut, implying that its function may be distributed across both organs or may be regulated post-transcriptionally rather than at the expression level.

Fig. 7. Comparative expression of 13 neuropeptide genes in the head and gut of adult *Eurygaster integriceps*.

This figure illustrates the comparative expression levels of 13 neuropeptide genes in the head and gut of adult *E. integriceps*. The anatomical diagram highlights the head and gut, with bar graphs representing gene expression levels in each organ. Bars positioned at the top represent the head, while those at the bottom represent the gut. The variation in expression levels suggests potential organ-specific functions of these neuropeptides in physiological and regulatory processes.

Discussion

Neuropeptides and peptide hormones are essential for cell-to-cell communication in multicellular animals [29,30]. They are derived from precursor proteins that undergo posttranslational modifications such as amidation that make them biologically active [31,32]. Neuropeptides and their receptors are highly conserved, playing critical roles in reproduction, metabolism, growth, and locomotion. [29,33]. Due to their physiological significance, neuropeptides have been

suggested as potential insecticidal targets [34]. The expression of neuropeptides in the *E. integriceps* is, however, poorly studied. In this study, the structure and expression patterns of identified neuropeptides involved in feeding regulation are examined across developmental stages, organs (head and gut), and adult males and females of *E. integriceps*.

Neuropeptides Identification and Structures

Allatostatin peptides play a crucial role in juvenile hormone regulation [35] and are diverse peptides derived from three distinct genes in insects, designated as *Ast-A, Ast-B, and Ast-C* [36]. In this study, the structural characterization of Ast-A, Ast-B, Ast-C peptides revealed both conserved motifs and sequence variability. There are eight mature peptides encoded by the Ast-A precursor, all of which share the characteristic C-terminal motif XYXFGL amide, which represents a hallmark of this family. However, the variable residues (X) in the motif and peptide lengths may be able to fine-tune the interaction with receptor subtypes, facilitating functional plasticity across tissues or developmental stages. Similarly, Ast-B peptides in Sunn pest exhibit a conserved XWXXXXGXW amide motif, which aligns with their role in regulating juvenile hormone synthesis like other insects. The shorter and more uniform length of Ast-B peptides (9–11 aa) compared to Ast-A (212 aa) may reflect distinct release dynamics or receptor-binding kinetics.

AKH, a key neuropeptide, regulates lipid and carbohydrate levels, energy metabolism, and the female reproductive system in insects [37]. In *E. integriceps*, we identified three neuropeptide precursors, two of which possess signal peptides (Fig 2). Prior studies have demonstrated that injecting mature AKH peptide elevates carbohydrate and lipid levels in *Bombyx mori* hemolymph [38]. Similarly, in *Coccinella septempunctata*, CsAKH and CsAKHR control lipid

storage and ovarian development during diapause [39]. The presence of two signal peptides in AKH precursors suggests that these peptides are secreted and act as endocrine or paracrine signals, aligning with AKH's established mode of action, where it is released from the corpora cardiaca into the hemolymph to exert systemic effects[40,41].

We also identified the CCH precursor, which contains a signal peptide and a conserved GCXSFGHSCXGGH structure (Fig 3), aligning well with CCH found in other insects. For instance, in *Drosophila melanogaster*, CCHamide-1 and CCHamide-2 are critical for stimulating food intake [42], whereas in *Gryllus bimaculatus*, CCH modulates feeding behavior and nutrient signaling [43]. Furthermore, CCH influences feeding motivation in blowflies and enhances sensory perception and olfactory responses in starved *D. melanogaster* [44]. Therefore, the identified CCH precursor in *E. integriceps* likely plays similar regulatory roles, potentially impacting feeding behavior and reproduction in this species.

ETH, as the identified neuropeptide precursor in *E. integriceps*, and its three mature ETH peptides, showed considerable similarity to the conserved VPRL amide motif (Fig 4). The process of ecdysis in insects requires ETH, an essential endocrine signal [45]. Besides its function in ecdysis, ETH is also associated with reproductive regulation [46]. Prior study showed ETH influences on juvenile hormone-related courtship memory and reproductive viability in several insect species, thus showing its multifaceted properties [47].

An additional significant finding was the identification of the ITP precursor. This precursor contains six spatially conserved cysteines in the mature peptide [44] . These cysteines form three intramolecular disulfide bonds, which stabilize the hormone (Fig 4). ITP is secreted by the corpora cardiaca and induces the ileum to transfer Cl^{-1} ions from the lumen to the hemolymph, establishing an electrochemical gradient that facilitates water reabsorption[48]. Two

neuropeptide F precursors were identified in this research; the mature peptides are 29 and 12 amino acids, respectively, and are referred to as NPF and sNPF (Fig 5). The C-terminal end of both peptides includes the conserved RXRF motif. The neuropeptide NPF in insects, containing 7–40 amino acids and a conserved RPRF amide motif, is critical for its functional activity in feeding, metabolism, reproduction, and stress responses[37,49]. In *Drosophila,* NPF regulates food intake and body size by modulating insulin-like peptides [50].

Developmental Expression Patterns of Neuropeptides

The dynamic expression profiles of neuropeptides throughout the developmental stages of *E. integriceps* provide essential information regarding their functional roles in growth, the regulation of feeding, and molting. The highest levels of neuropeptide expression, notably *Ast-A*, *Ast-B*, *CCH*, *AKH*, *Crz*, and *sNPF*, were detected in early instars (SI1-SI2). This shows their participation in the initial stages of development and feeding. Conversely, the expression of most neuropeptides in later stages (SI3-SI5), excluding *PDF* and *ITP*, showed a biphasic pattern, with an initial decline in SI3 followed by an increase in SI4. These findings could suggest that neuropeptides have stage-specific regulatory functions in *E. integriceps*. As an example, Allatostatins' *Ast-A* and *Ast-B* gene expression peaked during the first instar; in contrast, *Ast-C* was predominantly expressed in eggs, suggesting its role in early embryonic development rather than feeding regulation. The results are in agreement with earlier studies showing that the gene coding for allatostatins controls juvenile hormone metabolism, which affects development and feeding behavior [51]. Earlier findings revealed that Lepidopteran Ast-C feeding resulted in reduced growth and fecundity and induced considerable mortality in *Acyrthosiphon pisum* and *Myzus persicae* [52,53]. Furthermore, Cao et al. found that allatostatin compounds specifically

inhibit the synthesis and secretion of a specific juvenile hormone in *Spodoptera frugiperda*, which affects the ecdysis and growth of insects and leads to mortality [51,54]. *CCH* and *AKH*, which are recognized for their functions in metabolic regulation, exhibited elevated expression in early instars. Elevated *CCH* expression in SI1 relative to SI5 indicates a potential role for *CCH* in early nutritional signaling. *AKH* showed elevated expression in SI2; its expression was significantly higher than in eggs, which suggests *AKH* may be involved in lipid mobilization and energy metabolism, as previously reported [5]. The cerebral expression profiles of *CCH* at the early vitellogenic stage suggested a possible role in promoting vitellogenesis, as previously indicated in *Scylla paramamosain* [55]. Distinctive expression patterns were observed in the *PDF*, *ITP*, *Burs*, and *ETH* genes that regulate molting. *PDF* and *ITP* exhibited a biphasic pattern, declining in SI3 but increasing in SI4, potentially indicating their involvement in circadian rhythm regulation and physiological adaptation during molting [5,56]. *Burs*, which is key to hardening the cuticle post-molting, had a high level of expression in SI2 versus SI5, while *ETH*, which is required for starting molting, showed its maximum expression in SI1 in contrast to eggs. The results indicate that *ETH* is involved in early molting stages, while *PDF* and *ITP* govern the later molting transitions [28]. The *sNPF* and *NPF* neuropeptide F family members demonstrated peak expression in SI1, and *sNPF* and *NPF* levels were significantly greater than those observed in SI5. In later instars, the expression of these genes was significantly reduced, indicating that *NPFs* are essential for controlling feeding behavior primarily in the early stages of development, a pattern consistent with observations in other insect species [12,57]. The initial high expression of *Ast-A*, *Ast-B*, *CCH*, and *sNPF* strengthens the hypothesis that they have a role in stimulating feeding behavior and promoting growth in young instars. In contrast, the delayed

PDF and *ITP* peaks in SI4 could suggest their involvement in circadian and physiological adaptations prior to the ultimate molting stage [5,28].

Sex-Specific Expression of Neuropeptides

Sexual dimorphisms in neuropeptide expression were observed; specifically, *Ast-A*, *Ast-B*, *Ast-C*, *AKH*, *SIF*, *ITP*, *Burs*, *sNPF*, *NPF*, and *Crz* displayed elevated expression in males, while *CCH* and *PDF* were expressed primarily in females. The absence of significant variation in *ETH* expression suggests the conservation of its function across sexes. These findings imply that neuropeptides contribute to sexually dimorphic physiological functions, potentially influencing reproductive processes, metabolism, and behavioral adaptations. A significant difference in the expression of *NPF*, identified in *E. integriceps*, was observed between adult males and females (Fig 6, S2 Fig). Nevertheless, these neuropeptides are essential in the regulation of numerous physiological functions, such as development, reproduction, and feeding [36]. The higher expression of *sNPF* and *NPF* in males may correlate with increased feeding activity and energy metabolism, while heightened *PDF* expression in females might be indicative of a role in circadian rhythm regulation [36]. In this study, *SIF* exhibited higher expression in males (Fig 6). *SIF* are neuropeptide precursors that are highly conserved and involved in regulating sexual behavior, feeding, and sleep [58]. *SIF* knockdown in *R. prolixus* decreased blood meal consumption; however, injection of *Rhopr-SIF* increased blood meal intake [59]. The widespread distribution of *SIF* in the CNS, along with numerous varicosities throughout the ganglia, may indicate its potential involvement in regulating adult functions, such as reproduction [58]. Regarding sexual differences, the release of *SIF* in the brain may inhibit sexual behavior until the flies encounter the appropriate physiological conditions, suggesting a role in sexual dimorphism

[60]. Nevertheless, these inferences necessitate additional validation through tissue localization studies, functional inhibition assays, and other experimental methodologies.

Organ-Specific Expression of Neuropeptides

The organ-specific expression profiles of neuropeptides in *E. integriceps* provide essential data for in-depth investigation of the biology and ecology of this vital biological control species. Most of the *E. integriceps* neuropeptide precursor genes were expressed in the brain (Fig 7, S3 Fig). The data suggests that *Ast-B*, *PDF*, *ITP*, and *ETH* expression was predominant in the brain, implying that most neuropeptide signaling systems act as neuroregulators in predatory bugs [61]. Conversely, *Ast-C* and *CCH* exhibited broad expression within the gut (Fig 7). In *Arma custos*, the gut exhibited high expression levels of *CCH* and *Ast-C* [61]. *Ast-C* and *CCH* are brain-gut peptides that are conserved and that have functions such as myotropic effects and feeding behavior regulation [62]. In insects, neuropeptides within the gut control feeding behavior, which includes the selection of food, appetite regulation, digestion, metabolism, and excretion [58]. Notably, *SIF*, *AKH*, *NPF*, and *Crz* expression was confined to the brain (Fig 7), which aligns with observations in other insect species [44,63,64]. SIFamide-like immunoreactivity has been localized in the CNS of *R. prolixus* [65]. *SIF* is produced exclusively by four interneurons that project throughout the brain [64]. *AKH* is expressed in endocrine cells within the two neurohemal organs, corpora cardiaca, frequently fused and located near the insect brain.

Conclusions

This study provided a comprehensive characterization of neuropeptides regulating feeding behavior and development in Sunn pest (*E. integriceps*) which is a devastating pest of wheat in

Iran. Transcriptomic analysis and expression profiling facilitated the identification of 13 neuropeptides, each exhibiting unique temporal, sexual, and tissue-specific expression profiles. The increased expression of *Ast-A*, *Ast-B*, *CCH*, *AKH*, *Crz*, and *sNPF* in early instars supports their significant contribution to nymphal feeding and growth, while the biphasic expression pattern of *PDF* and *ITP* suggests these neuropeptides' involvement in molting and circadian regulation. It is noteworthy that the expression of most neuropeptides differs between the sexes, with males expressing more than females (*CCH* and *PDF*), which suggests specialized roles in reproduction and metabolism. The primary brain localization of most neuropeptides supports their neuro-regulatory roles, though the gut-specific expression of *Ast-C* and *CCH* suggests further roles in digestive physiology. According to these findings, neuropeptide signaling can potentially be used to control pests specifically adapted to a species through a process known as neuropeptide signaling. In order to develop sustainable alternatives to conventional insecticides within integrated pest management strategies, future research must focus on functionally characterizing these neuropeptides and their receptor binding in order to confirm their involvement in feeding and development.

Supporting information

Table S1. List of primers used for gene expression analysis.

Table S2. Identified neuropeptide precursors in Eurygaster integriceps.

Table S3. Neuropeptide Genes of *Eurygaster integriceps*: mRNA and Predicted Protein Sequences.

S1 Fig. qRT-PCR results of neuropeptides throughout the Eurygaster integriceps life cycle. The y-axis represents the relative expression level and the x-axis the life cycle. The standard error is represented by the error bar and significant differences are represented by the different letters ($p < 0.05$). E: egg, SI (Sunn pest Inestar)1: 1th-instar nymphs, SI2: 2th-instar nymphs; SI3: 3th instar nymphs; SI4: 4th instar nymphs; SI5: 5th instar nymphs. Ast-A: allatostatin-A; Ast-B: allatostatin-B; Ast-C: allatostatin-C; CCH: CCHamide; AKH: Adipokinetic hormone; SIF: SIFamide; PDF: Pigment-dispersing factor; ITP: Ion transport peptide; Burs: Bursicon; sNPF: Short NPF; NPF: Neuropeptide F; Crz: Corazonin; ETH: Ecdysis triggering hormone. Lowercase letters above the error bars indicate that significant differences among different stages or various tissues ($P < 0.05$, one-way ANOVA followed by Tukey's test).

S2 Fig. Relative expression of neuropeptides in both sexes of Eurygaster integriceps. FS: Female Sunn pest, MS: Male Sunn pest. Ast-A: allatostatin-A; Ast-B: allatostatin-B; Ast-C: allatostatin-C; CCH: CCHamide; AKH: Adipokinetic hormone; SIF: SIFamide; PDF: Pigment-dispersing factor; ITP: Ion transport peptide; Burs: Bursicon; sNPF: Short NPF; NPF: Neuropeptide F; Crz: Corazonin; ETH: Ecdysis triggering hormone. Data are expressed as mean ± standard error (SE). Differences between groups were assessed using one-way ANOVA followed by a t-test. Asterisks denote statistically significant differences: *$p < 0.05$; **$p < 0.01$; ***$p < 0.001$.

S3 Fig. The organ-specific transcript abundances of E. integriceps neuropeptides. Ast-A: allatostatin-A; Ast-B: allatostatin-B; Ast-C: allatostatin-C; CCH: CCHamide; AKH: Adipokinetic hormone; SIF: SIFamide; PDF: Pigment-dispersing factor; ITP: Ion transport

peptide; Burs: Bursicon; sNPF: Short NPF; NPF: Neuropeptide F; Crz: Corazonin; ETH: Ecdysis triggering hormone. Data are expressed as mean ± standard error (SE). Differences between groups were assessed using one-way ANOVA followed by a t-test. Asterisks denote statistically significant differences: *$p < 0.05$; **$p < 0.01$; ***$p < 0.001$.

Author contributions

Conceptualization: M.R.G.; Data curation: M.K.A., M.M., and M.R.G.; Formal analysis: M.K.A., M.D., and M.R.G.; Funding acquisition: M.R.G.; Methodology: M.K.A., R.T., M.D., and M.R.G.; Project administration: M.R.G.; Resources: M.K.A., M.M., and M.R.G.; Software: M.K.A., R.T., M.D., and M.R.G.; Supervision: M.R.G.; Validation: M.M. and M.R.G.; Visualization: M.K.A. and M.R.G.; Writing – original draft: M.K.A. and M.R.G.; Writing – review & editing: M.K.A., M.M., R.T., M.D., A.S., M.M., A.B., Z.G., G.H.S., and M.R.G.

References

1. Duman M, Alaserhat İ. Assessment of Eurygaster integriceps parameters to improve integrated pest management in Southeastern Türkiye wheat fields. Sci Rep. 2025;15. doi:10.1038/S41598-025-10340-3
2. Darkoh C, El-Bouhssini M, Baum M, Clack B. Characterization of a prolyl endoprotease from Eurygaster integriceps Puton (Sunn pest) infested wheat. Arch Insect Biochem Physiol. 2010;74: 163–178. doi:10.1002/arch.20370
3. Kivan M, Kilic N. Age-specific fecundity and life table of Trissolcus semistriatus, an egg parasitoid of the sunn pest Eurygaster integriceps. Entomol Sci. 2006;9: 39–46. doi:10.1111/J.1479-8298.2006.00152.X
4. Amiri A, Bandani AR, Alizadeh H. MOlecular Identification Of Cysteine And Trypsin Protease, Effect Of Different Hosts On Protease Expression, And Rnai Mediated Silencing Of Cysteine Protease Gene In The Sunn Pest. Arch Insect Biochem Physiol. 2016;91: 189–209. doi:10.1002/arch.21311
5. Nagata S, Zhou YJ. Feeding-modulating neuropeptides and peptide hormones in insects. Adv In Insect Phys. 2019;57: 137–172. doi:10.1016/BS.AIIP.2019.10.002
6. Gautron L, Elmquist JK, Williams KW. Neural control of energy balance: translating circuits to therapies. Cell. 2015;161: 133–145. doi:10.1016/J.CELL.2015.02.023
7. Sternson SM, Eiselt AK. Three Pillars for the Neural Control of Appetite. Annu Rev Physiol. 2017;79: 401–423. doi:10.1146/ANNUREV-PHYSIOL-021115-104948
8. Altstein M, Nässel DR. Neuropeptide signaling in insects. Adv Exp Med Biol. 2010;692: 155–165. doi:10.1007/978-1-4419-6902-6_8
9. Williams EA. Function and Distribution of the Wamide Neuropeptide Superfamily in

Metazoans. Front Endocrinol (Lausanne). 2020;11. doi:10.3389/FENDO.2020.00344

10. Nässel DR, Zandawala M. Recent advances in neuropeptide signaling in Drosophila, from genes to physiology and behavior. Prog Neurobiol. 2019;179. doi:10.1016/j.pneurobio.2019.02.003

11. Orchard I, Lange AB. The neuroendocrine and endocrine systems in insect – Historical perspective and overview. Mol Cell Endocrinol. 2024;580: 112108. doi:10.1016/J.MCE.2023.112108

12. Schoofs L, De Loof A, Van Hiel MB. Neuropeptides as Regulators of Behavior in Insects. Annu Rev Entomol. 2017;62: 35–52. doi:10.1146/ANNUREV-ENTO-031616-035500

13. Shi Y, Pandit A, Nachman RJ, Christiaens O, Davies SA, Dow JAT, et al. Transcriptome analysis of neuropeptides in the beneficial insect lacewing (Chrysoperla carnea) identifies kinins as a selective pesticide target: a biostable kinin analogue with activity against the peach potato aphid Myzus persicae. J Pest Sci (2004). 2023;96: 253–264. doi:10.1007/s10340-022-01511-6

14. Audsley N, Down RE. G protein coupled receptors as targets for next generation pesticides. Insect Biochem Mol Biol. 2015;67: 27–37. doi:10.1016/j.ibmb.2015.07.014

15. Kazemi Alamouti M, Majdi M, Talebi R, Dastranj M, Bandani A, Hossini Salekdeh G, et al. Transcriptome wide identification of neuropeptides and G protein-coupled receptors (GPCRs) in Sunn pest, Eurygaster integriceps Puton. Gene. 2024;893. doi:10.1016/J.GENE.2023.147911

16. Ahn SJ, Corcoran JA, Vander Meer RK, Choi MY. Identification and Characterization of GPCRs for Pyrokinin and CAPA Peptides in the Brown Marmorated Stink Bug, Halyomorpha halys (Hemiptera: Pentatomidae). Front Physiol. 2020;11.

doi:10.3389/FPHYS.2020.00559

17. Lavore A, Perez-Gianmarco L, Esponda-Behrens N, Palacio V, Catalano MI, Rivera-Pomar R, et al. Nezara viridula (Hemiptera: Pentatomidae) transcriptomic analysis and neuropeptidomics. Sci Rep. 2018;8: 1–15. doi:10.1038/s41598-018-35386-4

18. Veenstra JA. Neuropeptide evolution: Neurohormones and neuropeptides predicted from the genomes of Capitella teleta and Helobdella robusta. Gen Comp Endocrinol. 2011;171: 160–175. doi:10.1016/J.YGCEN.2011.01.005

19. Crooks GE, Hon G, Chandonia JM, Brenner SE. WebLogo: a sequence logo generator. Genome Res. 2004;14: 1188–1190. doi:10.1101/GR.849004

20. Pauls D, Chen J, Reiher W, Vanselow JT, Schlosser A, Kahnt J, et al. Peptidomics and processing of regulatory peptides in the fruit fly Drosophila. EuPA Open Proteomics. 2014;3: 114–127. doi:10.1016/J.EUPROT.2014.02.007

21. Rychlik W. OLIGO 7 primer analysis software. Methods Mol Biol. 2007;402: 35–59. doi:10.1007/978-1-59745-528-2_2

22. Majerowicz D, Alves-Bezerra M, Logullo R, Fonseca-De-Souza AL, Meyer-Fernandes JR, Braz GRC, et al. Looking for reference genes for real-time quantitative PCR experiments in Rhodnius prolixus (Hemiptera: Reduviidae). Insect Mol Biol. 2011;20: 713–722. doi:10.1111/J.1365-2583.2011.01101.X

23. Livak KJ, Schmittgen TD. Analysis of relative gene expression data using real-time quantitative PCR and the 2(-Delta Delta C(T)) Method. Methods. 2001;25: 402–408. doi:10.1006/METH.2001.1262

24. Christie AE, Stemmler EA, Dickinson PS. Crustacean neuropeptides. Cell Mol Life Sci. 2010;67: 4135–4169. doi:10.1007/S00018-010-0482-8

25. Bradbury AF, Finnie MDA, Smyth DG. Mechanism of C-terminal amide formation by pituitary enzymes. Nat 1982 2985875. 1982;298: 686–688. doi:10.1038/298686a0

26. Roller L, Yamanaka N, Watanabe K, Daubnerová I, Žitňan D, Kataoka H, et al. The unique evolution of neuropeptide genes in the silkworm Bombyx mori. Insect Biochem Mol Biol. 2008;38: 1147–1157. doi:10.1016/J.IBMB.2008.04.009

27. Jékely G. Global view of the evolution and diversity of metazoan neuropeptide signaling. Proc Natl Acad Sci U S A. 2013;110: 8702–8707. doi:10.1073/PNAS.1221833110

28. Sterkel M, Volonté M, Albornoz MG, Wulff JP, del Huerto Sánchez M, Terán PM, et al. The role of neuropeptides in regulating ecdysis and reproduction in the hemimetabolous insect Rhodnius prolixus. J Exp Biol. 2022;225. doi:10.1242/JEB.244696

29. Li B, Predel R, Neupert S, Hauser F, Tanaka Y, Cazzamali G, et al. Genomics, transcriptomics, and peptidomics of neuropeptides and protein hormones in the red flour beetle Tribolium castaneum. Genome Res. 2008;18: 113–122. doi:10.1101/gr.6714008

30. Garczynski SF, Hendrickson CA, Harper A, Unruh TR, Dhingra A, Ahn SJ, et al. Neuropeptides and peptide hormones identified in codling moth, Cydia pomonella (Lepidoptera: Tortricidae). Arch Insect Biochem Physiol. 2019;101. doi:10.1002/ARCH.21587

31. Hartenstein V. The neuroendocrine system of invertebrates: a developmental and evolutionary perspective. J Endocrinol. 2006;190: 555–570. doi:10.1677/JOE.1.06964

32. De Haes W, Van Sinay E, Detienne G, Temmerman L, Schoofs L, Boonen K. Functional neuropeptidomics in invertebrates. Biochim Biophys Acta. 2015;1854: 812–826. doi:10.1016/J.BBAPAP.2014.12.011

33. Veenstra JA. Coleoptera genome and transcriptome sequences reveal numerous

differences in neuropeptide signaling between species. PeerJ. 2019;2019. doi:10.7717/peerj.7144

34. Verlinden H, Vleugels R, Zels S, Dillen S, Lenaerts C, Crabbé K, et al. Receptors for Neuronal or Endocrine Signalling Molecules as Potential Targets for the Control of Insect Pests. Adv In Insect Phys. 2014;46: 167–303. doi:10.1016/B978-0-12-417010-0.00003-3

35. Kahveci K, Düzgün MB, Atis AE, Yılmaz A, Shahraki A, Coskun B, et al. Discovering allatostatin type-C receptor specific agonists. Nat Commun. 2024;15. doi:10.1038/S41467-024-48156-W

36. Nässel DR, Winther ÅME. Drosophila neuropeptides in regulation of physiology and behavior. Prog Neurobiol. 2010;92: 42–104. doi:10.1016/j.pneurobio.2010.04.010

37. Han S, Chen J, Liu Z, Zhang M, Guo P, Liu X, et al. Identification and expression profiling of neuropeptides and neuropeptide receptor genes in a natural enemy, Coccinella septempunctata. Front Physiol. 2024;15. doi:10.3389/FPHYS.2024.1464989

38. Sehadova H, Takasu Y, Zaloudikova A, Lin YH, Sauman I, Sezutsu H, et al. Functional Analysis of Adipokinetic Hormone Signaling in Bombyx mori. Cells. 2020;9. doi:10.3390/CELLS9122667

39. Shen Z, Liu Z, Chen J, Li Y, Mao J, Wang M, et al. Functional analysis of adipokinetic hormone signaling in reproductive diapause of Coccinella septempunctata. Pest Manag Sci. 2024;80: 3665–3674. doi:10.1002/PS.8070

40. Gäde G. The adipokinetic hormone/red pigment-concentrating hormone peptide family: Structures, interrelationships and functions. J Insect Physiol. 1990;36: 1–12. doi:10.1016/0022-1910(90)90145-6

41. Gäde G, Auerswald L. Mode of action of neuropeptides from the adipokinetic hormone

family. Gen Comp Endocrinol. 2003;132: 10–20. doi:10.1016/S0016-6480(03)00159-X

42. Ren GR, Hauser F, Rewitz KF, Kondo S, Engelbrecht AF, Didriksen AK, et al. CCHamide-2 Is an Orexigenic Brain-Gut Peptide in Drosophila. PLoS One. 2015;10. doi:10.1371/JOURNAL.PONE.0133017

43. Zhu Z, Tsuchimoto M, Nagata S. CCHamide-2 Signaling Regulates Food Intake and Metabolism in Gryllus bimaculatus. Insects. 2022;13. doi:10.3390/INSECTS13040324

44. Xu G, Gu GX, Teng ZW, Wu SF, Huang J, Song QS, et al. Identification and expression profiles of neuropeptides and their G protein-coupled receptors in the rice stem borer Chilo suppressalis. Sci Reports 2016 61. 2016;6: 1–15. doi:10.1038/srep28976

45. Park Y, Filippov V, Gill SS, Adams ME. Deletion of the ecdysis-triggering hormone gene leads to lethal ecdysis deficiency. Development. 2002;129: 493–503. doi:10.1242/DEV.129.2.493

46. Shi Y, Liu TY, Jiang HB, Liu XQ, Dou W, Park Y, et al. The Ecdysis Triggering Hormone System, via ETH/ETHR-B, Is Essential for Successful Reproduction of a Major Pest Insect, Bactrocera dorsalis (Hendel). Front Physiol. 2019;10. doi:10.3389/FPHYS.2019.00151

47. Malhotra P, Basu S. The Intricate Role of Ecdysis Triggering Hormone Signaling in Insect Development and Reproductive Regulation. Insects. 2023;14. doi:10.3390/INSECTS14080711

48. Begum K, Li B, Beeman RW, Park Y. Functions of ion transport peptide and ion transport peptide-like in the red flour beetle Tribolium castaneum. Insect Biochem Mol Biol. 2009;39: 717–725. doi:10.1016/J.IBMB.2009.08.005

49. Wang Z, Zhou W, Hameed MS, Liu J, Zeng X. Characterization and Expression Profiling

of Neuropeptides and G-Protein-Coupled Receptors (GPCRs) for Neuropeptides in the Asian Citrus Psyllid, Diaphorina citri (Hemiptera: Psyllidae). Int J Mol Sci. 2018;19. doi:10.3390/IJMS19123912

50. Lee KS, You KH, Choo JK, Han YM, Yu K. Drosophila short neuropeptide F regulates food intake and body size. J Biol Chem. 2004;279: 50781–50789. doi:10.1074/JBC.M407842200

51. Gao H, Li Y, Zhang H, Wang S, Feng F, Tang J, et al. Comparative study of neuropeptide signaling systems in Hemiptera. Insect Sci. 2023;30: 705–724. doi:10.1111/1744-7917.13120

52. Down RE, Matthews HJ, Audsley N. Effects of Manduca sexta allatostatin and an analog on the pea aphid Acyrthosiphon pisum (Hemiptera: Aphididae) and degradation by enzymes from the aphid gut. Peptides. 2010;31: 489–497. doi:10.1016/J.PEPTIDES.2009.06.017

53. Matthews HJ, Down RE, Audsley N. Effects of Manduca sexta allatostatin and an analogue on the peach-potato aphid Myzus persicae (hemiptera: aphididae) and degradation by enzymes in the aphid gut. Arch Insect Biochem Physiol. 2010;75: 139–157. doi:10.1002/ARCH.20376

54. Audsley N, Vandersmissen HP, Weaver R, Dani P, Matthews J, Down R, et al. Characterisation and tissue distribution of the PISCF allatostatin receptor in the red flour beetle, Tribolium castaneum. Insect Biochem Mol Biol. 2013;43: 65–74. doi:10.1016/J.IBMB.2012.09.007

55. Bao C, Yang Y, Huang H, Ye H. Neuropeptides in the cerebral ganglia of the mud crab , Scylla paramamosain : transcriptomic analysis and expression profiles during

vitellogenesis. 2015; 1–15. doi:10.1038/srep17055

56. Shafer OT, Yao Z. Pigment-Dispersing Factor Signaling and Circadian Rhythms in Insect Locomotor Activity. Curr Opin insect Sci. 2014;1: 73–80. doi:10.1016/J.COIS.2014.05.002

57. Li MM, Yang Q, Chen LH, Li YY, Wu JX, Xu XL. Effect of short neuropeptide F signaling on larval feeding in Mythimna separata. Insect Sci. 2024;31: 417–434. doi:10.1111/1744-7917.13246

58. Abou El Asrar R, Cools D, Vanden Broeck J. Role of peptide hormones in insect gut physiology. Curr Opin Insect Sci. 2020;41: 71–78. doi:10.1016/J.COIS.2020.07.004

59. Kong X, Li ZX, Gao YQ, Liu FH, Chen ZZ, Tian HG, et al. Genome-Wide Identification of Neuropeptides and Their Receptors in an Aphid Endoparasitoid Wasp, Aphidius gifuensi. Insects. 2021;12. doi:10.3390/INSECTS12080745

60. Wu HP, Wang XY, Hu J, Su RR, Lu W, Zheng XL. Identification of neuropeptides and neuropeptide receptor genes in Phauda flammans (Walker). Sci Reports 2022 121. 2022;12: 1–13. doi:10.1038/s41598-022-13590-7

61. Huang C, Dong X, Yang X, Zou J, Yang M, Wang X, et al. Identification of neuropeptides and their G protein-coupled receptors in the predatory stink bug, Arma custos (Hemiptera: Pentatomidae). Arch Insect Biochem Physiol. 2024;115: e22094. doi:10.1002/ARCH.22094

62. Hou QL, Chen EH, Jiang HB, Wei DD, Gui SH, Wang JJ, et al. Adipokinetic hormone receptor gene identification and its role in triacylglycerol mobilization and sexual behavior in the oriental fruit fly (Bactrocera dorsalis). Insect Biochem Mol Biol. 2017;90: 1–13. doi:10.1016/J.IBMB.2017.09.006

63. Hou L, Jiang F, Yang P, Wang X, Kang L. Molecular characterization and expression profiles of neuropeptide precursors in the migratory locust. Insect Biochem Mol Biol. 2015;63: 63–71. doi:10.1016/j.ibmb.2015.05.014

64. Nässel DR. A brief history of insect neuropeptide and peptide hormone research. Cell Tissue Res. 2025;399. doi:10.1007/S00441-024-03936-0

65. Ons S. Neuropeptides in the regulation of Rhodnius prolixus physiology. J Insect Physiol. 2017;97: 77–92. doi:10.1016/j.jinsphys.2016.05.003

Fig 1

Click here to access/download;Figure;Fig 1.tif

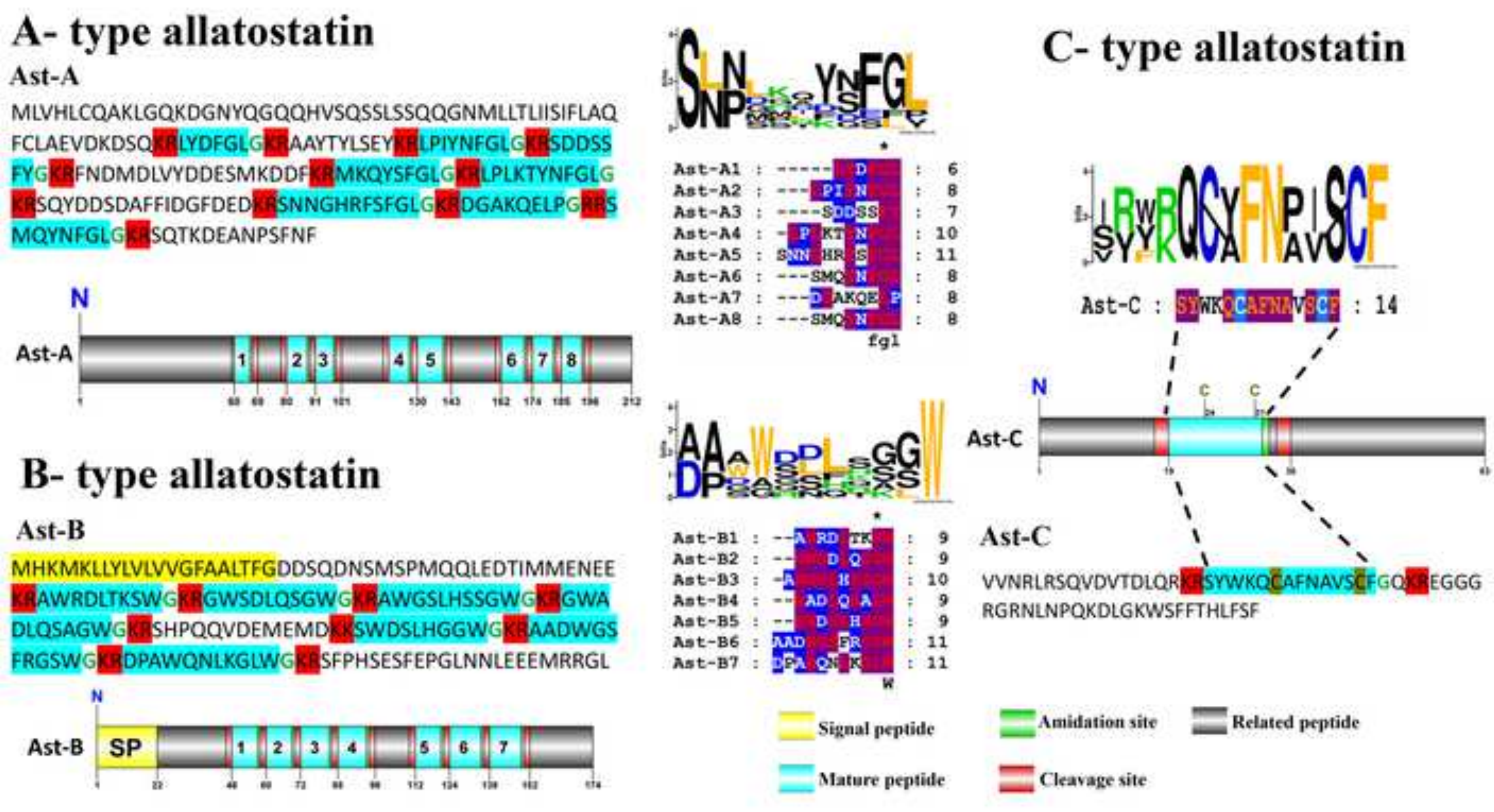

Click here to access/download;Figure;Fig 2.tif

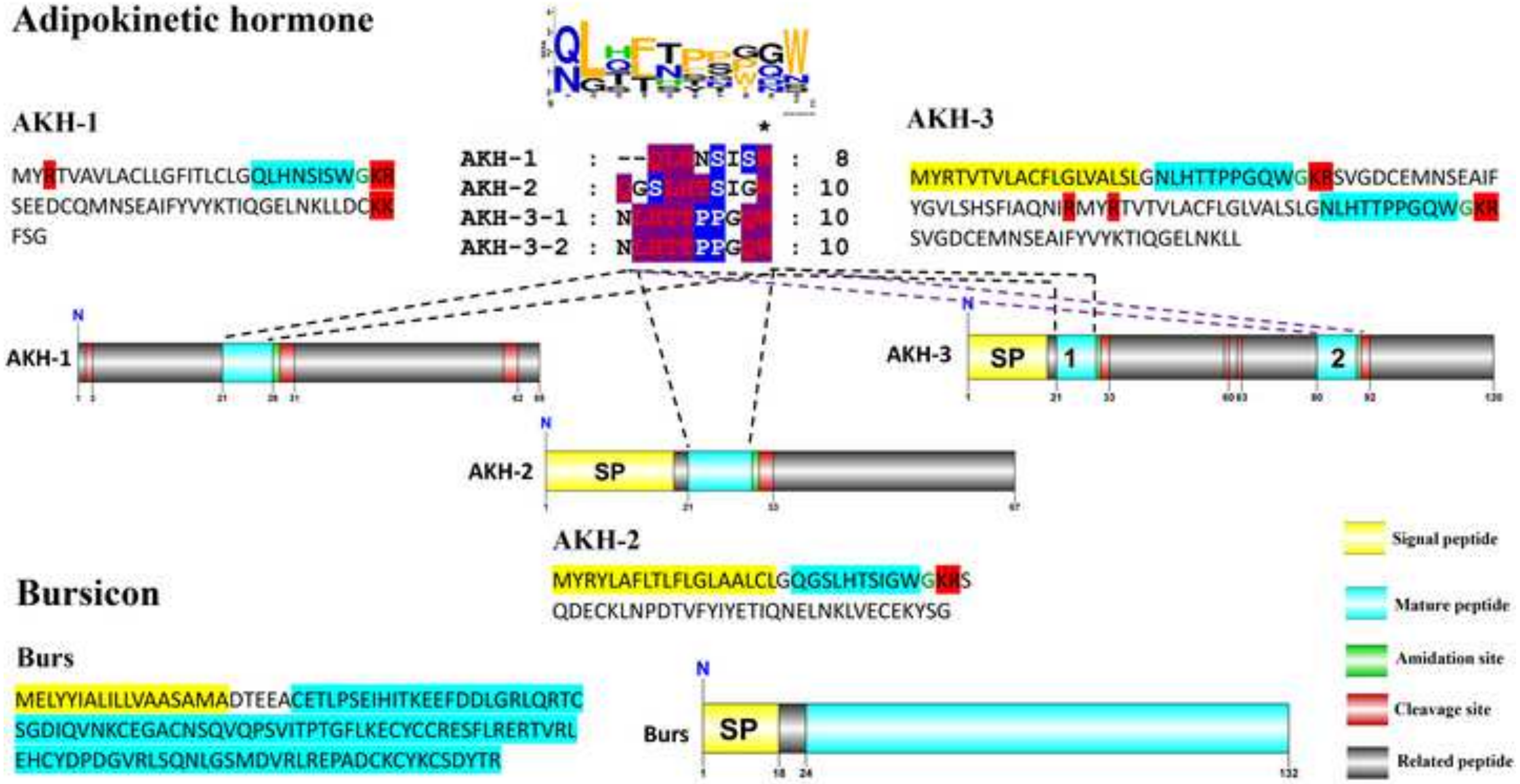

Fig 3 Click here to access/download;Figure;Fig 3.tif

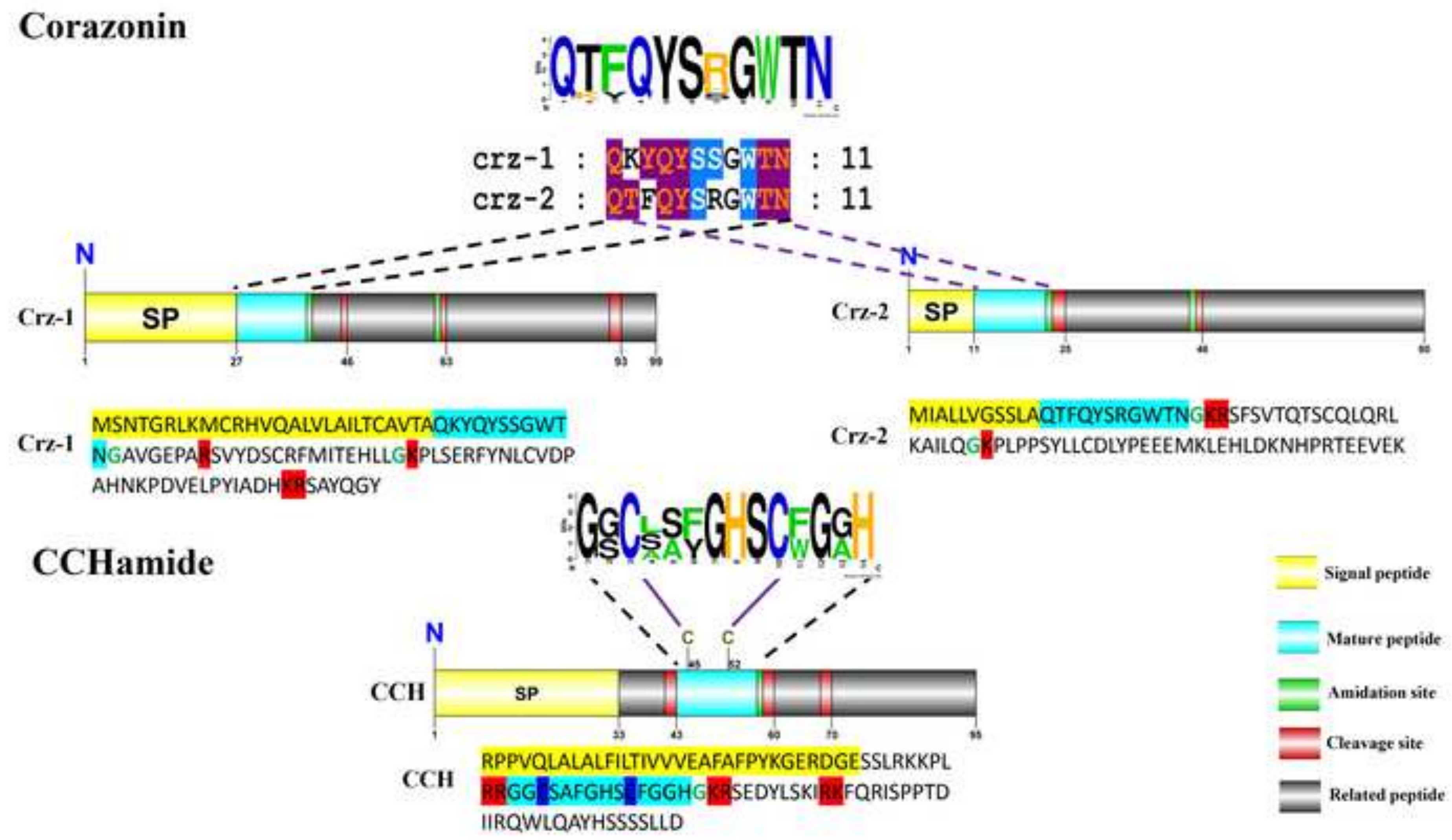

Fig 4
Click here to access/download;Figure;Fig 4.tif

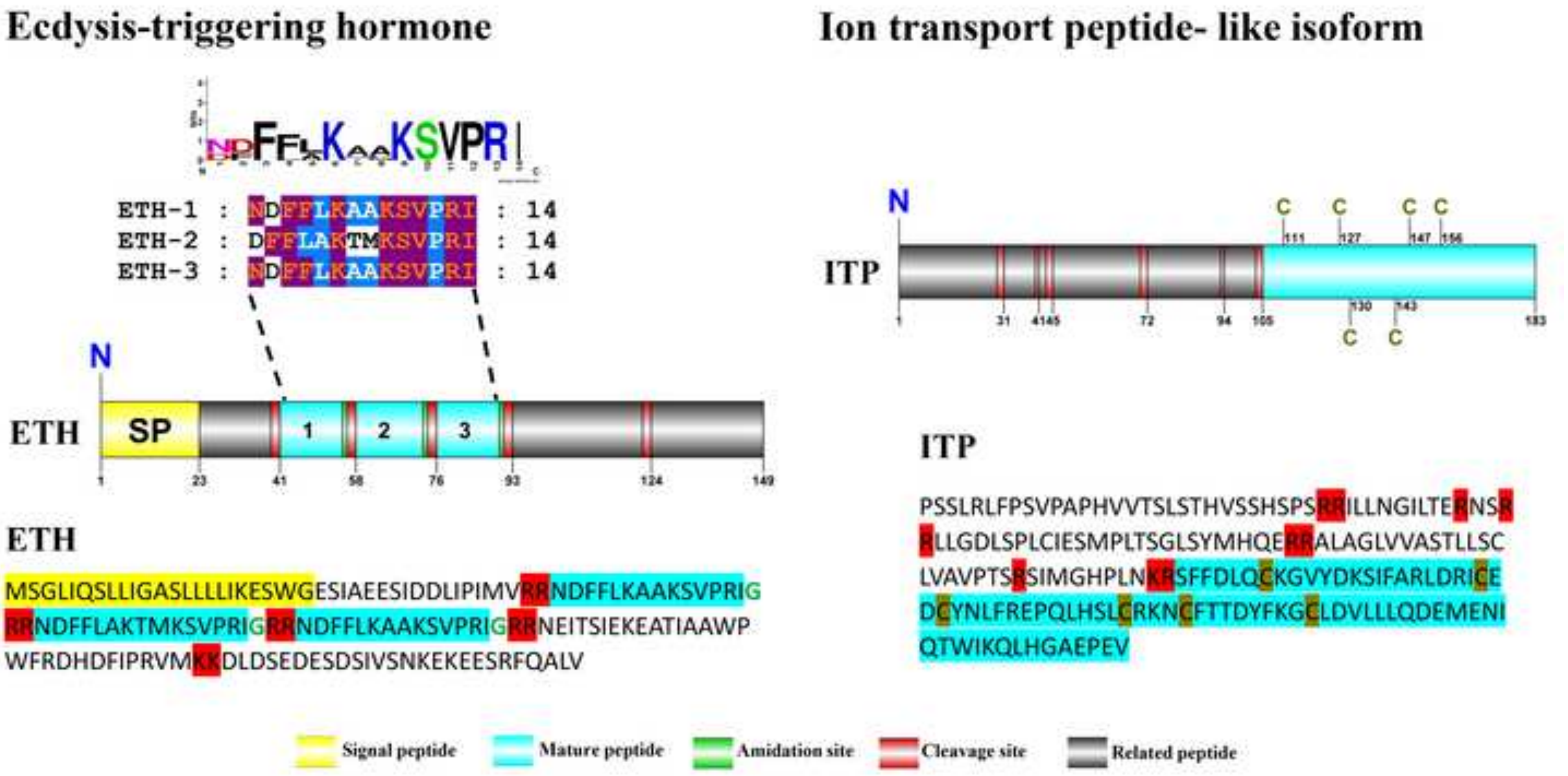

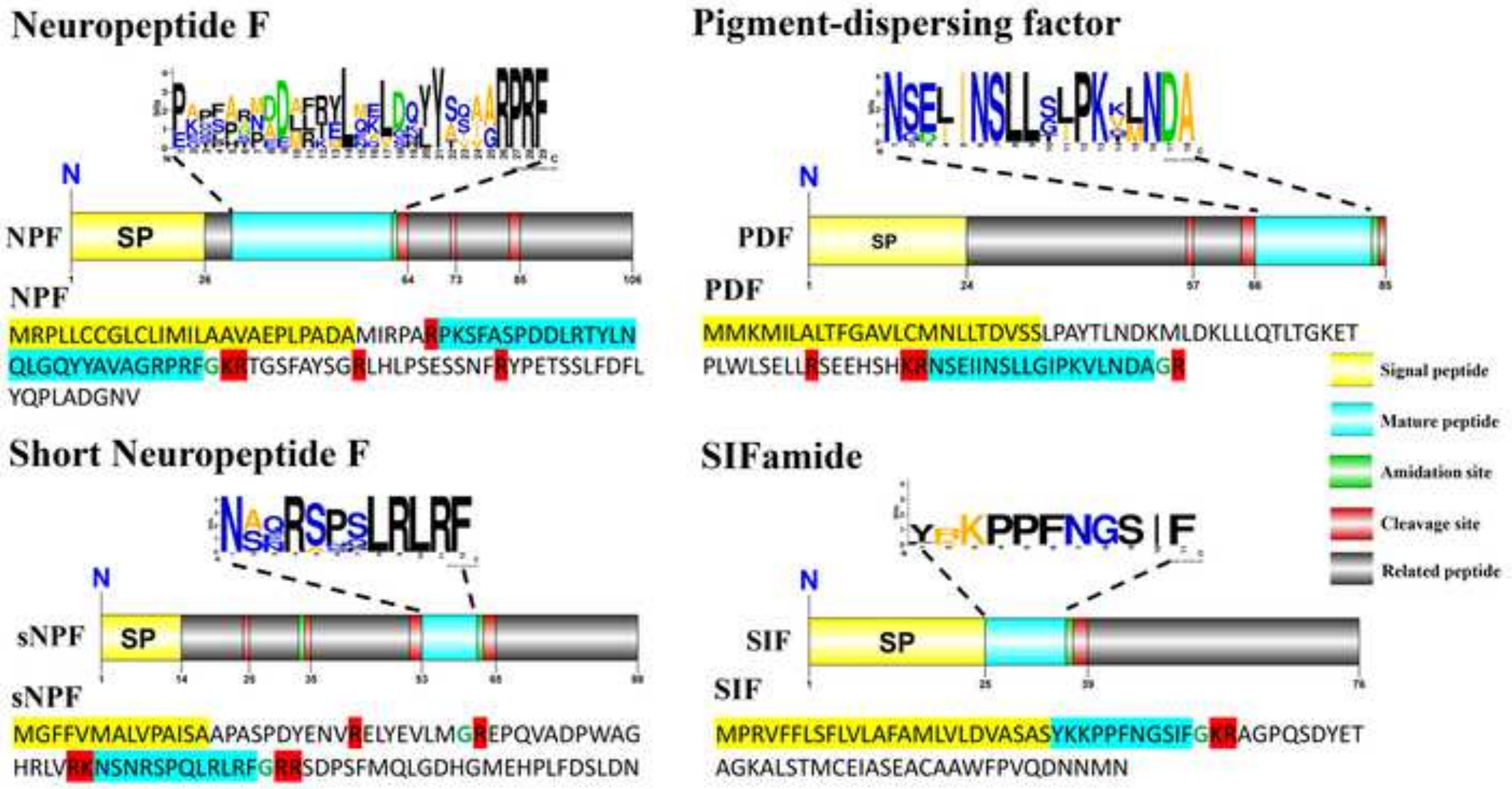
Neuropeptide F
N
NPF
SP
NPF
MRPLLCCGLCLIMILAAVAEPLPADAMIRPARPKSFASPDDLRTYLN
QLGQYYAVAGRPRFGKRTGSFAYSGRLHLPSESSNFRYPETSSLFDFL
YQPLADGNV
Pigment-dispersing factor
N
PDF
SP
PDF
MMKMILALTFGAVLCMNLLTDVSSLPAYTLNDKMLDKLLLQTLTGKET
PLWLSELLRSEEHSHKRNSEIINSLLGIPKVLNDAGR
Signal peptide
Mature peptide
Amidation site
Cleavage site
Related peptide
Short Neuropeptide F
N
sNPF
SP
sNPF
MGFFVMALVPAISAAPASPDYENVRELYEVLMGREPQVADPWAG
HRLVRKNSNRSPQLRLRFGRRSDPSFMQLGDHGMEHPLFDSLDN
SIFamide
N
SIF
SP
SIF
MPRVFFLSFLVLAFAMLVLDVASASYKKPPFNGSIFGKRAGPQSDYET
AGKALSTMCEIASEACAAWFPVQDNNMN

Click here to access/download;Figure;Fig 6 .tif

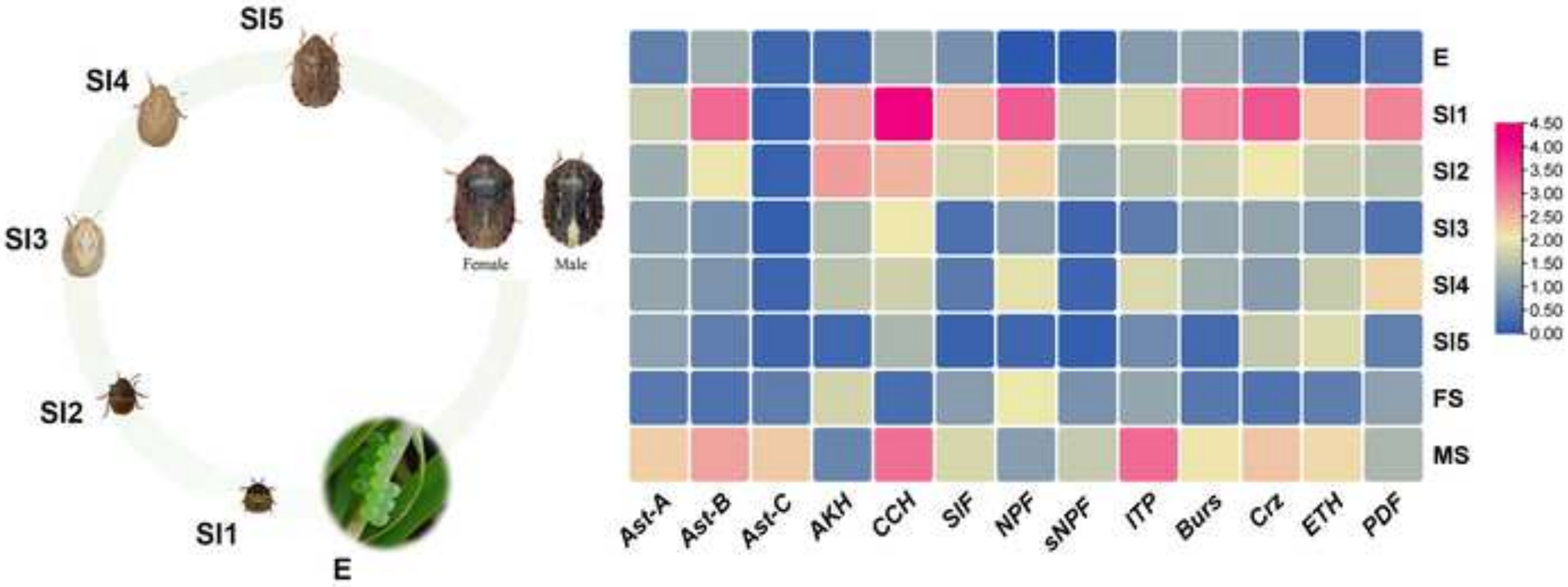

Fig 7

Click here to access/download;Figure;Fig 7.tif

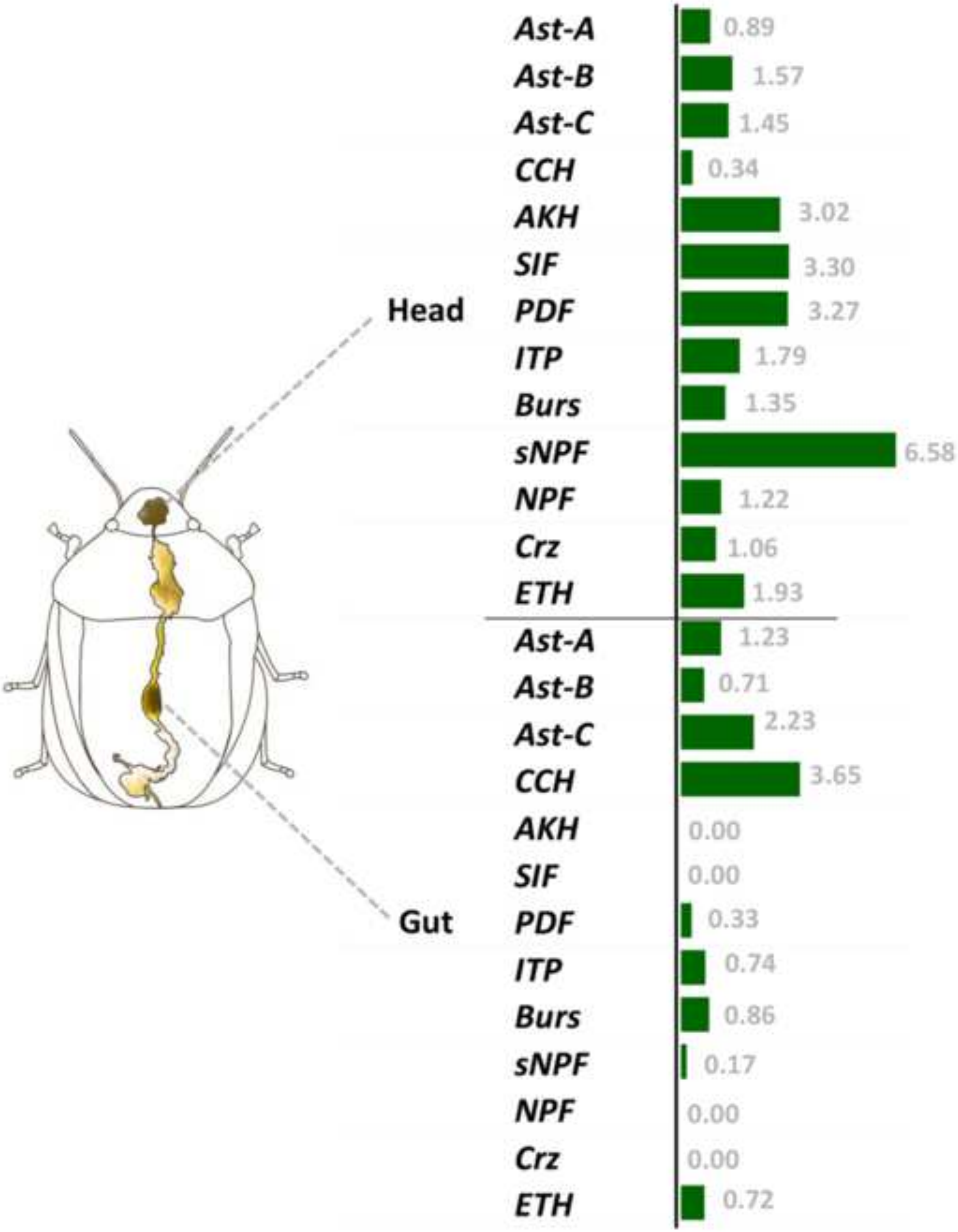

Click here to access/download
Supporting Information - Compressed/ZIP File Archive
Supporting_information.zip
