# Supplementary material for: Exploration of neuropeptides to identify potential target for regulating feeding behavior and development in Eurygaster integriceps
Source: PLoS One. 2026 Jul 17;21(7):e0353952. doi: 10.1371/journal.pone.0353952 (PMC13379102; doi:10.1371/journal.pone.0353952)
Supplement: Attachment — Submitted filename: Response to Reviewers.docx [file pone.0353952.s010.docx]

**Response to Reviewers**

**Reviewer #1: Manuscript ID: PONE-D-25-46793**

Manuscript title: Exploration of neuropeptides to identify potential target for regulating feeding behavior and development in *Eurygaster integriceps*

In this manuscript, authors performed a study of neuropeptides in *Eurygaster integriceps* Puton including sequence identification and expression profiling by means of quantitative PCR. Sequence features of precursors were analyzed in detail for 13 selected neuropeptides and their expressions were examined in various developmental stages, gender comparison and tissue preference (head vs gut). With these collected data, authors deduced and summarized their potential roles in the regulation of insect physiology. Overall, this is a continuation or companion study of their previously published results (reference 15 in this manuscript) whilst reference 15 was based on RNA-seq analysis of whole-body adult samples. All the neuropeptides described in this manuscript were identified in reference 15. This manuscript alone may stand technically sound after revisions, but the major challenge is to distinguish the scientific advancement from reference 15 in authors’ text descriptions. Reference 15 has described the potential roles of putative neuropeptides in physiological processes including stress and addiction, water balance, feeding, circadian rhythms, reproduction and development. The above-mentioned is my biggest concern for this submitted manuscript.

**Response: We thank the reviewer for raising this important point. The previously published results only showed a general transcriptomic catalog of neuropeptide precursors from adult whole-body. In contrast, the current work represents a distinct and substantially expanded novel investigation through the following analyses:**

- **Targeted selection and structural validation of neuropeptides linked to feeding regulation.**
- **Developmental expression profiling across six life stages.**
- **Tissue-specific analysis of head vs. gut.**
- **Sex-specific comparison between male and female adults.**
- **Quantitative validation, which was not performed previously.**

**Further, we have also revised the Materials and Methods (Line 117 of revised version of manuscript) to include a new subsection (“Transcriptome data mining and peptide structural analysis”), clearly explaining how the earlier RNA-seq dataset was re-analyzed and expanded for this functional study.**

**Reviewer #1-1**: Line 50, 46 neuropeptides were not identified in this manuscript. It only described 13 selected ones from reference 15.

**Response#1-1: We agree that this manuscript does not identify 46 neuropeptides. Those 46 precursors were identified only in our previous RNA-seq study (Ref. 16). The present work specifically examines 13 neuropeptides selected for their documented roles in feeding regulation. To avoid confusion, we have revised the text to clearly reflect this focus. The updated sentence (Lines 46-50 of revised version of manuscript) now reads:**

**“In this study, we focused on 13 feeding-related neuropeptides previously identified from our whole-body RNA-seq dataset of *Eurygaster integriceps*. Using a combination of bioinformatics and expression analyses, we characterized precursor structures, predicted mature peptides, and quantified expression across developmental stages, between sexes, and in key feeding-associated organs of head and gut.”**

**Reviewer #1-2: Statistical analysis in materials and methods. How pair-wise analysis (Fig. S2 and S3) was performed is not described in materials and methods**.

**Response#1-2: We have now clarified the statistical procedures used for the pair-wise analyses shown in S2 Fig and S3 Fig. A detailed description of the methods— including the use of independent-samples t-tests for two-group comparisons and one-way ANOVA with Tukey’s HSD for multi-group comparisons—has been added to the Statistical Analysis section at Lines 185–192 of the revised manuscript.**

**Reviewer #1-3**: **Table S2 is a shortened form of Table 2 from reference 15. This is a duplicate publication of the same results.**

**Response#1-3:** **Thank you for the comment. Table S2 is not a shortened or duplicated form of Table 2 from Reference 16. Reference 16 was a general transcriptome survey that catalogued all predicted neuropeptide precursors in *E. integriceps*, but it did not include differential expression analyses, sex-specific comparisons, tissue-specific comparisons, or developmental profiling. In the present study, we focus exclusively on 13 neuropeptides that showed functional relevance to feeding and development based on our new expression analyses. Table S2 includes only these 13 sequences so that readers can easily reference the specific precursors examined in the qPCR experiments. Therefore, Table S2 is a study-specific subset created to support the new analyses presented here, and it does not duplicate the content of Table 2 in Reference 16.**

**Reviewer #1- 4-1. Comparing Table S2 (this manuscript) and Table 2 (reference 15), it is confusing that sequence lengths and signal peptide lengths are different for the following precursors, AKH1, AKH2, AKH3, Burs, Crz-2, ETH, NPF, sNPF and SIF. They have the same lists of gene IDs and homology analysis in two manuscripts.**

**Response#1-4-1: Thank you for raising this important point. We agree that several precursors (AKH1, AKH2, AKH3, Burs, Crz-2, ETH, NPF, sNPF, and SIF) show differences in sequence length and signal peptide length between Table S2 of the current manuscript and Table 2 of Reference 16, despite sharing the same gene IDs. These differences do not represent conflicting results but rather updated and more accurate annotations. In Reference 16, neuropeptide precursors were reported as part of a general transcriptome survey, and the ORF predictions were generated using automated pipelines that allowed multiple potential start codons. For the present study, we re-evaluated all 13 selected neuropeptide genes manually, applying a stricter annotation approach that uses the canonical methionine (ATG) start codon and validates signal peptide regions using dedicated prediction tools. This refinement was led to adjustments in the ORF boundaries for some precursors, which in turn produced updated total and signal peptide lengths. Thus, the sequences presented in Table S2 represent corrected and biologically refined precursor annotations, tailored specifically for the structural and expression analyses performed in this study. We have added a clarification in the revised manuscript to prevent confusion between the broad transcriptome-based predictions in Reference 16 and the updated, manually curated precursor sequences used here.**

**Reviewer #1-4-2: Also, why was Ast-C2 omitted for analysis in this study for the Ast family?**

**Response#1-4-2:** **In the present study, we selected only one Ast-C precursor for experimental validation because the two predicted Ast-C transcripts share highly similar precursor architectures and mature peptide regions. Therefore, including both would not have provided additional biological insight into this neuropeptide family. We prioritized a single representative Ast-C precursor for qPCR analysis to avoid redundancy and to focus resources on neuropeptides with distinct sequences and predicted functions. This clarification has now been added to the revised manuscript.**

**Reviewer #1-5: Fig. 6 and Fig. 7 look nice but are better replaced by Fig. S1, S2 and S3. Result description by means of Fig. 6 and Fig. 7 largely disregarded the statistical analyses in S1, S2 and S3**

**Response#1-5:** **We thank the reviewer for this helpful comment. Figures 6 and 7 serve as visual summaries, and the detailed statistical analyses validating these patterns are now explicitly referenced in Supplementary Figures S1–S3. We have added citations to these supplementary figures in Lines 304–305, 322–325, and 349–351 to clearly direct readers to the corresponding statistical support.**

**Reviewer #2: This manuscript presents an investigation into the neuropeptidome of the Sunn pest (*Eurygaster integriceps*), combining transcriptome mining, bioinformatics prediction, and RT-qPCR expression profiling across developmental stages, sexes, and tissues. The topic is relevant, given the interest in species-specific molecular targets for sustainable pest management.**

**However, while the research is promising, the manuscript would benefit from some rework in its presentation form. Several claims are descriptive, while the statistics are present in the supplement; bringing some of the information from the external file to the main text would benefit the readability and interpretation.**

**Response#2:** **We appreciate the reviewer’s insightful suggestion. To improve readability and strengthen the integration between descriptive results and statistical evidence, we have added explicit references to the supporting statistical analyses in Supplementary Figures S1–S3 within the Results section (Lines 304–305, 322–325, and 349–351). In addition, we incorporated clearer interpretation of stage-specific expression patterns into the Discussion (Lines 433–441), including examples such as the developmental fluctuations in PDF, ITP, and Allatostatin expression.**

**Reviewer #2-1: Specify the accession numbers for the RNA-seq dataset used.**

**Response#2-1: BioProject accession number PRJNA1015108 has now been stated in the manuscript (Line 120).**

**Reviewer #2-2: Provide software versions, parameter settings, and statistical methods used in analyses.**

**Response#2-2: The software tools and their versions (e.g., Oligo7 v7.60, Primer3 v2.6.1, ClustalX 2.1, WebLogo 2.8.2) are now specified in the Materials and Methods (Lines 131–132 and 168). Parameter settings and statistical methods have also been added, including one-way ANOVA with Tukey’s HSD (P < 0.05), as outlined in Lines 190–192, which now read: “For comparisons involving more than two groups (developmental stages), one-way ANOVA was conducted, followed by Tukey’s HSD post hoc test. Statistical significance was set at *P* < 0.05.”**

**Reviewer #2-3 Identify the reference genes used and provide validation data for their stability, and add a validation test of these gene across different developmental stages to the supplement.**

**Response#2-3: We appreciate the reviewer’s suggestion. Two reference genes (Actin and 18S rRNA) were evaluated for stability, and 18S rRNA showed the most consistent expression across developmental stages. The stability assessment—including mean, SD, SEM, and CV%—is now described in the Methods (Lines 175–181) and further supported in the Results (Lines 322–325). Complete validation data are provided in Supplementary Fig. S2 and Supplementary Table S4.**

**Reviewer #2-4 The Results are descriptive. Claims of higher or lower expression would benefit by the support of quantitative fold-change values**

**Response#2-4: We thank the reviewer for this constructive comment. Quantitative fold-change values have been added to support the reported expression patterns. The text now reads (Lines 433–441):**

**For Neuropeptide PDF, expression decreased to 0.3-fold in SI3 and subsequently increased to 3.7-fold in SI4. Similarly, Neuropeptide ITP showed a reduction to 0.4-fold in SI3 followed by an increase to 2.5-fold in SI4. These patterns suggest that neuropeptides may have stage-specific regulatory functions in E. integriceps. For example, Allatostatins Ast-A and Ast-B peaked during the first instar, with 2.1-fold and 8.1-fold increases relative to eggs, respectively, whereas Ast-C exhibited its highest expression in eggs. During nymphal stages, Ast-C expression was lower than in eggs, with fold-changes of 0.5, 0.3, 0.2, 0.4, and 0.2 in the first to fifth instars, respectively (S1 Fig), suggesting its role in early embryonic development rather than feeding regulation.**

**Reviewer #2-5 Indicate how normalization was performed (ΔΔCt relative to which calibrator stage or tissue).**

**Response#2-5**: **The normalization procedure (ΔΔCt method and the calibrators used) has now been fully clarified in the Materials and Methods. The text now reads (Lines 186–192):**

**"** **The relative gene expression levels (ΔCq values normalized to reference genes) were calculated for each sample, and results are presented as mean ± standard error (SE). Pairwise comparisons between two groups (male vs. female, and head vs. gut) were performed using independent-samples t-tests. For comparisons involving more than two groups (developmental stages), one-way analysis of variance (ANOVA) was conducted, followed by Tukey’s honestly significant difference (HSD) post hoc test. Statistical significance was set at P < 0.05. All analyses were performed using SPSS version 27.0 (IBM Corp., Armonk, NY, USA). "**

**Reviewer #2-6 Figures and tables in the supplementary material are essential for interpreting results. These could be partially summarized and moved into the main manuscript.**

**Response#2-6: We have revised the Results section to include concise summaries and direct references to the relevant supplementary figures and tables, ensuring clearer linkage between the main figures and the supporting statistical analyses.**

**Lines 322–325: A summary of reference gene stability, with explicit reference to Supplementary Fig. S2 and Supplementary Table S4.**

**Lines 348–351: A description of the organ-specific expression patterns shown in Fig. 7, with a direct reference to the supporting analyses in Supplementary Fig. S3.**

**Reviewer #3 reviewer comments from the PDF file**

**Reviewer #3: The reviewer suggested adding the phrase “In this study, we analyzed previously generated RNA-seq datasets” to Lines 48–49 (reviewer comment on pdf file).**

**Response: We appreciate this useful suggestion. We have revised the manuscript accordingly. The updated text now reads (Lines 46–48):**

**"** **In this study, we focused on 13 feeding-related neuropeptides previously identified from our whole-body RNA-seq dataset of Eurygaster integriceps. "**

**Reviewer #3: The reviewer requested adding brief quantitative indicators in Line 53 (reviewer comment on pdf file).**

**Response: This revision has been completed and incorporated into the manuscript (Line 55 of the revised version).**

**Reviewer #3: The reviewer noted that the Introduction lacks a clearly defined knowledge gap specific to E. integriceps. The reviewer also requested explicit acknowledgement that this study builds on a previous transcriptomic dataset, with clarification of what new analyses and insights are provided here (reviewer comment on pdf file).**

**Response: We appreciate the reviewer’s comment. In the revised manuscript (Introduction, Lines 105–111), we clarified the specific knowledge gap and explained how this study builds on our previously generated RNA-seq dataset. Specifically, the revised text now reads (Lines 105–111):**

**"** **Our earlier whole-body RNA-seq study [16] identified 46 neuropeptide precursors in Eurygaster integriceps, but it did not resolve developmental, sex-specific, or tissue-specific expression patterns. Building on this foundational dataset, the present study focuses on major feeding-related neuropeptides for detailed structural and functional analysis. We examined precursor architecture, predicted mature peptide structures, and quantified expression across developmental stages, between sexes, and in key feeding-associated tissues (head and gut) using gene expression profiling."**

**Moreover, we also specified in the Materials and Methods that the original transcriptomic data are publicly available in the SRA: Lines 118–120: "** **The transcriptomic resources used for peptide discovery and structural characterization in this study were derived from our previously published whole-body RNA-seq dataset of E. integriceps [16], available under BioProject accession PRJNA1015108"**

**Reviewer #3: The reviewer suggested adding information on current management practices and associated challenges (reviewer comment on pdf file(**

**Response: This revision has been completed in the revised manuscript (Lines 72–78). We added relevant details and cited a recent reference:**

**Duman, M., & Alaserhat, İ. (2025). Assessment of Eurygaster integriceps parameters to improve integrated pest management in Southeastern Türkiye wheat fields. Scientific Reports, 15(1), 26031.**

**Reviewer #3: The reviewer suggested improving the link to better explain what was done previously and what is new in the current study (reviewer comment on pdf file).**

**Response: We have revised the text accordingly in the manuscript (Lines 105–114). The updated paragraph now reads:**

**"** **Our earlier whole-body RNA-seq study [16] identified 46 neuropeptide precursors in Eurygaster integriceps, but it did not resolve developmental, sex-specific, or tissue-specific expression patterns. Building on this foundational dataset, the present study focuses on major feeding-related neuropeptides for detailed structural and functional analysis. We examined precursor architecture, predicted mature peptide structures, and quantified expression across developmental stages, between sexes, and in key feeding-associated tissues (head and gut) using gene expression profiling. By integrating precursor structure with validated expression patterns, this research delivers the first comprehensive expression profile of feeding-related neuropeptides in E. integriceps and provides new molecular insight into the neuropeptidergic network underlying feeding behavior and development. "**

**Reviewer #3: The reviewer requested specifying the accession numbers for the RNA-seq dataset used (reviewer comment on pdf file).**

**Response: We have revised the manuscript accordingly (Lines 118–120). The updated text now reads:**

**“ The transcriptomic resources used for peptide discovery and structural characterization in this study were derived from our previously published whole-body RNA-seq dataset of E. integriceps [16], available under BioProject accession PRJNA1015108. ”**
