# Supplementary material for: Exploration of neuropeptides to identify potential target for regulating feeding behavior and development in Eurygaster integriceps
Source: PLoS One. 2026 Jul 17;21(7):e0353952. doi: 10.1371/journal.pone.0353952 (PMC13379102; doi:10.1371/journal.pone.0353952)
Supplement: Attachment — Submitted filename: Response_to_Reviewers_auresp_2.docx [file pone.0353952.s011.docx]

**Response to Reviewers**

PONE-D-25-46793R2

Reviewer #1-1: Double-check all the gene expression interpretations across the text.

Response #1-1: We appreciate the reviewer’s suggestion. Double-check and minor corrections have carefully been done all the gene expression interpretations across the text.

Reviewer #1-2: The Abstract states that several neuropeptides show higher expression in females. In the Results section, it is stated that these same neuropeptides show significantly higher expression in males, with only CCH and PDF being higher in females.

Response #1-2: We thank the reviewer for raising this mistake in the Abstract section. The expression level of *Ast-A*, *Ast-B*, *Ast-C*, *AKH*, *SIF*, *ITP*, *Burs*, *sNPF*, *NPF*, and *Crz*, were higher in males than in females, and the statement is correct as already mentioned in the Results section (according to S2 Fig). Now, we have corrected the writing mistake in the Abstract section (Revised version of manuscript, Lines 50-53). The text now it reads:

"Several neuropeptides, including *Ast-A*, *Ast-B*, *Ast-C*, *AKH*, *SIF*, *ITP*, *Burs*, *sNPF*, *NPF*, and Crz showed higher expression in males than in females, suggesting sexually dimorphic regulation of feeding and metabolism."

Reviewer #1-3: Several statements in the Results and Discussion imply functional roles. Please review these statements to show correlation or causation supported by evidence.

Response #1-3: We have added more references in the revised version of manuscript to show correlation or causation supported by evidence (Discussion section, Lines 484-486 and 506-507).

Reference [59]: Yao H, et al. The neuropeptide pigment-dispersing factor enhanced food intake and alleviated antifeedant effects by azadirachtin in Spodoptera frugiperda larvae. 2026;119: 1402–1411.

Reference [65]: Garelli A, et al. Body growth and shape regulation by neuropeptides and other neuroactive peptides in insects. Insect Sci. 2026;76: 1–9.

Reviewer #1-4: The Methods describe different numbers of individuals per developmental stage, but do not explicitly state whether samples were pooled prior to RNA extraction and qPCR. Please clarify.

Response #1-4: We thank the reviewer for this helpful comment. Now, we have explicitly stated in the revised version of manuscript. The revised text now it reads:

Lines 148-152 of revised manuscript: "For each stage we prepared a single pooled sample that comprised more than 100 eggs, 30 individuals for each of the 1st to 3rd instar nymphs, and 10 individuals for each of the 4th and 5th instar nymphs, female adults, and male adults. Samples were immediately frozen in liquid nitrogen and stored at −80 °C until further experiments."

"Line 158 of revised manuscript: "The pooled organs were pulverized in liquid nitrogen and stored at −80 °C prior to RNA extraction."
