# Supplementary material for: Exploration of neuropeptides to identify potential target for regulating feeding behavior and development in Eurygaster integriceps
Source: PLoS One. 2026 Jul 17;21(7):e0353952. doi: 10.1371/journal.pone.0353952 (PMC13379102; doi:10.1371/journal.pone.0353952)
Supplement: Attachment — Submitted filename: Response to Reviewer.docx [file pone.0353952.s012.docx]

**Response to Reviewers**

PONE-D-25-46793R3

Reviewer #2: The authors have satisfactorily addressed the major scientific concerns raised during the previous review rounds. The manuscript now provides a coherent description of neuropeptide precursor structures and expression patterns across developmental stages, sexes, and tissues in Eurygaster integriceps. The study is primarily descriptive in nature and represents a valuable molecular resource for future investigations of neuropeptide function in this economically important pest species.

I have one remaining concern regarding the description of biological replication in the RT-qPCR experiments. In the Methods section, the authors indicate that pooled samples were prepared for each developmental stage, while elsewhere they state that three biological replicates were used. The wording could be interpreted as suggesting that only a single pooled sample was generated per stage, which would not support the inferential statistical analyses reported. My interpretation, however, is that the authors likely generated multiple biological replicates using pooled individuals and that the issue is primarily one of methodological description rather than a fundamental flaw in the experimental design. Please clarify

Response #2-1: We sincerely thank the reviewer for the careful evaluation of our manuscript and for the positive comments on the revisions. We appreciate the reviewer’s important observation regarding the description of biological replication in the RT-qPCR experiments. To clarify, for each developmental stage, sex, and tissue type, we prepared three independent biological replicates, each consisting of a separate pooled sample of multiple individuals. Therefore, pooled sampling was used within each replicate to obtain sufficient RNA and reduce individual variability, but replication was maintained through independently prepared pools. This ensures that the statistical analyses performed are supported by true biological replication. We have revised the Methods section accordingly. The revised text now reads (Lines 148-156 of the revised version of the manuscript):

"For each stage three independent biological replicates were prepared. Each biological replicate consisted of a separate pooled sample of multiple individuals, including more than 100 eggs, 30 individuals for each of the 1st to 3rd instar nymphs, and 10 individuals for each of the 4th and 5th instar nymphs, as well as female and male adults. Samples were immediately frozen in liquid nitrogen and stored at −80 °C until further experiments.

For organ-specific analysis, female and male adults were dissected to isolate heads and guts. For each tissue type, three independent biological replicates were prepared, each consisting of pooled samples (15 heads or 15 guts per replicate)."
