# Supplementary material for: Exploration of neuropeptides to identify potential target for regulating feeding behavior and development in Eurygaster integriceps
Source: PLoS One. 2026 Jul 17;21(7):e0353952. doi: 10.1371/journal.pone.0353952 (PMC13379102; doi:10.1371/journal.pone.0353952)
Supplement: Peer review history

# Peer review: Decision Letter 0

**DOI:** 10.1371/journal.pone.0353952.r001

**Authors:** Patrizia Falabella

4 Nov 2025

PONE-D-25-46793

Exploration of neuropeptides to identify potential target for regulating feeding behavior and development in Eurygaster integriceps

PLOS ONE

Dear Dr. Ghaffari,

Thank you for submitting your manuscript to PLOS ONE. After careful consideration, we feel that it has merit but does not fully meet PLOS ONE’s publication criteria as it currently stands. Therefore, we invite you to submit a revised version of the manuscript that addresses the points raised during the review process.

Please submit your revised manuscript by Dec 19 2025 11:59PM. If you will need more time than this to complete your revisions, please reply to this message or contact the journal office at plosone@plos.org. When you're ready to submit your revision, log on to https://www.editorialmanager.com/pone/ and select the 'Submissions Needing Revision' folder to locate your manuscript file.

Please include the following items when submitting your revised manuscript:

- A rebuttal letter that responds to each point raised by the academic editor and reviewer(s). You should upload this letter as a separate file labeled 'Response to Reviewers'.
- A marked-up copy of your manuscript that highlights changes made to the original version. You should upload this as a separate file labeled 'Revised Manuscript with Track Changes'.
- An unmarked version of your revised paper without tracked changes. You should upload this as a separate file labeled 'Manuscript'.

If you would like to make changes to your financial disclosure, please include your updated statement in your cover letter. Guidelines for resubmitting your figure files are available below the reviewer comments at the end of this letter.

If applicable, we recommend that you deposit your laboratory protocols in protocols.io to enhance the reproducibility of your results. Protocols.io assigns your protocol its own identifier (DOI) so that it can be cited independently in the future. For instructions see: https://journals.plos.org/plosone/s/submission-guidelines#loc-laboratory-protocols. Additionally, PLOS ONE offers an option for publishing peer-reviewed Lab Protocol articles, which describe protocols hosted on protocols.io. Read more information on sharing protocols at https://plos.org/protocols?utm_medium=editorial-email&utm_source=authorletters&utm_campaign=protocols.

We look forward to receiving your revised manuscript.

Kind regards,

Patrizia Falabella

Academic Editor

PLOS ONE

Journal Requirements:

When submitting your revision, we need you to address these additional requirements.

1. Please ensure that your manuscript meets PLOS ONE's style requirements, including those for file naming. The PLOS ONE style templates can be found at

https://journals.plos.org/plosone/s/file?id=wjVg/PLOSOne_formatting_sample_main_body.pdf and

https://journals.plos.org/plosone/s/file?id=ba62/PLOSOne_formatting_sample_title_authors_affiliations.pdf.

2. In your Methods section, please provide additional information regarding the permits you obtained for the work. Please ensure you have included the full name of the authority that approved the field site access and, if no permits were required, a brief statement explaining why.

3. PLOS requires an ORCID iD for the corresponding author in Editorial Manager on papers submitted after December 6th, 2016. Please ensure that you have an ORCID iD and that it is validated in Editorial Manager. To do this, go to ‘Update my Information’ (in the upper left-hand corner of the main menu), and click on the Fetch/Validate link next to the ORCID field. This will take you to the ORCID site and allow you to create a new iD or authenticate a pre-existing iD in Editorial Manager.

4. If the reviewer comments include a recommendation to cite specific previously published works, please review and evaluate these publications to determine whether they are relevant and should be cited. There is no requirement to cite these works unless the editor has indicated otherwise.

[Note: HTML markup is below. Please do not edit.]

Reviewers' comments:

Reviewer's Responses to Questions

**Comments to the Author**

1. Is the manuscript technically sound, and do the data support the conclusions?

The manuscript must describe a technically sound piece of scientific research with data that supports the conclusions. Experiments must have been conducted rigorously, with appropriate controls, replication, and sample sizes. The conclusions must be drawn appropriately based on the data presented.

Reviewer #1: Partly

Reviewer #2: Partly

**********

2. Has the statistical analysis been performed appropriately and rigorously?

Reviewer #1: Yes

Reviewer #2: Yes

**********

3. Have the authors made all data underlying the findings in their manuscript fully available?

The PLOS Data policy requires authors to make all data underlying the findings described in their manuscript fully available without restriction, with rare exception (please refer to the Data Availability Statement in the manuscript PDF file). The data should be provided as part of the manuscript or its supporting information, or deposited to a public repository. For example, in addition to summary statistics, the data points behind means, medians and variance measures should be available. If there are restrictions on publicly sharing data—e.g. participant privacy or use of data from a third party—those must be specified.

Reviewer #1: Yes

Reviewer #2: Yes

**********

4. Is the manuscript presented in an intelligible fashion and written in standard English?

PLOS ONE does not copyedit accepted manuscripts, so the language in submitted articles must be clear, correct, and unambiguous. Any typographical or grammatical errors should be corrected at revision, so please note any specific errors here.

Reviewer #1: Yes

Reviewer #2: Yes

**********

5. Review Comments to the Author

Please use the space provided to explain your answers to the questions above. You may also include additional comments for the author, including concerns about dual publication, research ethics, or publication ethics. (Please upload your review as an attachment if it exceeds 20,000 characters)

Reviewer #1: Manuscript ID: PONE-D-25-46793

Manuscript title: Exploration of neuropeptides to identify potential target for regulating feeding behavior and development in Eurygaster integriceps

In this manuscript, authors performed a study of neuropeptides in Eurygaster integriceps Puton including sequence identification and expression profiling by means of quantitative PCR. Sequence features of precursors were analyzed in detail for 13 selected neuropeptides and their expressions were examined in various developmental stages, gender comparison and tissue preference (head vs gut). With these collected data, authors deduced and summarized their potential roles in the regulation of insect physiology.

Overall, this is a continuation or companion study of their previously published results (reference 15 in this manuscript) whilst reference 15 was based on RNA-seq analysis of whole-body adult samples. All the neuropeptides described in this manuscript were identified in reference 15. This manuscript alone may stand technically sound after revisions, but the major challenge is to distinguish the scientific advancement from reference 15 in authors’ text descriptions. Reference 15 has described the potential roles of putative neuropeptides in physiological processes including stress and addiction, water balance, feeding, circadian rhythms, reproduction and development. The above-mentioned is my biggest concern for this submitted manuscript.

Additional concerns are listed below:

1. Line 50, 46 neuropeptides were not identified in this manuscript. It only described 13 selected ones from reference 15.

2. Statistical analysis in materials and methods. How pair-wise analysis (Fig. S2 and S3) was performed is not described in materials and methods.

3. Table S2 is a shortened form of Table 2 from reference 15. This is a duplicate publication of the same results.

4. Comparing Table S2 (this manuscript) and Table 2 (reference 15), it is confusing that sequence lengths and signal peptide lengths are different for the following precursors, AKH1, AKH2, AKH3, Burs, Crz-2, ETH, NPF, sNPF and SIF. They have the same lists of gene IDs and homology analysis in two manuscripts.

Also, why was Ast-C2 omitted for analysis in this study for the Ast family?

5. Fig. 6 and Fig. 7 look nice but are better replaced by Fig. S1, S2 and S3. Result description by means of Fig. 6 and Fig. 7 largely disregarded the statistical analyses in S1, S2 and S3.

Reviewer #2: This manuscript presents an investigation into the neuropeptidome of the Sunn pest (Eurygaster integriceps), combining transcriptome mining, bioinformatics prediction, and qRT-PCR expression profiling across developmental stages, sexes, and tissues. The topic is relevant, given the interest in species-specific molecular targets for sustainable pest management.

However, while the research is promising, the manuscript would benefit from some rework in its presentation form. Several claims are descriptive, while the statistics are present in the supplement; bringing some of the information from the external file to the main text would benefit the readability and interpretation.

Other points:

Specify the accession numbers for the RNA-seq dataset used.

Provide software versions, parameter settings, and statistical methods used in analyses.

Identify the reference genes used and provide validation data for their stability, and add a validation test of these gene across different developmental stages to the supplement.

The Results are descriptive. Claims of higher or lower expression would benefit by the support of quantitative fold-change values.

Indicate how normalization was performed (ΔΔCt relative to which calibrator stage or tissue).

Figures and tables in the supplementary material are essential for interpreting results. These could be partially summarized and moved into the main manuscript.

**********

6. PLOS authors have the option to publish the peer review history of their article (what does this mean?). If published, this will include your full peer review and any attached files.

If you choose “no”, your identity will remain anonymous but your review may still be made public.

**Do you want your identity to be public for this peer review?**  For information about this choice, including consent withdrawal, please see our Privacy Policy.

Reviewer #1: No

Reviewer #2: No

**********

[NOTE: If reviewer comments were submitted as an attachment file, they will be attached to this email and accessible via the submission site. Please log into your account, locate the manuscript record, and check for the action link "View Attachments". If this link does not appear, there are no attachment files.]

To ensure your figures meet our technical requirements, please review our figure guidelines: https://journals.plos.org/plosone/s/figures

You may also use PLOS’s free figure tool, NAAS, to help you prepare publication quality figures: https://journals.plos.org/plosone/s/figures#loc-tools-for-figure-preparation.

NAAS will assess whether your figures meet our technical requirements by comparing each figure against our figure specifications.

Attachment Submitted filename: PONE-D-25-46793_reviewer.pdf

# Author comment: Author response to Decision Letter 1

**DOI:** 10.1371/journal.pone.0353952.r002

16 Dec 2025

Response to Reviewers

Reviewer #1: Manuscript ID: PONE-D-25-46793

Manuscript title: Exploration of neuropeptides to identify potential target for regulating feeding behavior and development in Eurygaster integriceps

In this manuscript, authors performed a study of neuropeptides in Eurygaster integriceps Puton including sequence identification and expression profiling by means of quantitative PCR. Sequence features of precursors were analyzed in detail for 13 selected neuropeptides and their expressions were examined in various developmental stages, gender comparison and tissue preference (head vs gut). With these collected data, authors deduced and summarized their potential roles in the regulation of insect physiology. Overall, this is a continuation or companion study of their previously published results (reference 15 in this manuscript) whilst reference 15 was based on RNA-seq analysis of whole-body adult samples. All the neuropeptides described in this manuscript were identified in reference 15. This manuscript alone may stand technically sound after revisions, but the major challenge is to distinguish the scientific advancement from reference 15 in authors’ text descriptions. Reference 15 has described the potential roles of putative neuropeptides in physiological processes including stress and addiction, water balance, feeding, circadian rhythms, reproduction and development. The above-mentioned is my biggest concern for this submitted manuscript.

Response: We thank the reviewer for raising this important point. The previously published results only showed a general transcriptomic catalog of neuropeptide precursors from adult whole-body. In contrast, the current work represents a distinct and substantially expanded novel investigation through the following analyses:

• Targeted selection and structural validation of neuropeptides linked to feeding regulation.

• Developmental expression profiling across six life stages.

• Tissue-specific analysis of head vs. gut.

• Sex-specific comparison between male and female adults.

• Quantitative validation, which was not performed previously.

Further, we have also revised the Materials and Methods (Line 117 of revised version of manuscript) to include a new subsection (“Transcriptome data mining and peptide structural analysis”), clearly explaining how the earlier RNA-seq dataset was re-analyzed and expanded for this functional study.

Reviewer #1-1: Line 50, 46 neuropeptides were not identified in this manuscript. It only described 13 selected ones from reference 15.

Response#1-1: We agree that this manuscript does not identify 46 neuropeptides. Those 46 precursors were identified only in our previous RNA-seq study (Ref. 16). The present work specifically examines 13 neuropeptides selected for their documented roles in feeding regulation. To avoid confusion, we have revised the text to clearly reflect this focus. The updated sentence (Lines 46-50 of revised version of manuscript) now reads:

“In this study, we focused on 13 feeding-related neuropeptides previously identified from our whole-body RNA-seq dataset of Eurygaster integriceps. Using a combination of bioinformatics and expression analyses, we characterized precursor structures, predicted mature peptides, and quantified expression across developmental stages, between sexes, and in key feeding-associated organs of head and gut.”

Reviewer #1-2: Statistical analysis in materials and methods. How pair-wise analysis (Fig. S2 and S3) was performed is not described in materials and methods.

Response#1-2: We have now clarified the statistical procedures used for the pair-wise analyses shown in S2 Fig and S3 Fig. A detailed description of the methods— including the use of independent-samples t-tests for two-group comparisons and one-way ANOVA with Tukey’s HSD for multi-group comparisons—has been added to the Statistical Analysis section at Lines 185–192 of the revised manuscript.

Reviewer #1-3: Table S2 is a shortened form of Table 2 from reference 15. This is a duplicate publication of the same results.

Response#1-3: Thank you for the comment. Table S2 is not a shortened or duplicated form of Table 2 from Reference 16. Reference 16 was a general transcriptome survey that catalogued all predicted neuropeptide precursors in E. integriceps, but it did not include differential expression analyses, sex-specific comparisons, tissue-specific comparisons, or developmental profiling. In the present study, we focus exclusively on 13 neuropeptides that showed functional relevance to feeding and development based on our new expression analyses. Table S2 includes only these 13 sequences so that readers can easily reference the specific precursors examined in the qPCR experiments. Therefore, Table S2 is a study-specific subset created to support the new analyses presented here, and it does not duplicate the content of Table 2 in Reference 16.

Reviewer #1- 4-1. Comparing Table S2 (this manuscript) and Table 2 (reference 15), it is confusing that sequence lengths and signal peptide lengths are different for the following precursors, AKH1, AKH2, AKH3, Burs, Crz-2, ETH, NPF, sNPF and SIF. They have the same lists of gene IDs and homology analysis in two manuscripts.

Response#1-4-1: Thank you for raising this important point. We agree that several precursors (AKH1, AKH2, AKH3, Burs, Crz-2, ETH, NPF, sNPF, and SIF) show differences in sequence length and signal peptide length between Table S2 of the current manuscript and Table 2 of Reference 16, despite sharing the same gene IDs. These differences do not represent conflicting results but rather updated and more accurate annotations. In Reference 16, neuropeptide precursors were reported as part of a general transcriptome survey, and the ORF predictions were generated using automated pipelines that allowed multiple potential start codons. For the present study, we re-evaluated all 13 selected neuropeptide genes manually, applying a stricter annotation approach that uses the canonical methionine (ATG) start codon and validates signal peptide regions using dedicated prediction tools. This refinement was led to adjustments in the ORF boundaries for some precursors, which in turn produced updated total and signal peptide lengths. Thus, the sequences presented in Table S2 represent corrected and biologically refined precursor annotations, tailored specifically for the structural and expression analyses performed in this study. We have added a clarification in the revised manuscript to prevent confusion between the broad transcriptome-based predictions in Reference 16 and the updated, manually curated precursor sequences used here.

Reviewer #1-4-2: Also, why was Ast-C2 omitted for analysis in this study for the Ast family?

Response#1-4-2: In the present study, we selected only one Ast-C precursor for experimental validation because the two predicted Ast-C transcripts share highly similar precursor architectures and mature peptide regions. Therefore, including both would not have provided additional biological insight into this neuropeptide family. We prioritized a single representative Ast-C precursor for qPCR analysis to avoid redundancy and to focus resources on neuropeptides with distinct sequences and predicted functions. This clarification has now been added to the revised manuscript.

Reviewer #1-5: Fig. 6 and Fig. 7 look nice but are better replaced by Fig. S1, S2 and S3. Result description by means of Fig. 6 and Fig. 7 largely disregarded the statistical analyses in S1, S2 and S3

Response#1-5: We thank the reviewer for this helpful comment. Figures 6 and 7 serve as visual summaries, and the detailed statistical analyses validating these patterns are now explicitly referenced in Supplementary Figures S1–S3. We have added citations to these supplementary figures in Lines 304–305, 322–325, and 349–351 to clearly direct readers to the corresponding statistical support.

Reviewer #2: This manuscript presents an investigation into the neuropeptidome of the Sunn pest (Eurygaster integriceps), combining transcriptome mining, bioinformatics prediction, and RT-qPCR expression profiling across developmental stages, sexes, and tissues. The topic is relevant, given the interest in species-specific molecular targets for sustainable pest management.

However, while the research is promising, the manuscript would benefit from some rework in its presentation form. Several claims are descriptive, while the statistics are present in the supplement; bringing some of the information from the external file to the main text would benefit the readability and interpretation.

Response#2: We appreciate the reviewer’s insightful suggestion. To improve readability and strengthen the integration between descriptive results and statistical evidence, we have added explicit references to the supporting statistical analyses in Supplementary Figures S1–S3 within the Results section (Lines 304–305, 322–325, and 349–351). In addition, we incorporated clearer interpretation of stage-specific expression patterns into the Discussion (Lines 433–441), including examples such as the developmental fluctuations in PDF, ITP, and Allatostatin expression.

Reviewer #2-1: Specify the accession numbers for the RNA-seq dataset used.

Response#2-1: BioProject accession number PRJNA1015108 has now been stated in the manuscript (Line 120).

Reviewer #2-2: Provide software versions, parameter settings, and statistical methods used in analyses.

Response#2-2: The software tools and their versions (e.g., Oligo7 v7.60, Primer3 v2.6.1, ClustalX 2.1, WebLogo 2.8.2) are now specified in the Materials and Methods (Lines 131–132 and 168). Parameter settings and statistical methods have also been added, including one-way ANOVA with Tukey’s HSD (P < 0.05), as outlined in Lines 190–192, which now read: “For comparisons involving more than two groups (developmental stages), one-way ANOVA was conducted, followed by Tukey’s HSD post hoc test. Statistical significance was set at P < 0.05.”

Reviewer #2-3 Identify the reference genes used and provide validation data for their stability, and add a validation test of these gene across different developmental stages to the supplement.

Response#2-3: We appreciate the reviewer’s suggestion. Two reference genes (Actin and 18S rRNA) were evaluated for stability, and 18S rRNA showed the most consistent expression across developmental stages. The stability assessment—including mean, SD, SEM, and CV%—is now described in the Methods (Lines 175–181) and further supported in the Results (Lines 322–325). Complete validation data are provided in Supplementary Fig. S2 and Supplementary Table S4.

Reviewer #2-4 The Results are descriptive. Claims of higher or lower expression would benefit by the support of quantitative fold-change values

Response#2-4: We thank the reviewer for this constructive comment. Quantitative fold-change values have been added to support the reported expression patterns. The text now reads (Lines 433–441):

For Neuropeptide PDF, expression decreased to 0.3-fold in SI3 and subsequently increased to 3.7-fold in SI4. Similarly, Neuropeptide ITP showed a reduction to 0.4-fold in SI3 followed by an increase to 2.5-fold in SI4. These patterns suggest that neuropeptides may have stage-specific regulatory functions in E. integriceps. For example, Allatostatins Ast-A and Ast-B peaked during the first instar, with 2.1-fold and 8.1-fold increases relative to eggs, respectively, whereas Ast-C exhibited its highest expression in eggs. During nymphal stages, Ast-C expression was lower than in eggs, with fold-changes of 0.5, 0.3, 0.2, 0.4, and 0.2 in the first to fifth instars, respectively (S1 Fig), suggesting its role in early embryonic development rather than feeding regulation.

Reviewer #2-5 Indicate how normalization was performed (ΔΔCt relative to which calibrator stage or tissue).

Response#2-5: The normalization procedure (ΔΔCt method and the calibrators used) has now been fully clarified in the Materials and Methods. The text now reads (Lines 186–192):

" The relative gene expression levels (ΔCq values normalized to reference genes) were calculated for each sample, and results are presented as mean ± standard error (SE). Pairwise comparisons between two groups (male vs. female, and head vs. gut) were performed using independent-samples t-tests. For comparisons involving more than two groups (developmental stages), one-way analysis of variance (ANOVA) was conducted, followed by Tukey’s honestly significant difference (HSD) post hoc test. Statistical significance was set at P < 0.05. All analyses were performed using SPSS version 27.0 (IBM Corp., Armonk, NY, USA). "

Reviewer #2-6 Figures and tables in the supplementary material are essential for interpreting results. These could be partially summarized and moved into the main manuscript.

Response#2-6: We have revised the Results section to include concise summaries and direct references to the relevant supplementary figures and tables, ensuring clearer linkage between the main figures and the supporting statistical analyses.

Lines 322–325: A summary of reference gene stability, with explicit reference to Supplementary Fig. S2 and Supplementary Table S4.

Lines 348–351: A description of the organ-specific expression patterns shown in Fig. 7, with a direct reference to the supporting analyses in Supplementary Fig. S3.

Reviewer #3 reviewer comments from the PDF file

Reviewer #3: The reviewer suggested adding the phrase “In this study, we analyzed previously generated RNA-seq datasets” to Lines 48–49 (reviewer comment on pdf file).

Response: We appreciate this useful suggestion. We have revised the manuscript accordingly. The updated text now reads (Lines 46–48):

" In this study, we focused on 13 feeding-related neuropeptides previously identified from our whole-body RNA-seq dataset of Eurygaster integriceps. "

Reviewer #3: The reviewer requested adding brief quantitative indicators in Line 53 (reviewer comment on pdf file).

Response: This revision has been completed and incorporated into the manuscript (Line 55 of the revised version).

Reviewer #3: The reviewer noted that the Introduction lacks a clearly defined knowledge gap specific to E. integriceps. The reviewer also requested explicit acknowledgement that this study builds on a previous transcriptomic dataset, with clarification of what new analyses and insights are provided here (reviewer comment on pdf file).

Response: We appreciate the reviewer’s comment. In the revised manuscript (Introduction, Lines 105–111), we clarified the specific knowledge gap and explained how this study builds on our previously generated RNA-seq dataset. Specifically, the revised text now reads (Lines 105–111):

" Our earlier whole-body RNA-seq study [16] identified 46 neuropeptide precursors in Eurygaster integriceps, but it did not resolve developmental, sex-specific, or tissue-specific expression patterns. Building on this foundational dataset, the present study focuses on major feeding-related neuropeptides for detailed structural and functional analysis. We examined precursor architecture, predicted mature peptide structures, and quantified expression across developmental stages, between sexes, and in key feeding-associated tissues (head and gut) using gene expression profiling."

Moreover, we also specified in the Materials and Methods that the original transcriptomic data are publicly available in the SRA: Lines 118–120: " The transcriptomic resources used for peptide discovery and structural characterization in this study were derived from our previously published whole-body RNA-seq dataset of E. integriceps [16], available under BioProject accession PRJNA1015108"

Reviewer #3: The reviewer suggested adding information on current management practices and associated challenges (reviewer comment on pdf file(

Response: This revision has been completed in the revised manuscript (Lines 72–78). We added relevant details and cited a recent reference:

Duman, M., & Alaserhat, İ. (2025). Assessment of Eurygaster integriceps parameters to improve integrated pest management in Southeastern Türkiye wheat fields. Scientific Reports, 15(1), 26031.

Reviewer

Attachment Submitted filename: Response to Reviewers.docx

# Peer review: Decision Letter 1

**DOI:** 10.1371/journal.pone.0353952.r003

**Authors:** Humberto Lanz-Mendoza

3 Mar 2026

<div>PONE-D-25-46793R1

Exploration of neuropeptides to identify potential target for regulating feeding behavior and development in Eurygaster integriceps

PLOS One

Dear Dr. Ghaffari,

Thank you for submitting your manuscript to PLOS ONE. After careful consideration, we feel that it has merit but does not fully meet PLOS ONE’s publication criteria as it currently stands. Therefore, we invite you to submit a revised version of the manuscript that addresses the points raised during the review process.

==============================

1) Double-check all the gene expression interpretations across the text.

2) The Abstract states that several neuropeptides show higher expression in females. In the Results section, it is stated that these same neuropeptides show significantly higher expression in males, with only CCH and PDF being higher in females.

3) Several statements in the Results and Discussion imply functional roles. Please review these statements to show correlation or causation supported by evidence.

4) The Methods describe different numbers of individuals per developmental stage, but do not explicitly state whether samples were pooled prior to RNA extraction and qPCR. Please clarify .

==============================

Please submit your revised manuscript by Apr 17 2026 11:59PM. If you will need more time than this to complete your revisions, please reply to this message or contact the journal office at plosone@plos.org. When you're ready to submit your revision, log on to https://www.editorialmanager.com/pone/ and select the 'Submissions Needing Revision' folder to locate your manuscript file.

Please include the following items when submitting your revised manuscript:

- A letter that responds to each point raised by the academic editor and reviewer(s). You should upload this letter as a separate file labeled 'Response to Reviewers'.
- A marked-up copy of your manuscript that highlights changes made to the original version. You should upload this as a separate file labeled 'Revised Manuscript with Track Changes'.
- An unmarked version of your revised paper without tracked changes. You should upload this as a separate file labeled 'Manuscript'.

-->If you would like to make changes to your financial disclosure, please include your updated statement in your cover letter. Guidelines for resubmitting your figure files are available below the reviewer comments at the end of this letter.

If applicable, we recommend that you deposit your laboratory protocols in protocols.io to enhance the reproducibility of your results. Protocols.io assigns your protocol its own identifier (DOI) so that it can be cited independently in the future. For instructions see: https://journals.plos.org/plosone/s/submission-guidelines#loc-laboratory-protocols. Additionally, PLOS ONE offers an option for publishing peer-reviewed Lab Protocol articles, which describe protocols hosted on protocols.io. Read more information on sharing protocols at https://plos.org/protocols?utm_medium=editorial-email&utm_source=authorletters&utm_campaign=protocols.

We look forward to receiving your revised manuscript.

Kind regards,

Humberto Lanz-Mendoza

Academic Editor

PLOS One

Journal Requirements:

1. If the reviewer comments include a recommendation to cite specific previously published works, please review and evaluate these publications to determine whether they are relevant and should be cited. There is no requirement to cite these works unless the editor has indicated otherwise.

2. Please review your reference list to ensure that it is complete and correct. If you have cited papers that have been retracted, please include the rationale for doing so in the manuscript text, or remove these references and replace them with relevant current references. Any changes to the reference list should be mentioned in the rebuttal letter that accompanies your revised manuscript. If you need to cite a retracted article, indicate the article’s retracted status in the References list and also include a citation and full reference for the retraction notice.

Additional Editor Comments :

Please answer the reviewer's 2 comments, in particular:

1) Double-check all the gene expression interpretations across the text.

Specifically for the sex-specific interpretation:

2) The Abstract states that several neuropeptides (Ast-A, Ast-B, Ast-C, AKH, SIF, ITP, Burs, sNPF, NPF, Crz) show higher expression in females. In the Results section, it is stated that these same neuropeptides show significantly higher expression in males, with only CCH and PDF being higher in females.

3) Several statements in the Results and Discussion imply functional roles “indicating involvement in embryonic development” or “suggesting a regulatory role” based solely on transcript abundance. These conclusions should be softened to reflect correlation rather than causation, unless supported by functional assays.

4) The Methods describe different numbers of individuals per developmental stage (e.g., >100 eggs vs. 10 adults), but do not explicitly state whether samples were pooled prior to RNA extraction and qPCR. This should be clarified to avoid ambiguity in experimental design.

5) Statements such as “not detected in the gut” I would revise to “not detected above the threshold of detection” to avoid implying absolute absence.

[Note: HTML markup is below. Please do not edit.]

Reviewers' comments:

Reviewer's Responses to Questions

**Comments to the Author**

1. If the authors have adequately addressed your comments raised in a previous round of review and you feel that this manuscript is now acceptable for publication, you may indicate that here to bypass the “Comments to the Author” section, enter your conflict of interest statement in the “Confidential to Editor” section, and submit your "Accept" recommendation.

Reviewer #1: All comments have been addressed

Reviewer #2: All comments have been addressed

**********

2. Is the manuscript technically sound, and do the data support the conclusions?

The manuscript must describe a technically sound piece of scientific research with data that supports the conclusions. Experiments must have been conducted rigorously, with appropriate controls, replication, and sample sizes. The conclusions must be drawn appropriately based on the data presented.

Reviewer #1: (No Response)

Reviewer #2: Yes

**********

3. Has the statistical analysis been performed appropriately and rigorously?

Reviewer #1: (No Response)

Reviewer #2: Yes

**********

4. Have the authors made all data underlying the findings in their manuscript fully available?

The PLOS Data policy requires authors to make all data underlying the findings described in their manuscript fully available without restriction, with rare exception (please refer to the Data Availability Statement in the manuscript PDF file). The data should be provided as part of the manuscript or its supporting information, or deposited to a public repository. For example, in addition to summary statistics, the data points behind means, medians and variance measures should be available. If there are restrictions on publicly sharing data—e.g. participant privacy or use of data from a third party—those must be specified.

Reviewer #1: (No Response)

Reviewer #2: Yes

**********

5. Is the manuscript presented in an intelligible fashion and written in standard English?

PLOS ONE does not copyedit accepted manuscripts, so the language in submitted articles must be clear, correct, and unambiguous. Any typographical or grammatical errors should be corrected at revision, so please note any specific errors here.

Reviewer #1: (No Response)

Reviewer #2: Yes

**********

6. Review Comments to the Author

Please use the space provided to explain your answers to the questions above. You may also include additional comments for the author, including concerns about dual publication, research ethics, or publication ethics. (Please upload your review as an attachment if it exceeds 20,000 characters)

Reviewer #1: (No Response)

Reviewer #2: This revised manuscript improves the presentation of data on the comprehensive structural and expression analysis of 13 feeding-related neuropeptides in Eurygaster integriceps. The study is technically sound, the bioinformatic annotation is carefully executed, and the developmental, sex-specific, and tissue-specific expression profiling adds value beyond the authors’ previously published transcriptome dataset. Overall, the work fits within the scope of PLOS ONE and addresses an important pest species.

However, there are critical points of inconsistencies still present towards the manuscript, for example the sex-specific expression patterns are different state and interpret on different points of the manuscript like the abstract and results, due to this I would recommend double check all the gene expression interpretation across the text to confirm no other mistakes were made during writing.

Specifically for the sex-specific interpretation: The Abstract states that several neuropeptides (Ast-A, Ast-B, Ast-C, AKH, SIF, ITP, Burs, sNPF, NPF, Crz) show higher expression in females. While on the Results section it is stated that these same neuropeptides show significantly higher expression in males, with only CCH and PDF higher in females.

Several statements in the Results and Discussion imply functional roles “indicating involvement in embryonic development” or “suggesting a regulatory role” based solely on transcript abundance. These conclusions should be softened to reflect correlation rather than causation, unless supported by functional assays.

The Methods describe different numbers of individuals per developmental stage (e.g., >100 eggs vs. 10 adults), but do not explicitly state whether samples were pooled prior to RNA extraction and qPCR. This should be clarified to avoid ambiguity in experimental design.

Another point that needs attention on the writing and interpretation, statements such as “not detected in the gut” I would revise to “not detected above the threshold of detection” to avoid implying absolute absence.

Based on this I would recommend a revision of the text to check the inconsistencies, and after the issue is resolved, the manuscript will probably be suitable for publication.

**********

7. PLOS authors have the option to publish the peer review history of their article (what does this mean?). If published, this will include your full peer review and any attached files.

If you choose “no”, your identity will remain anonymous but your review may still be made public.

**Do you want your identity to be public for this peer review?**  For information about this choice, including consent withdrawal, please see our Privacy Policy.

Reviewer #1: No

Reviewer #2: No

**********

[NOTE: If reviewer comments were submitted as an attachment file, they will be attached to this email and accessible via the submission site. Please log into your account, locate the manuscript record, and check for the action link "View Attachments". If this link does not appear, there are no attachment files.]

To ensure your figures meet our technical requirements, please review our figure guidelines: https://journals.plos.org/plosone/s/figures

You may also use PLOS’s free figure tool, NAAS, to help you prepare publication quality figures: https://journals.plos.org/plosone/s/figures#loc-tools-for-figure-preparation.

NAAS will assess whether your figures meet our technical requirements by comparing each figure against our figure specifications.

# Author comment: Author response to Decision Letter 2

**DOI:** 10.1371/journal.pone.0353952.r004

21 Apr 2026

Response to Reviewers

PONE-D-25-46793R2

Reviewer #1-1: Double-check all the gene expression interpretations across the text.

Response #1-1: We appreciate the reviewer’s suggestion. Double-check and minor corrections have carefully been done all the gene expression interpretations across the text.

Reviewer #1-2: The Abstract states that several neuropeptides show higher expression in females. In the Results section, it is stated that these same neuropeptides show significantly higher expression in males, with only CCH and PDF being higher in females.

Response #1-2: We thank the reviewer for raising this mistake in the Abstract section. The expression level of Ast-A, Ast-B, Ast-C, AKH, SIF, ITP, Burs, sNPF, NPF, and Crz, were higher in males than in females, and the statement is correct as already mentioned in the Results section (according to S2 Fig). Now, we have corrected the writing mistake in the Abstract section (Revised version of manuscript, Lines 50-53). The text now it reads:

"Several neuropeptides, including Ast-A, Ast-B, Ast-C, AKH, SIF, ITP, Burs, sNPF, NPF, and Crz showed higher expression in males than in females, suggesting sexually dimorphic regulation of feeding and metabolism."

Reviewer #1-3: Several statements in the Results and Discussion imply functional roles. Please review these statements to show correlation or causation supported by evidence.

Response #1-3: We have added more references in the revised version of manuscript to show correlation or causation supported by evidence (Discussion section, Lines 484-486 and 506-507).

Reference [59]: Yao H, et al. The neuropeptide pigment-dispersing factor enhanced food intake and alleviated antifeedant effects by azadirachtin in Spodoptera frugiperda larvae. 2026;119: 1402–1411.

Reference [65]: Garelli A, et al. Body growth and shape regulation by neuropeptides and other neuroactive peptides in insects. Insect Sci. 2026;76: 1–9.

Reviewer #1-4: The Methods describe different numbers of individuals per developmental stage, but do not explicitly state whether samples were pooled prior to RNA extraction and qPCR. Please clarify.

Response #1-4: We thank the reviewer for this helpful comment. Now, we have explicitly stated in the revised version of manuscript. The revised text now it reads:

Lines 148-152 of revised manuscript: "For each stage we prepared a single pooled sample that comprised more than 100 eggs, 30 individuals for each of the 1st to 3rd instar nymphs, and 10 individuals for each of the 4th and 5th instar nymphs, female adults, and male adults. Samples were immediately frozen in liquid nitrogen and stored at −80 °C until further experiments."

"Line 158 of revised manuscript: "The pooled organs were pulverized in liquid nitrogen and stored at −80 °C prior to RNA extraction."

Attachment Submitted filename: Response_to_Reviewers_auresp_2.docx

# Peer review: Decision Letter 2

**DOI:** 10.1371/journal.pone.0353952.r005

**Authors:** Shashank Pandey

29 Jun 2026

PONE-D-25-46793R2

Exploration of neuropeptides to identify potential target for regulating feeding behavior and development in Eurygaster integriceps

PLOS One

Dear Dr. Mohammad Reza Ghaffari,

Thank you for submitting your manuscript to PLOS ONE. After careful consideration, we feel that it has merit but does not fully meet PLOS ONE’s publication criteria as it currently stands. Therefore, we invite you to submit a revised version of the manuscript that addresses the points raised during the review process.

===================================

Thank you for submitting your manuscript to PLOS ONE.

We invite you to submit a revised version of the manuscript that addresses the points raised during the review process which was related to biological replication in the RT-qPCR experiments. It can be seen from one reviewer's comments.

=================================

Please submit your revised manuscript by Aug 13 2026 11:59PM. If you will need more time than this to complete your revisions, please reply to this message or contact the journal office at plosone@plos.org. When you're ready to submit your revision, log on to https://www.editorialmanager.com/pone/ and select the 'Submissions Needing Revision' folder to locate your manuscript file.

Please include the following items when submitting your revised manuscript:

- A rebuttal letter that responds to each point raised by the academic editor and reviewer(s). You should upload this letter as a separate file labeled 'Response to Reviewers'.
- A marked-up copy of your manuscript that highlights changes made to the original version. You should upload this as a separate file labeled 'Revised Manuscript with Track Changes'.
- An unmarked version of your revised paper without tracked changes. You should upload this as a separate file labeled 'Manuscript'.

If you would like to make changes to your financial disclosure, please include your updated statement in your cover letter. Guidelines for resubmitting your figure files are available below the reviewer comments at the end of this letter.

If applicable, we recommend that you deposit your laboratory protocols in protocols.io to enhance the reproducibility of your results. Protocols.io assigns your protocol its own identifier (DOI) so that it can be cited independently in the future. For instructions see: https://journals.plos.org/plosone/s/submission-guidelines#loc-laboratory-protocols. Additionally, PLOS ONE offers an option for publishing peer-reviewed Lab Protocol articles, which describe protocols hosted on protocols.io. Read more information on sharing protocols at https://plos.org/protocols?utm_medium=editorial-email&utm_source=authorletters&utm_campaign=protocols.

As the corresponding author, your ORCID iD is verified in the submission system and will appear in the published article. PLOS supports the use of ORCID, and we encourage all coauthors to register for an ORCID iD and use it as well. Please encourage your coauthors to verify their ORCID iD within the submission system before final acceptance, as unverified ORCID iDs will not appear in the published article. Only the individual author can complete the verification step; PLOS staff cannot verify ORCID iDs on behalf of authors.

We look forward to receiving your revised manuscript.

Kind regards,

Shashank Pandey

Academic Editor

PLOS One

Journal Requirements:

If the reviewer comments include a recommendation to cite specific previously published works, please review and evaluate these publications to determine whether they are relevant and should be cited. There is no requirement to cite these works unless the editor has indicated otherwise.

Please review your reference list to ensure that it is complete and correct. If you have cited papers that have been retracted, please include the rationale for doing so in the manuscript text, or remove these references and replace them with relevant current references. Any changes to the reference list should be mentioned in the rebuttal letter that accompanies your revised manuscript. If you need to cite a retracted article, indicate the article’s retracted status in the References list and also include a citation and full reference for the retraction notice.

Additional Editor Comments (if provided):

Please provide high resolution figures.

[Note: HTML markup is below. Please do not edit.]

Reviewers' comments:

Reviewer's Responses to Questions

**Comments to the Author**

1. If the authors have adequately addressed your comments raised in a previous round of review and you feel that this manuscript is now acceptable for publication, you may indicate that here to bypass the “Comments to the Author” section, enter your conflict of interest statement in the “Confidential to Editor” section, and submit your "Accept" recommendation.

Reviewer #1: All comments have been addressed

Reviewer #2: All comments have been addressed

**********

2. Is the manuscript technically sound, and do the data support the conclusions?

The manuscript must describe a technically sound piece of scientific research with data that supports the conclusions. Experiments must have been conducted rigorously, with appropriate controls, replication, and sample sizes. The conclusions must be drawn appropriately based on the data presented.

Reviewer #1: (No Response)

Reviewer #2: Yes

**********

3. Has the statistical analysis been performed appropriately and rigorously?

Reviewer #1: (No Response)

Reviewer #2: No

**********

4. Have the authors made all data underlying the findings in their manuscript fully available?

The PLOS Data policy requires authors to make all data underlying the findings described in their manuscript fully available without restriction, with rare exception (please refer to the Data Availability Statement in the manuscript PDF file). The data should be provided as part of the manuscript or its supporting information, or deposited to a public repository. For example, in addition to summary statistics, the data points behind means, medians and variance measures should be available. If there are restrictions on publicly sharing data—e.g. participant privacy or use of data from a third party—those must be specified.

Reviewer #1: (No Response)

Reviewer #2: No

**********

5. Is the manuscript presented in an intelligible fashion and written in standard English?

PLOS ONE does not copyedit accepted manuscripts, so the language in submitted articles must be clear, correct, and unambiguous. Any typographical or grammatical errors should be corrected at revision, so please note any specific errors here.

Reviewer #1: (No Response)

Reviewer #2: Yes

**********

6. Review Comments to the Author

Please use the space provided to explain your answers to the questions above. You may also include additional comments for the author, including concerns about dual publication, research ethics, or publication ethics. (Please upload your review as an attachment if it exceeds 20,000 characters)

Reviewer #1: (No Response)

Reviewer #2: The authors have satisfactorily addressed the major scientific concerns raised during the previous review rounds. The manuscript now provides a coherent description of neuropeptide precursor structures and expression patterns across developmental stages, sexes, and tissues in Eurygaster integriceps. The study is primarily descriptive in nature and represents a valuable molecular resource for future investigations of neuropeptide function in this economically important pest species.

I have one remaining concern regarding the description of biological replication in the RT-qPCR experiments. In the Methods section, the authors indicate that pooled samples were prepared for each developmental stage, while elsewhere they state that three biological replicates were used. The wording could be interpreted as suggesting that only a single pooled sample was generated per stage, which would not support the inferential statistical analyses reported. My interpretation, however, is that the authors likely generated multiple biological replicates using pooled individuals and that the issue is primarily one of methodological description rather than a fundamental flaw in the experimental design. Please clarify

**********

7. PLOS authors have the option to publish the peer review history of their article (what does this mean?). If published, this will include your full peer review and any attached files.

If you choose “no”, your identity will remain anonymous but your review may still be made public.

**Do you want your identity to be public for this peer review?**  For information about this choice, including consent withdrawal, please see our Privacy Policy.

Reviewer #1: No

Reviewer #2: No

**********

[NOTE: If reviewer comments were submitted as an attachment file, they will be attached to this email and accessible via the submission site. Please log into your account, locate the manuscript record, and check for the action link "View Attachments". If this link does not appear, there are no attachment files.]

To ensure your figures meet our technical requirements, please review our figure guidelines: https://journals.plos.org/plosone/s/figures

You may also use PLOS’s free figure tool, NAAS, to help you prepare publication quality figures: https://journals.plos.org/plosone/s/figures#loc-tools-for-figure-preparation.

NAAS will assess whether your figures meet our technical requirements by comparing each figure against our figure specifications.

# Author comment: Author response to Decision Letter 3

**DOI:** 10.1371/journal.pone.0353952.r006

30 Jun 2026

Response to Reviewers

PONE-D-25-46793R3

Reviewer #2: The authors have satisfactorily addressed the major scientific concerns raised during the previous review rounds. The manuscript now provides a coherent description of neuropeptide precursor structures and expression patterns across developmental stages, sexes, and tissues in Eurygaster integriceps. The study is primarily descriptive in nature and represents a valuable molecular resource for future investigations of neuropeptide function in this economically important pest species.

I have one remaining concern regarding the description of biological replication in the RT-qPCR experiments. In the Methods section, the authors indicate that pooled samples were prepared for each developmental stage, while elsewhere they state that three biological replicates were used. The wording could be interpreted as suggesting that only a single pooled sample was generated per stage, which would not support the inferential statistical analyses reported. My interpretation, however, is that the authors likely generated multiple biological replicates using pooled individuals and that the issue is primarily one of methodological description rather than a fundamental flaw in the experimental design. Please clarify

Response #2-1: We sincerely thank the reviewer for the careful evaluation of our manuscript and for the positive comments on the revisions. We appreciate the reviewer’s important observation regarding the description of biological replication in the RT-qPCR experiments. To clarify, for each developmental stage, sex, and tissue type, we prepared three independent biological replicates, each consisting of a separate pooled sample of multiple individuals. Therefore, pooled sampling was used within each replicate to obtain sufficient RNA and reduce individual variability, but replication was maintained through independently prepared pools. This ensures that the statistical analyses performed are supported by true biological replication. We have revised the Methods section accordingly. The revised text now reads (Lines 148-156 of the revised version of the manuscript):

"For each stage three independent biological replicates were prepared. Each biological replicate consisted of a separate pooled sample of multiple individuals, including more than 100 eggs, 30 individuals for each of the 1st to 3rd instar nymphs, and 10 individuals for each of the 4th and 5th instar nymphs, as well as female and male adults. Samples were immediately frozen in liquid nitrogen and stored at −80 °C until further experiments.

For organ-specific analysis, female and male adults were dissected to isolate heads and guts. For each tissue type, three independent biological replicates were prepared, each consisting of pooled samples (15 heads or 15 guts per replicate)."

Attachment Submitted filename: Response to Reviewer.docx

# Editor report: Decision Letter 3

**DOI:** 10.1371/journal.pone.0353952.r007

**Authors:** Shashank Pandey

2 Jul 2026

Exploration of neuropeptides to identify potential target for regulating feeding behavior and development in Eurygaster integriceps

PONE-D-25-46793R3

Dear Dr. Ghaffari,

We’re pleased to inform you that your manuscript has been judged scientifically suitable for publication and will be formally accepted for publication once it meets all outstanding technical requirements.

Within one week, you’ll receive an e-mail detailing the required amendments. When these have been addressed, you’ll receive a formal acceptance letter and your manuscript will be scheduled for publication.

An invoice will be generated when your article is formally accepted. Please note, if your institution has a publishing partnership with PLOS and your article meets the relevant criteria, all or part of your publication costs will be covered. Please make sure your user information is up-to-date by logging into Editorial Manager at Editorial Manager® and clicking the ‘Update My Information' link at the top of the page. For questions related to billing, please contact billing support.

If your institution or institutions have a press office, please notify them about your upcoming paper to help maximize its impact. If they’ll be preparing press materials, please inform our press team as soon as possible -- no later than 48 hours after receiving the formal acceptance. Your manuscript will remain under strict press embargo until 2 pm Eastern Time on the date of publication. For more information, please contact onepress@plos.org.

Kind regards,

Shashank Pandey

Academic Editor

PLOS One

Additional Editor Comments (optional):

Reviewers' comments:

# Editor report: Acceptance letter

**DOI:** 10.1371/journal.pone.0353952.r008

**Authors:** Shashank Pandey

PONE-D-25-46793R3

PLOS One

Dear Dr. Ghaffari,

I'm pleased to inform you that your manuscript has been deemed suitable for publication in PLOS One. Congratulations! Your manuscript is now being handed over to our production team.

At this stage, our production department will prepare your paper for publication. This includes ensuring the following:

* All references, tables, and figures are properly cited

* All relevant supporting information is included in the manuscript submission,

* There are no issues that prevent the paper from being properly typeset

You will receive further instructions from the production team, including instructions on how to review your proof when it is ready. Please keep in mind that we are working through a large volume of accepted articles, so please give us a few days to review your paper and let you know the next and final steps.

Lastly, if your institution or institutions have a press office, please let them know about your upcoming paper now to help maximize its impact. If they'll be preparing press materials, please inform our press team within the next 48 hours. Your manuscript will remain under strict press embargo until 2 pm Eastern Time on the date of publication. For more information, please contact onepress@plos.org.

You will receive an invoice from PLOS for your publication fee after your manuscript has reached the completed accept phase. If you receive an email requesting payment before acceptance or for any other service, this may be a phishing scheme. Learn how to identify phishing emails and protect your accounts at https://explore.plos.org/phishing.

If we can help with anything else, please email us at customercare@plos.org.

Thank you for submitting your work to PLOS ONE and supporting open access.

Kind regards,

PLOS ONE Editorial Office Staff

on behalf of

PhD Shashank Pandey

Academic Editor

PLOS One
